# Closing the Gap in Early Mathematics: Domain and Cognitive Insights from TIMSS 2023 in South Africa and Singapore

**DOI:** 10.12688/f1000research.172015.1

**Published:** 2025-11-05

**Authors:** Mathelela Steyn Mokgwathi

**Affiliations:** 1 Department of Early Childhood Education, University of South Africa, Pretoria, Gauteng, 0003, South Africa

**Keywords:** Trends in International Mathematics and Science Study (TIMSS), Mathematics Achievement, Content and Cognitive Domains, South Africa, Singapore

## Abstract

**Background:**

South Africa continues to underperform in primary mathematics, with foundational gaps evident across content and cognitive skills. This study investigates domain-specific patterns of achievement and cognitive demand using the *Trends in International Mathematics and Science Study (TIMSS) 2023*, benchmarking South African Grade 5 learners against Singaporean Grade 4 learners.

**Methods:**

A quantitative secondary analysis was conducted on nationally representative TIMSS 2023 datasets comprising 10,424 South African learners from 285 schools and 6,530 Singaporean learners from 181 schools. Mathematics achievement was disaggregated by content domains (Number, Measurement and Geometry, Data) and cognitive domains (Knowing, Applying, Reasoning). Weighted estimates, mean differences, and effect sizes (Cohen’s d) were computed to characterise performance gaps within and between countries.

**Results:**

South African learners scored well below the international centre point across all content and cognitive domains. The largest content gap with Singapore occurred in Measurement and Geometry, indicating persistent weaknesses in spatial reasoning. We observed the widest disparity in the cognitive domain of knowing, which reflected fragile factual knowledge and procedural fluency. South African learners showed relatively stronger performance in applying mathematical concepts, indicating some competence with routine and structured tasks; however, their deficits in knowing hindered their progression to reasoning, particularly in multi-step and non-routine problem-solving, which were the weakest areas.

**Conclusions:**

Early mathematics learning in South Africa is characterised by weak foundational knowledge, underdeveloped spatial reasoning, and limited opportunities for higher-order thinking. Targeted reforms should improve number and fact fluency, provide better teaching support for geometry and measurement, and add learning tasks that build on what learners are good at to help them think more clearly. Aligning curriculum intent, teacher development, and assessment expectations is essential to close domain-specific gaps and advance equitable foundational learning outcomes.

## Introduction

Mathematics achievement at the primary school level has profound implications for learners’ future participation in education, work, and society. Basic mathematics skills help with higher-order thinking, problem-solving, and learning for life, which are all important for both academic success and social participation ( Mullis et al., 2020). TIMSS has become a critical assessment instrument for benchmarking mathematical learner achievement internationally, revealing how national curricula, teaching practices, and educational systems prepare learners for cognitive demands in mathematics. South Africa has participated in TIMSS since 1995 and, despite modest gains, remains one of the lowest-performing systems globally ( Zuze et al., 2018). Much of the existing research focuses on Grade 9 data, emphasising long-term learning deficits and systemic inequities ( Mensah & Baidoo-Anu, 2022; Reddy & Hannan, 2019). Far less attention has been given to the primary phase, particularly Grade 5, where learners consolidate fundamental numeracy and begin to transition from concrete to abstract reasoning. TIMSS 2023 results indicate that South African Grade 5 learners achieved an average score of 362 compared to Singapore’s Grade 4 average of 615 (TIMSS, 2023). This 250-point gap, despite Singaporean learners being a grade lower than South African learners, signals deep foundational weaknesses in South Africa’s education system, inclusive of mathematics education.

National assessments such as the Annual National Assessments (ANA), which is now discontinued, and systemic evaluation reports have highlighted low achievement levels in mathematics among learners, but they rarely explore *how* learners perform across specific content and cognitive domains. Yet TIMSS distinguishes between three content domains (numbers, measurement and geometry, and data) and three cognitive domains (knowing, applying, and reasoning). Assessing learner achievement through these lenses allows for a diagnostic understanding of learners’ strengths and weaknesses. Research from high-performing education systems such as Singapore demonstrates that consistent curriculum alignment ( Johnson et al., 2020), spiral progression, and scaffolded teaching and learning cultivate a balanced development of knowledge, application, and reasoning skills ( Choy & Dindyal, 2024; Low & Wong, 2021; Morony, 2023; Mullis et al., 2020). Conversely, South African studies point to persistent challenges in geometry, reasoning, and teacher content knowledge ( Maqoqa, 2024; Taylor, 2021), compounded by curriculum overload and large class sizes that limit opportunities for formative assessment and conceptual engagement. Improving mathematics learner achievement therefore requires more than curriculum reform; it depends on strengthening the consistency between curriculum design and development, teacher professional development, and classroom practice. Knowing how learners interact with the content and cognitive demands offers crucial understanding of the areas that require the most instructional support and pedagogical innovation.

This study addresses these gaps by analysing South African Grade 5 learners’ performance at TIMSS 2023 relative to Singaporean Grade 4 learners. It contributes in three key ways. Firstly, it focuses on the under-researched area of early mathematics learning in Early Childhood Education (ECE), encompassing both formal and informal approaches, which aim to establish basic mathematics skills. Second, it disaggregates performance across content and cognitive domains to identify specific patterns of strength and weakness. Third, it links these results to curriculum and pedagogical implications, proposing strategies to strengthen basic knowledge, geometry teaching, and teacher professional development. The study was guided by the following research questions:

### Research questions

1. What are the patterns of South African Grade 5 learners’ performance across the TIMSS 2023 mathematics content domains (numbers, measurement and geometry, and data), and how do these compare with Singaporean Grade 4 learners?
2. How do South African learners perform across the TIMSS 2023 cognitive domains (Knowing, Applying, and Reasoning), and what specific strengths and weaknesses are revealed in comparison with Singapore?
3. What curriculum and pedagogical implications can be drawn from the comparative analysis of domain-specific and cognitive performance to inform strategies for improving mathematics achievement in South Africa?

### Literature review: Comparative analysis of South African and Singaporean grade 5 mathematics achievement

**Benchmarking with TIMSS 2023**

The Trends in International Mathematics and Science Study (TIMSS) serves as an international benchmark to evaluate the mathematical learner achievement of both primary and secondary school learners. Singapore was consistently ranked as the top achiever, with learners assessed at Grade 4 achieving an overall average of 615 points, while South Africa, assessed at Grade 5, scored 362 points. This means that Singaporean learners who are on average a year younger still outperform South African learners by more than 250 points ( von Davier et al., 2024). The magnitude of this achievement gap underscores the need to analyse not just overall scores but also performances across content domains (numbers, measurements, geometry, and data) and cognitive domains (knowing, applying, and reasoning) to understand how curricula and teaching practices shape outcomes.

**Curriculum alignment and content domains**

Singapore’s mathematical curriculum is internationally recognised for its coherence and spiral structure, which involves systematically revisiting concepts at increasing levels of complexity. In TIMSS 2023, Singapore scored 613 in numbers, 619 in measurement and geometry, and 616 in data, while South Africa scored 362, 353, and 362, respectively. Maqoqa (2024) and Tachie (2020) reported that the largest achievement gap is in measurement and geometry (266 points), an area long identified as a “blind spot” in South African classrooms. These gaps suggest that South African learners struggle with reasoning and geometric domains, while Singaporean learners benefit from early exposure to concrete manipulatives, reasoning, and visual models that build conceptual skills and knowledge.

**Cognitive demands: Knowing, applying, and reasoning**

TIMSS distinguishes between three cognitive domains: knowing, applying, and reasoning. Singapore’s Grade 4 learners achieved 624 in knowing, 615 in applying, and 609 in reasoning, whereas South Africa’s Grade 5 learners scored 357, 366, and 363. This result reveals a profound weakness in knowing (–267 points compared to Singapore), which reflects learners’ difficulties with knowing domains. South African learners performed slightly better in the applying domain (366) relative to their average, suggesting that when knowledge is available, learners can engage in routine applications. The reasoning domain, on the other hand, shows a persistent weakness. This means that South African learners are not being prepared for non-routine, multi-step problem-solving tasks, which is a strong point of the Singaporean system.

### Instructional and structural factors

Several systemic factors reinforce these disparities. According to Meier and West (2020), South Africa’s classrooms often suffer from overcrowding, with class sizes averaging over 50 learners, which hinders formative feedback and personalised support. Teacher content and pedagogical knowledge remain uneven, particularly in geometry and measurement ( Bhagwonparsadh & Pule, 2024; Taylor, 2021). In contrast, Singapore invests heavily in sustained teacher development, smaller class sizes, and instructional leadership, creating a conducive learning environment where consistent teaching and learning take place. Furthermore, although South Africa’s curriculum aims for comprehensive coverage, it has faced criticism for being “overloaded” and not allowing adequate time for the mastery of fundamental skills ( Milne & Mhlolo, 2021). By contrast, Singapore’s Concrete–Pictorial–Abstract (CPA) approach deliberately scaffolds learning so that conceptual understanding precedes abstraction, enabling a positive learner achievement in higher-order reasoning ( Leong et al., 2015; Lutfi & Dasari, 2024).

### Curriculum–Cognitive alignment in International research

International evidence further illustrates how disparities between curriculum objectives and classroom practices shape academic learner achievement. Yılmaz et al. (2021) found that, while mathematics curricula emphasised reasoning, textbook activities leaned more toward application, creating a mismatch between intended and taught cognitive emphases. Similarly, Bulut and Taşpınar-şener (2023) reported that the application domain is most frequently prioritised in the secondary mathematics curriculum, disregarding the ECE curriculum, whereas the emphasis placed on knowledge and reasoning differs across grade levels. In primary schools, Pertiwi and Wahidin (2020) showed that fourth-grade assessments are dominated by number-related content, with far less attention given to geometry or data activities. These results reflect TIMSS’s framework, where knowing entails factual recall and procedural fluency, applying involves transferring knowledge to structured contexts, and reasoning requires non-routine problem-solving and critical thinking ( Peduk & Ateş, 2019). Importantly, the study also found that content domains exert a more positive effect on mathematics learner achievement than cognitive domains.

This body of research provides an explanatory lens for South Africa’s TIMSS 2023 mathematics learner achievement. The relative strengths of South African Grade 5 learners in the application domain, alongside persistent weaknesses in knowledge and reasoning, reflect the misalignment noted by several studies internationally, where instruction and textbooks emphasise routine application but fail to develop the basic knowledge and reasoning capacity required for learner progression. In contrast, Singapore’s balanced curriculum and pedagogy demonstrate how alignment across content and cognitive domains fosters sustained learner achievement.

### TIMSS: A diagnostic instrument

TIMSS offers a diagnostic lens to evaluate the effectiveness of the current curriculum and teaching practices, rather than viewing the legacies of apartheid inequalities as the main reason for the ongoing poor achievement in mathematics among learners. The comparative analysis with Singapore illustrates that South Africa’s curriculum is aligned superficially in content but misaligned in cognitive expectations, particularly at the levels of knowing and reasoning. This mismatch leaves learners underprepared for both academic progression and broader applications of mathematics in their everyday lives. The fact that Singapore’s Grade 4 learners significantly outperform South Africa’s Grade 5 learners further shows that the gap in mathematics achievement among learners is not simply attributable to learner age or exposure but to differences in curriculum coherence, cognitive scaffolding, and teacher training and development.

The TIMSS 2023 results highlight not just the scale of South Africa’s underperformance but also its domain-specific and cognitive weaknesses. While Singapore demonstrates how curriculum coherence and sustained teacher training and development can support an understanding of teaching and learning across content and cognitive domains, South Africa’s challenges are rooted in weak foundations, gaps in geometry and measurement, and systemic barriers in teacher training, development, and the classroom environment. Addressing these challenges requires targeted curriculum reforms in ECE, early-grade numeracy, curriculum streamlining, and teacher professional development, with a particular emphasis on geometry, critical thinking (reasoning), and basic knowledge. Context-sensitive adaptations, rather than comprehensive importation of Singapore’s model, offer a pathway for South Africa to strengthen learner trajectories in mathematics.

### Conceptual framework

This study was guided by the TIMSS conceptual model, which distinguishes between mathematical content domains (numbers, measurement, geometry, and data) and cognitive domains (knowing, applying, and reasoning) ( Mullis et al., 2020). These dual dimensions provide a diagnostic lens for understanding not only what mathematics learners are expected to know but also how they engage with mathematical activities in the classroom. Therefore, the researcher can investigate South Africa’s historically poor mathematics learner achievement by identifying specific weaknesses in basic knowledge (knowing), conceptual understanding (applying), and higher-order reasoning.

To explain these disparities, the study draws on curriculum alignment theory, which emphasises coherence between curricular intentions, classroom practices, and assessment demands ( Porter, 2002). High-performing education systems such as Singapore demonstrate strong alignment through a spiral curriculum that revisits concepts at increasing levels of complexity supported by the Concrete–Pictorial–Abstract (CPA) approach that scaffolds learning from concrete manipulatives to abstract reasoning ( Leong et al., 2015; Lutfi & Dasari, 2024). In contrast, research in South Africa highlights curriculum overload, insufficient time for mastery of basic domains, and persistent weaknesses in geometry and spatial reasoning, exacerbated by gaps in teacher content knowledge ( Maqoqa, 2024; Taylor, 2021).

**Figure 1.**
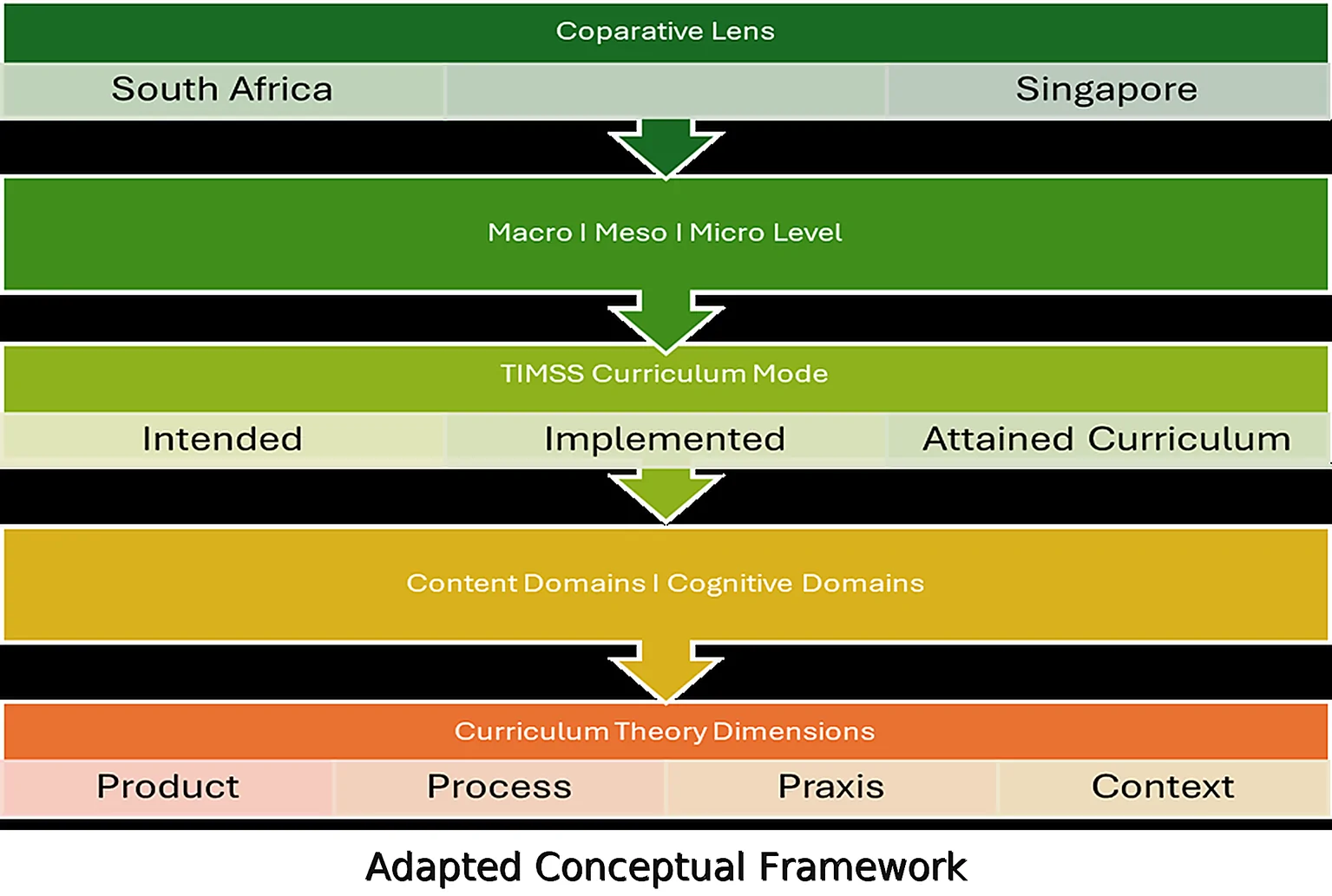
Adapted conceptual framework combining the comparative lens, TIMSS curriculum model, and curriculum theory dimensions guiding the study.

 Situating South Africa’s Grade 5 TIMSS 2023 results against Singapore’s Grade 4 performance thus allows for a comparative curriculum lens that identifies both where South African learners struggle most and how Singapore’s curriculum scaffolds progression from knowledge to application and reasoning. To guide this analysis, the study therefore adopted an integrative conceptual framework that combines the Comparative Lens (South Africa versus Singapore, considered at macro, meso, and micro levels) with the TIMSS Curriculum Model (distinguishing intended, implemented, and attained curricula, as well as content and cognitive domains) ( Alemu et al., 2021; Mullis et al., 2020) and Curriculum Theory Dimensions (alignment, spiral progression, CPA scaffolding as described by Lutfi and Dasari (2024), and Grundy’s (1992) product–process–praxis–context typology). Together, these components ensure that the framework is both diagnostic, by mapping learner strengths and weaknesses, and explanatory, by linking performance to curriculum and pedagogy, while also generating practical insights for educational reform. Figure 1 illustrates this integrative conceptual framework, showing how the comparative, curricular, and theoretical dimensions interact to guide the study’s analysis.

Building on this conceptual framework, the study’s methodology was designed to operationalise these dimensions through an analysis of the TIMSS 2023 data. The TIMSS curriculum model provided the basis for investigating both content and cognitive domains, while the comparative lens allowed for benchmarking South African Grade 5 learners against Singaporean Grade 4 learners. Curriculum theory informed the interpretive aspect of the analysis by connecting observed performance trends to overarching concerns of curriculum alignment, learner advancement, and pedagogical practices. The following section outlines the research design, participants, data collection, analytical strategy, and ethical considerations employed in this study.

## Methodology

### Research design

This study employed a quantitative secondary data analysis design, using data from the Trends in International Mathematics and Science Study (TIMSS) 2023. This design is particularly appropriate for investigating the mathematics achievement of South African Grade 5 learners across content and cognitive domains for several reasons. First, TIMSS provides a large, internationally standardised dataset that is both rigorous in design and nationally representative, making it suitable for examining learners’ performance patterns with a high degree of reliability. Second, secondary analysis enables the use of TIMSS’s robust psychometric procedures, including item response theory and plausible values, which strengthen the validity of inferences about learners’ achievement. Third, the TIMSS framework allows for meaningful international benchmarking, making it possible to situate South Africa’s performance in relation to high-performing education systems such as Singapore. Guided by the TIMSS assessment framework, the analysis focused on three content domains (numbers, measurement, geometry, and data) and three cognitive domains (knowing, applying, and reasoning). This two-fold focus not only enabled a diagnostic assessment of learners’ strengths and weaknesses within the South African context but also provided comparative insights to inform curriculum and policy reform globally.

### Participants

The South African Grade 5 sample for TIMSS 2023 consisted of approximately 10,424 learners drawn from 285 schools ( Department of Basic Education, 2024), while Singapore sampled 6530 Grade 4 learners from 181 schools ( von Davier et al., 2024). The International Association for the Evaluation of Educational Achievement (IEA), in collaboration with Statistics Canada, utilised a two-stage stratified cluster sampling methodology to guarantee nationally representative estimates ( Siegel & Foy, 2024). In the first stage, schools were selected with probabilities proportional to their size, and in the second stage, intact Grade 5 classes were sampled. The stratification variables included the school sector (public or private), the language of instruction, the geographic region, socioeconomic indicators, the degree of urbanisation, and prior academic achievements (Ibid.). This rigorous design ensured that the results accurately reflect the diversity of the South African education system and provide robust population-level estimates of learner performance.

### Data collection and analysis

Learners completed mathematics assessments together with contextual background questionnaires. The TIMSS mathematics achievement is measured on a scale with a standard deviation of 100 and an international centre point of 500, which makes it possible to make valid comparisons between countries. According to Reynolds (2024), the mathematics assessment comprised 183 items distributed across the three content domains (numbers: 94 items; measurement and geometry: 49 items; data: 40 items) and three cognitive domains (knowing: 58 items; applying: 85 items; reasoning: 40 items). Each student took one test booklet, and the results were estimated using item response theory and reported as plausible values ( von Davier, 2019). This design ensures reliable population-level estimates while reducing respondent burden.

The analysis concentrated on South Africa’s Grade 5 results, using Singapore’s Grade 4 outcomes as a benchmark to emphasise comparative mathematics learner achievement. Weighted descriptive statistics were computed according to IEA guidelines for handling complex sampling. These weights take into account the hierarchical structure of TIMSS, which makes sure that the estimates are not biassed at the national level ( Siegel & Foy, 2024). Differences in performance across domains were tested for statistical significance at *p* < 0.01. In addition, effect sizes (Cohen’s *d*) were calculated to assess the practical significance of observed gaps between content and cognitive domains and between South Africa and Singapore. This combination of statistical significance and effect size estimation provided a more profound understanding of performance trends.

### Ethical considerations

This study is based on secondary analysis of TIMSS 2023 restricted-use datasets provided by the IEA. The data contain no personal identifiers and were collected under strict international ethical protocols during the original administration. Because this research involved secondary analysis of anonymised data, no institutional ethics approval was required. There was no formal request to use the dataset from the IEA since the data is available in the public domain, and all analyses adhered to its guidelines for responsible data use.

## Results

### Content domain achievement

Table 1 reports the mean scores for South African Grade 5 learners and Singaporean Grade 4 learners across the three TIMSS mathematics content domains. The results reveal consistently lower learner achievement for South Africa across all three areas, with the largest gap evident in measurement and geometry. This persistent weakness in spatial reasoning reflects long-standing challenges in South African classrooms and emphasises the need for targeted curricula and pedagogical reforms in this domain that are context-sensitive.

**Table 1.** Mean scale scores and effect sizes in mathematics content domains, TIMSS 2023.

Content domain	Singapore (Grade 4)	South Africa (Grade 5)	Gap	Cohen’s *d*
Numbers	613	362	251	2.51
Measurement & Geometry	619	353	266	2.66
Data	616	362	254	2.54

All three domains reveal large effect sizes ( *d* > 2.5), signifying profound disparities. The most pronounced gap lies in Measurement and Geometry ( *d* = 2.66), confirming that South African learners face persistent difficulties in spatial reasoning and geometric concepts.

### Cognitive domain achievement

Table 2 presents performance across the TIMSS cognitive domains. The results show that South African learners are much less knowledgeable than their Singaporean peers in all three areas. The biggest difference is in the knowing domain. This result points to fragile foundations in knowing domain, which limit learners’ ability to advance toward higher-order reasoning activities. Although performance in the applying domain is relatively positive, it remains well below international benchmarks, suggesting that gains in procedural applications are insufficient without deeper conceptual mastery.

**Table 2.** Mean scale scores and effect sizes in mathematics cognitive domains, TIMSS 2023.

Cognitive domain	Singapore (Grade 4)	South Africa (Grade 5)	Gap	Cohen’s *d*
Knowing	624	357	267	2.67
Applying	615	366	249	2.49
Reasoning	609	363	246	2.46

The knowing domain shows the largest gap ( *d* = 2.67), underscoring fragile foundations in factual knowledge and procedural fluency. South African learners’ relatively positive achievement in applying (366) suggests that when basic knowledge is accessible, learners can engage with routine procedures. However, the consistent deficits across domains indicate that foundational gaps constrain progression to reasoning tasks, where Singaporean learners excel.

### Item-level illustrations

To illustrate the cognitive demands underlying these results, examples from released TIMSS items are useful. In the knowing domain (numbers), more than 90% of Singaporean learners were able to recall multiplication facts correctly, while fewer than 40% of South African learners were able to do the same. This shows that there are big gaps in basic fact fluency. In the applying domain (data), routine tasks such as interpreting a simple bar chart were accessible to many South African learners, suggesting some competence with structured and familiar problems. However, in the reasoning domain (geometry), where items demanded multi-step reasoning with angles, most Singaporean learners responded correctly compared to fewer than 20% of South African learners, reflecting limited exposure to non-routine problem-solving beyond procedural recall. Collectively, these examples highlight how curriculum exposure and classroom instructional practices shape learners’ preparedness to engage with domain-specific cognitive demands.

### Visualising the gaps

**Figure 2.**
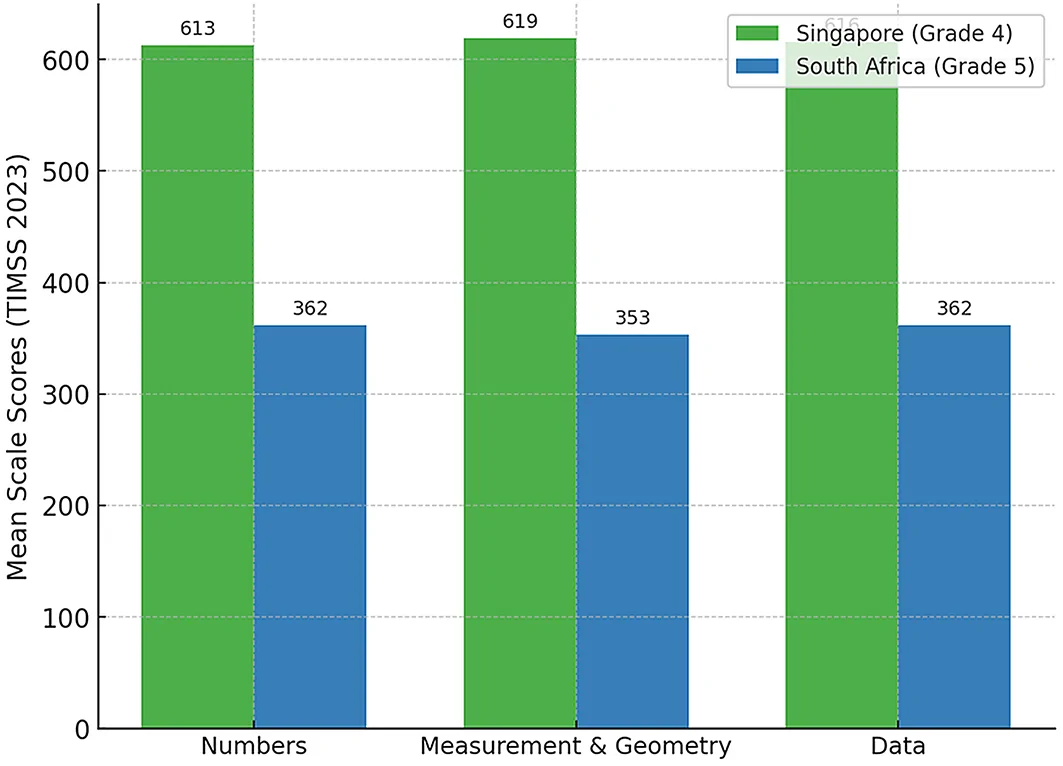
Comparative performance of South African Grade 5 and Singaporean Grade 4 learners across TIMSS content domains (Numbers, Measurement & Geometry, and Data).

Figure 2 illustrates the comparative performance of South African Grade 5 and Singaporean Grade 4 learners across the three TIMSS content domains. The visualisation confirms the consistently lower achievement of South African learners, with the largest gap observed in measurement and geometry. This finding supports the tabulated results and highlights the ongoing challenges that South African learners encounter in spatial reasoning and geometric concepts. By presenting the disparities graphically,  Figure 2 highlights the structural nature of these weaknesses and the extent to which curriculum design and instructional practices shape domain-specific performance.

**Figure 3.**
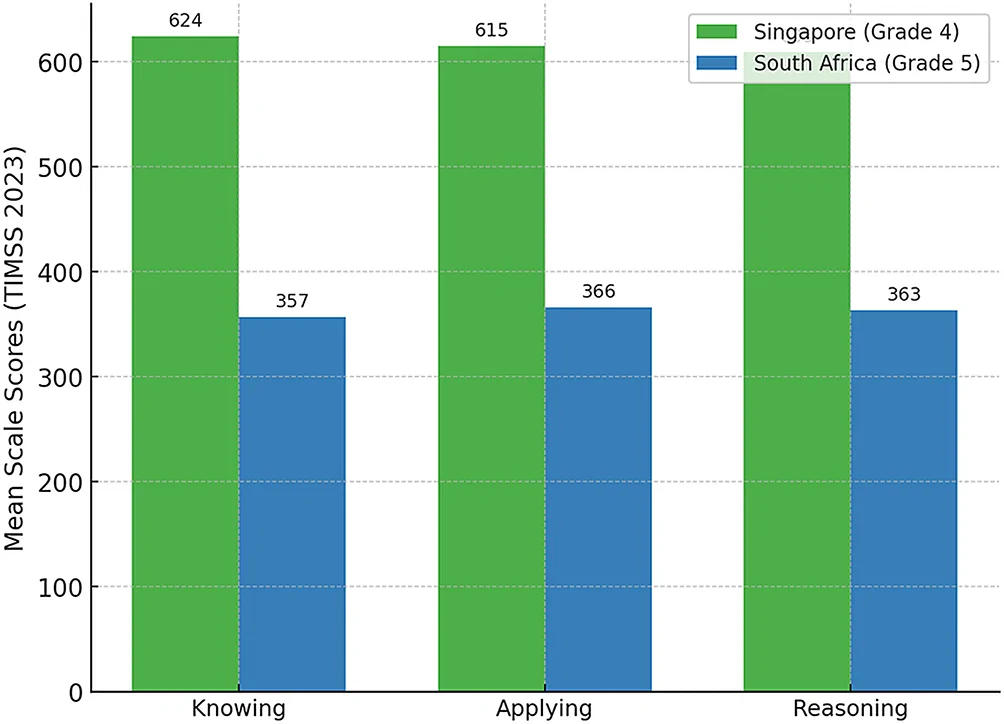
Comparative performance of South African Grade 5 and Singaporean Grade 4 learners across TIMSS cognitive domains (Knowing, Applying, and Reasoning).

Figure 3 presents the comparative performance of South African Grade 5 and Singaporean Grade 4 learners across the TIMSS cognitive domains. The figure makes clear that the widest disparity lies in the knowing domain, where South African learners demonstrate severe deficits in foundational knowledge and procedural fluency. While relative performance in applying appears slightly positive, it remains far below international benchmarks, limiting progress to more complex reasoning. These results show how gaps in basic knowledge can lead to fewer chances for higher-order thinking. On the other hand, Singapore’s curriculum scaffolding helps all three cognitive domains grow in a balanced way.

### Summary of results

The results of the analysis highlight three critical insights into South African learners’ mathematics achievement. First, geometry and spatial reasoning continue to represent the weakest domain, pointing to enduring structural gaps in both curriculum design and teacher preparation. Second, the severity of deficits in foundational knowledge, reflected in the knowing domain, restricts learners from progressing toward higher-order reasoning tasks. Third, although learners demonstrate relatively positive learner achievement in the applying domain, this potential remains constrained by the absence of solid basic skills and limited opportunities for reasoning skills, which prevents the development of sustained mathematical learner achievement. Collectively, these results suggest that South Africa’s performance challenges are not incidental but rather systematic, domain-specific, and deeply embedded in pedagogical practices. In contrast, the comparison with Singapore emphasises the value of curriculum coherence and deliberate cognitive scaffolding for supporting learners’ steady progression across domains.

## Discussion

The TIMSS 2023 results reaffirm that South African Grade 5 learners perform significantly below the international mathematics benchmark, achieving a mean score of 362 compared with the centre point of 500. While this overall result underscores persistent systemic challenges, disaggregating performance across content and cognitive domains provides a more diagnostic understanding of where learning gaps emerge and how they shape curriculum, pedagogy, and teacher preparation.

At the *content level*, learner performance in Number and Data suggests partial competence in basic computation and routine interpretation tasks. However, the marked deficit in measurement and geometry exposes structural barriers to developing spatial reasoning and conceptual understanding. These results confirm earlier evidence that geometry remains a “blind spot” in South African classrooms, often attributed to weak pedagogical content knowledge, crowded curricula, and limited use of concrete or visual learning tools ( Maqoqa, 2024; Taylor, 2019, 2021). Pedagogically, early mathematics instruction continues to privilege procedural accuracy over conceptual exploration. Empowering teachers to adopt visual, contextual, and manipulative-based approaches through dynamic geometry software, outdoor measurement tasks, and locally available materials could enhance conceptual understanding and promote more profound learning.

At the *cognitive level*, the most critical weakness lies in the Knowing domain, where South African learners performed substantially below their Singaporean peers (357 vs 624). This indicates fragile mastery of basic facts, operations, and recall skills that form the foundation for higher-order reasoning. Consequently, learners struggle to transition from applying to reasoning, resulting in a cycle where weak early-grade foundations inhibit advanced problem-solving. The comparatively stronger performance in applying suggests that learners can transfer knowledge to routine contexts when concepts are well taught, but limited exposure to non-routine, exploratory activities constrains reasoning development.

The cross-national contrast with Singapore illustrates the value of systemic alignment. Singapore’s curriculum exhibits strong internal coherence, guided by the Concrete–Pictorial–Abstract (CPA) model and a spiral structure that reinforces conceptual progression ( Leong et al., 2015). In contrast, South Africa’s curriculum spreads content thinly across grades, sacrificing depth. Uneven teacher preparation and large class sizes further undermine instructional consistency. Consistent with Curriculum Alignment Theory ( Porter, 2002), effective learning requires harmony between curriculum intent, classroom enactment, and assessment expectations. The misalignment in South Africa is evident: policy frameworks prioritise reasoning and conceptual understanding, yet classroom practice still heavily relies on rote learning and procedural repetition. Addressing this misalignment demands integrated reform that connects curriculum, pedagogy, and professional learning. Teacher development programmes should emphasise diagnostic assessment, conceptual teaching of geometry, and scaffolding of reasoning through open-ended questioning and collaborative tasks. School-level supports such as instructional coaching and communities of practice can facilitate the translation of curriculum goals into classroom realities.

The results present a dual challenge: (1) foundational gaps in number sense, fluency, and spatial reasoning require systematic attention; and (2) learners’ relative strength in application should be leveraged as a pathway toward reasoning and higher-order thinking. Sustainable progress will depend on coherent interventions that coordinate curriculum design, professional development, and classroom innovation. Without such alignment, improvements in application will remain fragmented, limiting advancement toward deeper mathematical reasoning and STEM readiness.

### Differentiation from previous research

Most South African studies using TIMSS data focus on Grade 9, highlighting long-term inequalities and secondary-level deficits. This study’s focus on Grade 5 performance adds novel evidence at the stage when foundational mathematical concepts are consolidated and future trajectories are set. By disaggregating results across content and cognitive domains, it reveals early learning disparities that aggregate reports often obscure. The comparative lens with Singapore enhances interpretive depth by demonstrating how curriculum alignment, pedagogical scaffolding, and teacher preparation in high-performing systems cultivate balanced cognitive development. Thus, this paper contributes grade-specific, comparative evidence that complements rather than duplicates existing analyses, offering practical insights for foundational-phase teacher education and classroom reform.

### Policy and practice implications

The TIMSS 2023 results demonstrate that socioeconomic and systemic inequalities, which directly manifest in classroom practice, entrench South Africa’s mathematics challenges ( Taylor, 2019). Learners in Quintile 1–3 schools, serving the poorest communities, often face overcrowded classrooms, resource scarcity, and underqualified teachers. These conditions constrain formative assessment, differentiated instruction, and sustained skill development. Although pro-poor funding policies provide some redress, resources remain inadequate to counter entrenched disparities. Drawing comparative insights from Singapore while contextualising them for South Africa, this study proposes three interrelated pillars of reform, anchored in teachers’ daily practice.

### Pillar 1: Strengthening foundations through diagnostic teaching

Persistent deficits in the knowing domain indicate that many learners progress without mastering number facts, place value, and basic operations. Policy should prioritise early diagnostic assessments in Grades 1–3 to identify learning gaps before they widen. Schools, particularly in low-SES contexts, could implement structured catch-up programmes modelled on Singapore’s Learning Support Programme, adapted for multilingual and resource-limited settings. Professional development in formative assessment and error analysis will enable teachers to translate diagnostic data into responsive teaching. Dedicated funding for remedial support staff, small-group tutors, and low-cost learning materials would operationalise this pillar at the school level.

### Pillar 2: Enhancing geometry and spatial reasoning through pedagogical innovation

Measurement and geometry remain the weakest domains, highlighting persistent gaps in spatial reasoning. Professional learning programmes should therefore emphasise visual, experiential, and problem-based approaches to geometry. In resource-constrained schools, teachers can employ locally available materials, outdoor activities, and hand-drawn representations to cultivate geometric understanding. Over time, they can be complemented by affordable digital visualisation tools. Embedding spatial reasoning modules in pre-service and in-service teacher training will institutionalise these practices across the system.

### Pillar 3: Leveraging learners’ strength in applying to cultivate reasoning

The relatively stronger The relatively stronger performance in the Applying category provides an opportunity to foster deeper reasoning. Curriculum reform should integrate contextually relevant problem-solving tasks drawn from everyday experiences, such as budgeting or community-based measurement activities, to enhance engagement and conceptual transfer. Teachers should be supported to design inquiry-based lessons that promote explanation, justification, and collaboration. District-level mentoring and professional learning communities can help disseminate and sustain these pedagogical innovations.

### System-level coherence: Linking policy, curriculum, and classroom practice

The effectiveness of these pillars depends on systemic coherence from early childhood education through the intermediate phase. Singapore’s ongoing progress shows that real change happens when policy goals, teacher training, classroom practice, and assessment design all work together. Accordingly, South Africa’s Department of Basic Education must integrate curriculum reform with continuous professional development, accountability structures, and resource provision. Reducing class sizes, ensuring consistent access to learning materials, and strengthening school-based mentoring will translate curriculum goals into meaningful learning. Ultimately, bridging policy and practice requires recognising teachers as the primary agents of change. When professional learning, curriculum design, and resourcing converge around clear pedagogical objectives, early mathematics education can evolve from procedural instruction toward conceptual understanding, advancing equitable foundational learning in line with Sustainable Development Goal 4 (SDG-4): Quality Education.

## Conclusion

This study analysed South African Grade 5 learners’ mathematics achievement in TIMSS 2023, disaggregated by content and cognitive domains and benchmarked against Singapore’s Grade 4 performance. The results confirm earlier research ( Mabena et al., 2021; Taylor, 2021) that South Africa continues to perform well below international benchmarks, with particularly severe weaknesses in measurement and geometry and in the knowing domain. These persistent gaps reflect deep structural inequities within the education system, rooted in socioeconomic disparities, uneven teacher preparation, and limited curriculum coherence. Encouragingly, learners’ relatively stronger performance in the applying domain demonstrates their ability to transfer knowledge to routine problem-solving contexts when they understand foundational concepts well. This strength provides a critical entry point for instructional improvement and curriculum redesign, which emphasises conceptual understanding.

Building on these insights, the study proposes a reform agenda centred on strengthening foundational knowledge through early diagnostic assessment and catch-up programs; enhancing geometry and spatial reasoning through sustained professional development focused on visualisation and conceptual teaching; and leveraging learners’ ability to apply knowledge by embedding authentic, context-based problem-solving tasks that bridge procedural fluency and higher-order reasoning. The coherence of curriculum design, teacher education, classroom practice, and assessment determines the effectiveness of these reforms. Drawing on Curriculum Alignment Theory ( Kulasegaram et al., 2018; Pinar, 2019; Porter, 2002), reform succeeds when the intended, implemented, and achieved curricula reinforce one another. Achieving such coherence requires long-term investment in teacher learning communities, instructional coaching, and equitable support for schools in low-socioeconomic contexts. Combining curriculum refinement with sustained teacher development and adequate resourcing can transform early mathematics instruction from rote learning to conceptual reasoning.

The Department of Basic Education (2018) and Costello et al. (2020) both say that teaching basic skills along with transversal skills like problem-solving, critical thinking, and working together leads to more creative and fair mathematics learning. Taken together, these results suggest that lasting improvement in mathematics achievement will emerge not from isolated interventions but from continuous professional learning and pedagogical innovation that connect national policy with classroom practice and advance Sustainable Development Goal 4 on quality education.

## Limitations and future research

Although this research offers invaluable information about South African Grade 5 mathematics achievement, several limitations warrant acknowledgement. First, the analysis relied on secondary, cross-sectional data from TIMSS 2023, which precludes causal inference. The observed associations among content and cognitive domains should therefore be interpreted as correlational rather than causal. Policymakers often operate under conditions of incomplete information that may lead to suboptimal decisions ( Byman, 2024); hence, the results should inform but not overdetermine policy responses. Second, the domain-specific subscales, such as knowing and measurement and geometry, are based on fewer test items than the overall mathematics scale, introducing potential measurement errors and limiting precision. Although the use of plausible values enhances population-level reliability, it also introduces statistical uncertainty that must be considered when interpreting effect sizes.

Third, the use of secondary data restricts the ability to examine classroom processes, teaching strategies, and learner experiences that influence achievement. Without qualitative or longitudinal evidence, it remains difficult to capture how curriculum intentions are enacted or how learners engage with mathematical tasks in practice. This limitation points to the need for mixed-methods research that integrates large-scale assessment data with classroom observations, teacher interviews, and learner case studies. Fourth, while the TIMSS sampling design ensures national representativeness, it may under-represent learners in marginalised or rural settings where multi-grade teaching, language mismatches, and severe resource shortages persist. Future research should therefore include oversampling or targeted case studies in these contexts to illuminate how systemic inequities shape foundational learning.

Future inquiry should extend this work through design-based and experimental studies that align with the reform framework articulated here. Pilot interventions could evaluate the effectiveness of early diagnostic assessments and structured catch-up programmes in low-SES schools, assessing whether they narrow foundational knowledge gaps. Classroom-based investigations could examine how educators integrate cost-effective geometry and reasoning activities, with or without digital tools, to enhance spatial and cognitive development. Further, experimental and longitudinal designs could examine how embedding authentic, real-life application tasks influences learners’ problem-solving and reasoning abilities. Such research would not only address the limitations of cross-sectional secondary data but also provide empirical evidence to guide curriculum reform, teacher education, and policy innovation aimed at improving mathematics achievement and equity in South Africa.

## Declaration of AI use

The author affirms that no generative artificial intelligence tools (such as ChatGPT or similar models) were used to produce the academic content, analysis, or interpretations presented in this manuscript. QuillBot (premium) was employed solely for grammar and spelling checks. The author personally reviewed and edited the final manuscript and takes full responsibility for its content and conclusions.

## Data Availability

The data that support the results of this article are derived from the publicly accessible Trends in International Mathematics and Science Study (TIMSS) 2023 database, available through the International Association for the Evaluation of Educational Achievement (IEA) at https://timssandpirls.bc.edu. Derived and processed data underlying the statistical analyses, including the variables used to generate the tables, figures, and descriptive statistics presented in this article, are openly available in the Zenodo repository ( Mokgwathi, 2025) at https://doi.org/10.5281/zenodo.17379412, under the Creative Commons Attribution 4.0 International (CC BY 4.0) licence. These files include anonymised values underlying the means, standard deviations, and effect sizes, as well as detailed variable descriptions and methodological notes supporting replication of the comparative analyses between South Africa (Grade 5) and Singapore (Grade 4). No ethical approval or participant consent was required, as all data originate from secondary sources that are publicly available through the IEA TIMSS 2023 repository. Supplementary materials supporting this article are available in the Zenodo repository at https://doi.org/10.5281/zenodo.17379412 ( Mokgwathi, 2025), under the CC BY 4.0 license. These include: TIMSS2023_SGvZA_Derived_Dataset.xlsx (domain-level statistics and effect sizes), TIMSS2023_Variable_Descriptions.xlsx (variable definitions and sources), and TIMSS2023_Supplementary_Notes.pdf (methodological documentation).
