# Closing the Gap in Early Mathematics: Domain and Cognitive Insights from TIMSS 2023 in South Africa and Singapore

**DOI:** 10.12688/f1000research.172015.2

**Published:** 2026-01-28

**Authors:** Mathelela Steyn Mokgwathi

**Affiliations:** 1 Department of Early Childhood Education, University of South Africa, Pretoria, Gauteng, South Africa

**Keywords:** Trends in International Mathematics and Science Study (TIMSS), Mathematics Achievement, Content and Cognitive Domains, South Africa, Singapore

## Abstract

**Background:**

South Africa continues to underperform in primary mathematics, with persistent foundational gaps across content and cognitive domains. This study examines domain-specific patterns of mathematics achievement using data from the Trends in International Mathematics and Science Study (TIMSS) 2023, benchmarking South African Grade 5 learners against Singaporean Grade 4 learners.

**Methods:**

A quantitative secondary analysis was conducted using nationally representative TIMSS 2023 datasets comprising 10,424 South African learners from 285 schools and 6,530 Singaporean learners from 181 schools. In TIMSS 2023, South Africa assessed learners in Grade 5 using the internationally standardised TIMSS Grade 4 mathematics assessment framework, a practice adopted within TIMSS where curriculum exposure aligns more closely with this level. Singapore assessed learners at Grade 4 using the same instruments. Mathematics achievement was analysed across content domains (number, measurement and geometry, and data) and cognitive domains (knowing, applying, and reasoning). Weighted estimates, mean differences, and effect sizes (Cohen’s d) were computed in line with IEA guidelines.

**Results:**

South African learners performed well below the international centre point across all domains. The largest content-level gap relative to Singapore occurred in measurement and geometry, indicating persistent weaknesses in spatial reasoning. At the cognitive level, the most pronounced deficit was in the knowing domain, reflecting fragile mastery of basic facts and procedural fluency. Although performance in applying was comparatively stronger, limitations in foundational knowledge constrained progression to reasoning, particularly in non-routine problem-solving.

**Conclusions:**

Weak foundations, underdeveloped spatial reasoning, and limited opportunities for higher-order thinking characterise early mathematics learning in South Africa. Targeted support for number fluency and geometry instruction, alongside pedagogical approaches that leverage application to scaffold reasoning, is required. Improved alignment between curriculum design, teacher development, and assessment practices is essential for advancing equitable foundational learning outcomes.

## 1. Introduction

Mathematics achievement at the primary school level has profound implications for learners’ future participation in education, work, and society. Basic mathematics skills support higher-order thinking, problem-solving, and lifelong learning, all of which are central to academic success and broader social participation, as consistently demonstrated in international large-scale assessment research ( Mullis et al., 2020; von Davier et al., 2024). TIMSS has become a critical assessment instrument for benchmarking mathematical learner achievement internationally, revealing how national curricula, teaching practices, and educational systems prepare learners for cognitive demands in mathematics. South Africa has participated in TIMSS since 1995 and, despite modest gains over successive cycles, continues to rank among the lowest-performing education systems internationally ( Reddy & Hannan, 2019; Zuze et al., 2018; Mullis et al., 2020). Much of the existing research focuses on Grade 9 data, emphasising long-term learning deficits and systemic inequities ( Mensah & Baidoo-Anu, 2022; Reddy & Hannan, 2019). Far less attention has been given to the primary phase, particularly Grade 5, where learners consolidate fundamental numeracy and begin to transition from concrete to abstract reasoning. TIMSS 2023 results indicate that South African Grade 5 learners achieved an average score of 362 compared to Singapore’s Grade 4 average of 615 (TIMSS, 2023). This 250-point gap, despite Singaporean learners being a grade lower than South African learners, signals deep foundational weaknesses in South Africa’s education system, inclusive of mathematics education. In TIMSS 2023, while the international benchmarking grades are 4 and 8, some education systems assess an adjacent grade using the same internationally calibrated instruments when curriculum alignment indicates that the benchmark framework better matches learners’ opportunity to learn. In this study, South Africa’s Grade 5 cohort is analysed because it was assessed using the TIMSS Grade 4 mathematics assessment instruments and framework, which supports a valid cross-national comparison with Singapore’s Grade 4 results on the same measurement scale ( Mullis et al., 2020; von Davier et al., 2024).

National assessments such as the Annual National Assessments (ANA), which is now discontinued, and systemic evaluation reports have highlighted low achievement levels in mathematics among learners, but they rarely explore *how* learners perform across specific content and cognitive domains. Yet TIMSS distinguishes between three content domains (numbers, measurement and geometry, and data) and three cognitive domains (knowing, applying, and reasoning). Assessing learner achievement through these lenses allows for a diagnostic understanding of learners’ strengths and weaknesses. Research from high-performing education systems such as Singapore demonstrates that consistent curriculum alignment, spiral progression, and scaffolded teaching and learning cultivate a balanced development of knowledge, application, and reasoning skills ( Choy & Dindyal, 2024; Low & Wong, 2021; Morony, 2023; Mullis et al., 2020). Conversely, South African studies point to persistent challenges in geometry, reasoning, and teacher content knowledge ( Maqoqa, 2024; Taylor, 2021), compounded by curriculum overload and large class sizes that limit opportunities for formative assessment and conceptual engagement. Improving mathematics learner achievement therefore requires more than curriculum reform; it depends on strengthening the consistency between curriculum design and development, teacher professional development, and classroom practice. Knowing how learners interact with the content and cognitive demands offers crucial understanding of the areas that require the most instructional support and pedagogical innovation.

This study addresses these gaps by analysing South African Grade 5 learners’ performances at TIMSS 2023 relative to Singaporean Grade 4 learners. It contributes in three key ways. Firstly, it focuses on the under-researched area of upper primary mathematics learning, where learners consolidate foundational numeracy and transition from concrete to more abstract mathematical reasoning. Second, it breaks down performance by content and cognitive areas to find specific patterns of strength and weakness. Third, it links these results to curriculum and pedagogical implications, proposing strategies to strengthen basic knowledge, geometry teaching, and teacher professional development. The study was guided by the following research questions:

### 1.1 Research Questions

1. What are the patterns of performance for South Africa’s Grade 5 learners on the TIMSS 2023 primary mathematics assessment across the content domains (number, measurement and geometry, and data), and how do these patterns compare with Singapore’s Grade 4 learners assessed on the same TIMSS primary mathematics instrument?
2. How do South African learners perform across the TIMSS 2023 cognitive domains (knowing, applying, and reasoning), and what specific strengths and weaknesses are revealed in comparison with Singapore?
3. What curriculum and pedagogical implications can be drawn from the comparative analysis of domain-specific and cognitive performance to inform strategies for improving mathematics achievement in South Africa?

### 1.2 Literature Review: Benchmarking South Africa’s Grade 5 Results on the TIMSS Primary Mathematics Assessment against Singapore’s Grade 4

**Benchmarking with TIMSS 2023**

The Trends in International Mathematics and Science Study (TIMSS) is a global standard for measuring how well primary and secondary school students do in math. Singapore was consistently ranked as the top achiever, with learners assessed at Grade 4 achieving an overall average of 615 points, while South Africa, assessed at Grade 5, scored 362 points. This means that Singaporean learners who are on average a year younger still outperform South African learners by more than 250 points ( von Davier et al., 2024). The magnitude of this achievement gap underscores the need to analyse not just overall scores but also performances across content domains (numbers, measurements, geometry, and data) and cognitive domains (knowing, applying, and reasoning) to understand how curricula and teaching practices shape outcomes.

**Curriculum Alignment and Content Domains**

Singapore’s mathematical curriculum is internationally recognised for its coherence and spiral structure, which involves systematically revisiting concepts at increasing levels of complexity. In TIMSS 2023, Singapore scored 613 in numbers, 619 in measurement and geometry, and 616 in data, while South Africa scored 362, 353, and 362, respectively. Maqoqa (2024) and Tachie (2020) reported that the largest achievement gap is in measurement and geometry (266 points), an area long identified as a “blind spot” in South African classrooms. These gaps suggest that South African learners struggle with reasoning and geometric domains, while Singaporean learners benefit from early exposure to concrete manipulatives, reasoning, and visual models that build conceptual skills and knowledge.

**Cognitive Demands: Knowing, Applying, and Reasoning**

TIMSS distinguishes between three cognitive domains: knowing, applying, and reasoning. Singapore’s Grade 4 learners achieved 624 in knowing, 615 in applying, and 609 in reasoning, whereas South Africa’s Grade 5 learners scored 357, 366, and 363. This result reveals a profound weakness in knowing (–267 points compared to Singapore), which reflects learners’ difficulties with knowing domains. South African learners performed slightly better in the applying domain (366) relative to their average, suggesting that when knowledge is available, learners can engage in routine applications. The reasoning domain, on the other hand, shows a persistent weakness. The result means that South African learners are not being prepared for non-routine, multi-step problem-solving tasks, which is a strong point of the Singaporean system.

### 1.3 Instructional and Structural Factors

Several systemic factors reinforce these disparities. According to Meier and West (2020), South Africa’s classrooms often suffer from overcrowding, with class sizes averaging over 50 learners, which hinders formative feedback and personalised support. Teacher content and pedagogical knowledge remain uneven, particularly in geometry and measurement ( Bhagwonparsadh & Pule, 2024; Taylor, 2021). In contrast, Singapore invests heavily in sustained teacher development, smaller class sizes, and instructional leadership, creating a conducive learning environment where consistent teaching and learning take place. Furthermore, although South Africa’s curriculum aims for comprehensive coverage, it has faced criticism for being “overloaded” and not allowing adequate time for the mastery of fundamental skills ( Milne & Mhlolo, 2021). By contrast, Singapore’s Concrete–Pictorial–Abstract (CPA) approach deliberately scaffolds learning so that conceptual understanding precedes abstraction, enabling positive learner achievement in higher-order reasoning ( Leong et al., 2015; Lutfi & Dasari, 2024).

### 1.4 Curriculum–Cognitive Alignment in International Research

International evidence further illustrates how disparities between curriculum objectives and classroom practices shape academic learner achievement. Yılmaz et al. (2021) found that, while mathematics curricula emphasised reasoning, textbook activities leaned more toward application, creating a mismatch between intended and taught cognitive emphases. Similarly, Bulut and Taşpınar-şener (2023) reported that the application domain is most frequently prioritised in the secondary mathematics curriculum, disregarding the primary mathematics curriculum, whereas the emphasis placed on knowledge and reasoning differs across grade levels. In primary schools, Pertiwi and Wahidin (2020) showed that fourth-grade assessments are dominated by number-related content, with far less attention given to geometry or data activities. These results reflect TIMSS’s framework, where knowing entails factual recall and procedural fluency, applying involves transferring knowledge to structured contexts, and reasoning requires non-routine problem-solving and critical thinking ( Peduk & Ateş, 2019). Importantly, the study also found that content domains exert a more positive effect on mathematics learner achievement than cognitive domains. This body of research provides an explanatory lens for South Africa’s TIMSS 2023 mathematics learner achievement. The relative strengths of South African Grade 5 learners in the application domain, alongside their persistent weaknesses in knowledge and reasoning, reflect the misalignment noted by several studies internationally, where instruction and textbooks emphasise routine application but fail to develop the basic knowledge and reasoning capacity required for learner progression. In contrast, Singapore’s balanced curriculum and pedagogy demonstrate how alignment across content and cognitive domains fosters sustained learner achievement.

### 1.5 TIMSS: A Diagnostic Instrument

TIMSS offers a diagnostic lens to evaluate the effectiveness of the current curriculum and teaching practices, rather than viewing the legacies of apartheid inequalities as the main reason for the ongoing poor achievement in mathematics among learners. The comparison with Singapore shows that South Africa’s curriculum is superficially similar in content but not in cognitive expectations, especially when it comes to knowing and reasoning. This mismatch leaves learners underprepared for both academic progression and broader applications of mathematics in their everyday lives. The fact that Singapore’s Grade 4 learners significantly outperform South Africa’s Grade 5 learners further shows that the gap in mathematics achievement among learners is not simply attributable to learner age or exposure but to differences in curriculum coherence, cognitive scaffolding, and teacher training and development.

The TIMSS 2023 results highlight not just the scale of South Africa’s underperformance but also its domain-specific and cognitive weaknesses. While Singapore demonstrates how curriculum coherence and sustained teacher training and development can support an understanding of teaching and learning across content and cognitive domains, South Africa’s challenges are rooted in weak foundations, gaps in geometry and measurement, and systemic barriers in teacher training, development, and the classroom environment. Addressing these challenges requires targeted curriculum reforms in early-grade numeracy, curriculum streamlining, and teacher professional development, with a particular emphasis on geometry, critical thinking (reasoning), and basic knowledge. Context-sensitive adaptations, rather than comprehensive importation of Singapore’s model, offer a pathway for South Africa to strengthen learner trajectories in mathematics.

### 1.6 Conceptual Framework

This study is guided by the TIMSS conceptual model, which distinguishes between mathematical content domains (numbers, measurements, geometry, and data) and cognitive domains (knowing, applying, and reasoning) ( Mullis et al., 2020). Together, these dimensions provide a diagnostic lens for examining not only what learners are expected to know but also how they engage with mathematical tasks at increasing levels of cognitive demand. In light of South Africa’s historically low mathematics achievement, this framework facilitates the identification of domain-specific deficiencies in foundational knowledge, procedural proficiency, and higher-order reasoning, which are frequently obscured by overall performance averages.

The study employs curriculum alignment theory, which emphasises the coherence between curricular intentions, classroom enactment, and assessment demands, to interpret these patterns ( Porter, 2002). International evidence from high-performing systems such as Singapore illustrates how strong alignment is achieved through a spiral curriculum that revisits mathematical concepts at progressively higher levels of complexity, supported by the Concrete–Pictorial–Abstract (CPA) approach that scaffolds learning from concrete representations to abstract reasoning ( Leong et al., 2015; Lutfi & Dasari, 2024). In contrast, research on South African primary mathematics reveals an overloaded curriculum, limited instructional time for mastery of foundational domains, and persistent weaknesses in geometry and spatial reasoning, compounded by gaps in teacher content knowledge and pedagogical confidence ( Maqoqa, 2024; Taylor, 2021). Situating South Africa’s Grade 5 TIMSS 2023 performance against Singapore’s Grade 4 results therefore provides a comparative curriculum lens for examining how differences in sequencing, pacing, and instructional coherence shape progression from basic knowledge to application and reasoning.

To strengthen the theoretical grounding of the cognitive domains, the study further draws on Kilpatrick, Swafford, and Findell’s framework for mathematical proficiency, which conceptualises mathematical competence as comprising five interrelated strands: conceptual understanding, procedural fluency, strategic competence, adaptive reasoning, and productive disposition ( Kilpatrick et al., 2001). Within this framework, the TIMSS cognitive domains align closely with core strands of mathematical proficiency. The knowing domain corresponds primarily to procedural fluency and factual recall, reflecting learners’ command of basic operations and mathematical facts. The applying domain aligns with strategic competence, as it involves selecting and executing appropriate procedures in familiar problem contexts. The reasoning domain maps onto adaptive reasoning, requiring learners to justify solutions, generalise patterns, and solve non-routine problems. Interpreting TIMSS results through this lens situates learner performance within a coherent model of learning progression, where weaknesses in foundational proficiency constrain advancement toward higher-order reasoning and conceptual integration ( Mullis & Martin, 2017).

The study utilises an integrative conceptual framework that amalgamates three complementary dimensions, building upon these perspectives. First, a comparative lens is employed to examine South Africa and Singapore at macro (system-level policy and curriculum design), meso (school and classroom practices), and micro (learner performance across domains) levels. Second, the TIMSS curriculum model is used to distinguish between intended, implemented, and attained curricula, as well as between content and cognitive domains ( Mullis et al., 2020). Third, curriculum theory dimensions are included, such as alignment, spiral progression, CPA scaffolding ( Lutfi & Dasari, 2024), and Grundy’s (1992) product–process–praxis–context typology. Together, these elements ensure that the framework is both diagnostic, by identifying specific patterns of learner strength and weakness, and explanatory, by linking observed performance to curriculum structure, instructional practices, and pedagogical coherence.

**Figure 1.**
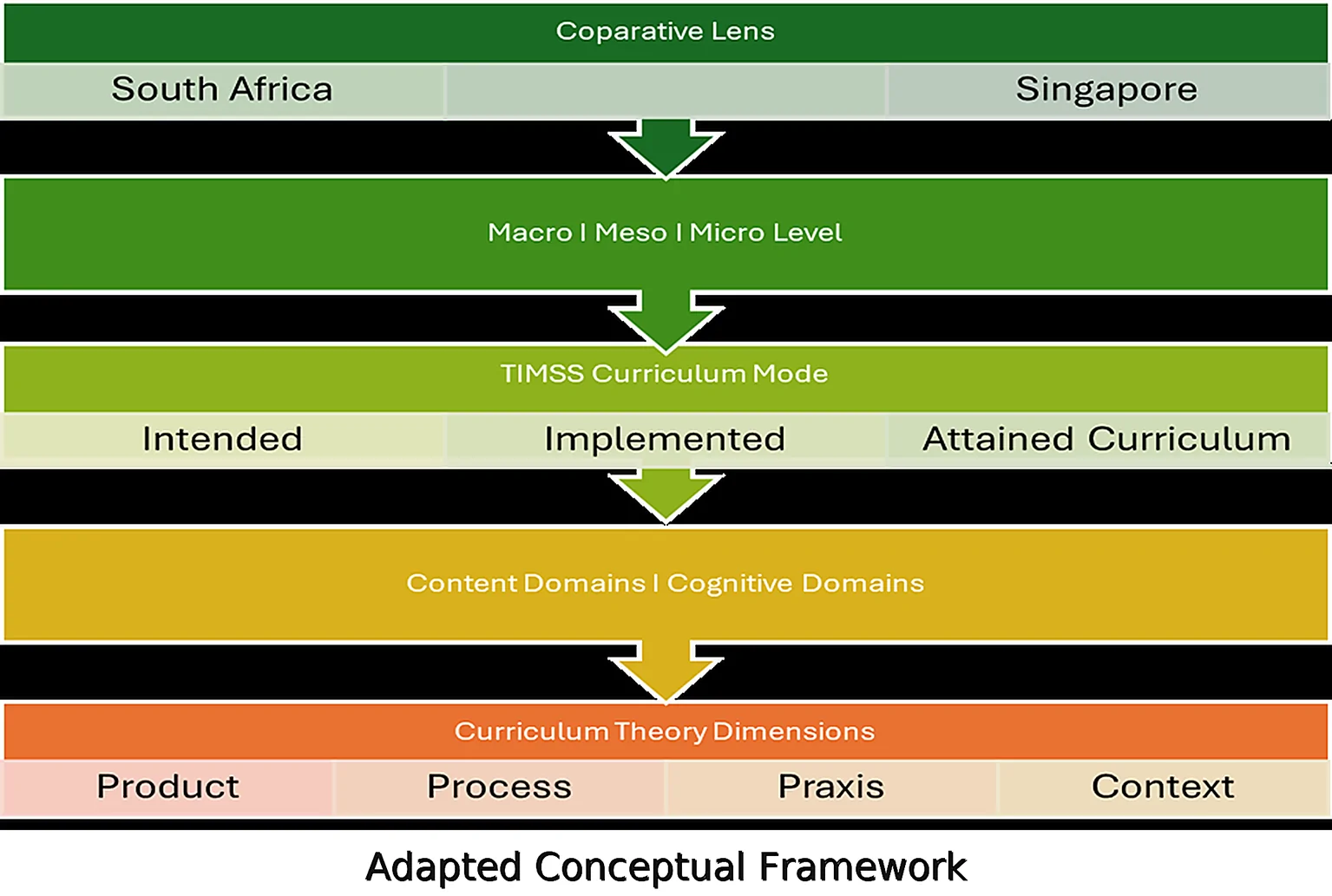
Adapted conceptual framework combining the comparative lens, TIMSS curriculum model, and curriculum theory dimensions guiding the study.

Figure 1 illustrates this integrative conceptual framework, showing how comparative, curricular, and theoretical dimensions interact to guide the analysis.

Guided by this framework, the study’s methodology was designed to operationalise these dimensions through a secondary analysis of the TIMSS 2023 data. The TIMSS curriculum model informed the analysis of content and cognitive domains, the comparative lens enabled benchmarking of South African Grade 5 learners against Singaporean Grade 4 learners, and curriculum theory shaped the interpretation of performance patterns in relation to alignment, learning progression, and pedagogy. The following section therefore outlines the research design, participants, data sources, analytical procedures, and ethical considerations employed in the study.

## 2. Methodology

### 2.1 Research Design

This study employed a quantitative secondary data analysis design, using data from the Trends in International Mathematics and Science Study (TIMSS) 2023. This design is particularly appropriate for investigating the mathematics achievement of South African Grade 5 learners across content and cognitive domains for several reasons. First, TIMSS provides a large, internationally standardised dataset that is both rigorous in design and nationally representative, making it suitable for examining learners’ performance patterns with a high degree of reliability. Second, secondary analysis enables the use of TIMSS’s robust psychometric procedures, including item response theory and plausible values, which strengthen the validity of inferences about learners’ achievement. Third, the TIMSS framework allows for meaningful international benchmarking, making it possible to situate South Africa’s performance in relation to high-performing education systems such as Singapore. Guided by the TIMSS assessment framework, the analysis focused on three content domains (numbers, measurement, geometry, and data) and three cognitive domains (knowing, applying, and reasoning). This two-fold focus not only enabled a diagnostic assessment of learners’ strengths and weaknesses within the South African context but also provided comparative insights to inform curriculum and policy reform globally. Importantly, the South African sample reported here reflects the TIMSS 2023 Grade 5 administration using the Grade 4 mathematics assessment instruments and framework. The analysis therefore benchmarks Grade 5 in South Africa against Grade 4 in Singapore on an equivalent instrument, enabling comparison of performance patterns across TIMSS content and cognitive domains on the same international reporting scale ( Mullis et al., 2020; von Davier et al., 2024).

### 2.2 Participants

The South African TIMSS 2023 primary grade sample comprised 10,424 Grade 5 learners from 285 schools who were assessed using the TIMSS Grade 4 mathematics instruments, while Singapore assessed 6,530 Grade 4 learners from 181 schools using the same Grade 4 mathematics assessment framework ( Department of Basic Education, 2024; von Davier et al., 2024). The International Association for the Evaluation of Educational Achievement (IEA), in collaboration with Statistics Canada, used a two-stage stratified cluster sampling methodology to guarantee nationally representative estimates ( Siegel & Foy, 2024). In the first stage, schools were selected with probabilities proportional to their size, and in the second stage, intact Grade 5 classes were sampled. The stratification variables included the school sector (public or private), the language of instruction, the geographic region, socioeconomic indicators, the degree of urbanisation, and prior academic achievements (Ibid.). This rigorous design ensured that the results accurately reflect the diversity of the South African education system and provide robust population-level estimates of learner performance.

### 2.3 Data Collection and Analysis

Data for this study were drawn from the TIMSS 2023 mathematics assessment and associated contextual background questionnaires administered to participating learners, teachers, and schools. The TIMSS mathematics achievement is reported on an internationally standardised scale with a centre point of 500 and a standard deviation of 100, enabling valid comparisons across countries and education systems. The mathematics assessment comprised 183 items distributed across three content domains, namely number (94 items), measurement and geometry (49 items), and data (40 items), as well as three cognitive domains, namely knowing (58 items), applying (85 items), and reasoning (40 items) ( Reynolds, 2024). Each learner completed one assessment booklet, with achievement estimates derived using item response theory and reported as plausible values. This design supports reliable population-level estimation while minimising respondents’ burden ( von Davier, 2020).

The analysis focused on South Africa’s Grade 5 results, and Singapore’s Grade 4 performance was used as an international benchmark to contextualise domain-specific patterns of mathematics achievement. This comparison is consistent with TIMSS procedures, as both cohorts were assessed using the same Grade 4 mathematics framework and instruments, calibrated on a common international scale. Weighted descriptive statistics were computed in accordance with International Association for the Evaluation of Educational Achievement (IEA) guidelines to account for TIMSS’s two-stage stratified cluster sampling design. Sampling weights were applied to ensure nationally representative estimates and to correct for unequal probabilities of selection and non-response, thereby reducing bias in cross-national comparisons ( Siegel & Foy, 2024). To assess differences in performance across content and cognitive domains, weighted mean scores were compared and tested for statistical significance at the 1% level to account for multiple comparisons. In addition to significance testing, effect sizes (Cohen’s *d*) were calculated to evaluate the practical magnitude of observed differences between domains and between South Africa and Singapore. The combined use of statistical significance and effect size estimation enabled a more substantively meaningful interpretation of performance gaps, beyond reliance on mean differences alone.

Although TIMSS 2023 collects extensive contextual information through learner, teacher, school, and curriculum questionnaires, the present study prioritised a diagnostic comparison of domain-specific achievement patterns. Consequently, contextual variables such as socioeconomic status, language of instruction, school resources, and teacher characteristics were not incorporated into multivariate or multilevel models in the main analysis. Instead, these variables were used interpretively in the discussion to contextualise the observed performance trends. This analytical choice reflects the study’s primary objective, which is to identify where achievement gaps are most pronounced across content and cognitive domains rather than modelling causal relationships. Such an approach is consistent with established practices in large-scale assessment research, where descriptive diagnostics and explanatory modelling are viewed as complementary rather than competitive strategies ( OECD, 2019; Rutkowski & Delandshere, 2016).

To ensure appropriate estimation of statistical uncertainty, all achievement comparisons were based on the full set of TIMSS plausible values, with variance estimation conducted in line with IEA technical guidelines. Weighted means were accompanied by standard errors derived from the complex sampling design, and statistical inference was undertaken using these variance estimates. Effect sizes were reported alongside significance tests to provide an educationally meaningful interpretation of observed differences. Detailed estimates of standard errors and confidence intervals for domain-level comparisons are reported in the supplementary materials.

### 2.4 Ethical Considerations

This study is based on secondary analysis of TIMSS 2023 restricted-use datasets provided by the IEA. The data contain no personal identifiers and were collected under strict international ethical protocols during the original administration. Because this research involved secondary analysis of anonymised data, no institutional ethics approval was required. There was no formal request to use the dataset from the IEA since the data is available in the public domain, and all analyses adhered to its guidelines for responsible data use.

## 3. Results

This section presents the empirical results addressing Research Questions 1 and 2. Research Question 1 examines patterns of performance across the TIMSS mathematics content domains, while Research Question 2 focuses on performance across the cognitive domains of knowing, applying, and reasoning. The Discussion section addresses Research Question 3, which pertains to curriculum and pedagogical implications.

### 3.1 Content Domain Achievement

In response to Research Question 1, which examines patterns of performance across the TIMSS mathematics content domains, the analysis reveals that South African Grade 5 learners perform substantially below Singaporean Grade 4 learners in all three domains. The largest performance gap is observed in Measurement and Geometry, indicating persistent weaknesses in spatial reasoning and conceptual understanding. These patterns suggest that content-related learning gaps are systematic rather than isolated to specific mathematical topics. Table 1 reports the weighted mean scores and effect sizes for South African Grade 5 learners and Singaporean Grade 4 learners across the three TIMSS mathematics content domains.

**Table 1.** Mean Scale Scores and Effect Sizes in Mathematics Content Domains, TIMSS 2023

Content domain	Singapore (Grade 4)	South Africa (Grade 5)	Gap	Cohen’s *d*
Numbers	613	362	251	2.51
Measurement & Geometry	619	353	266	2.66
Data	616	362	254	2.54

*Note.* All estimates are weighted. Standard errors and confidence intervals were computed using TIMSS plausible values and replicate weights and are reported in the supplementary materials.

All three domains reveal large effect sizes ( *d* > 2.5), signifying profound disparities. The most pronounced gap lies in Measurement and Geometry ( *d* = 2.66), confirming that South African learners face persistent difficulties in spatial reasoning and geometric concepts.

### 3.2 Cognitive Domain Achievement

Addressing Research Question 2, which focuses on learner performance across the TIMSS cognitive domains, the results indicate that South African learners demonstrate markedly weaker performance than their Singaporean peers in knowing, applying, and reasoning. The most pronounced disparity occurs in the knowing domain, reflecting fragile foundations in factual knowledge and procedural fluency that constrain progression toward higher-order reasoning.

**Table 2.** Mean Scale Scores and Effect Sizes in Mathematics Cognitive Domains, TIMSS 2023.

Cognitive domain	Singapore (Grade 4)	South Africa (Grade 5)	Gap	Cohen’s *d*
Knowing	624	357	267	2.67
Applying	615	366	249	2.49
Reasoning	609	363	246	2.46

*Note.* All estimates are weighted. Standard errors and confidence intervals were computed using TIMSS plausible values and replicate weights and are reported in the supplementary materials.

The knowing domain shows the largest gap ( *d* = 2.67), underscoring fragile foundations in factual knowledge and procedural fluency. South African learners’ relatively positive achievement in applying (366) suggests that when basic knowledge is accessible, learners can engage with routine procedures. However, the consistent deficits across domains indicate that foundational gaps constrain progression to reasoning tasks, where Singaporean learners excel.

### 3.3 Item-Level Illustrations

To further illustrate the patterns identified in response to Research Questions 1 and 2, selected released TIMSS items are used to demonstrate how differences in content and cognitive demands manifest at the item level. To illustrate the cognitive demands underlying these results, examples from released TIMSS items are useful. In the knowing domain (numbers), more than 90% of Singaporean learners were able to recall multiplication facts correctly, while fewer than 40% of South African learners were able to do the same. This indicates that there are big gaps in basic fact fluency. In the applying domain (data), routine tasks such as interpreting a simple bar chart were accessible to many South African learners, suggesting some competence with structured and familiar problems. However, in the reasoning domain (geometry), where items demanded multi-step reasoning with angles, most Singaporean learners responded correctly compared to fewer than 20% of South African learners, reflecting limited exposure to non-routine problem-solving beyond procedural recall. Collectively, these examples highlight how curriculum exposure and classroom instructional practices shape learners’ preparedness to engage with domain-specific cognitive demands.

### 3.4 Visualising the Gaps

**Figure 2.**
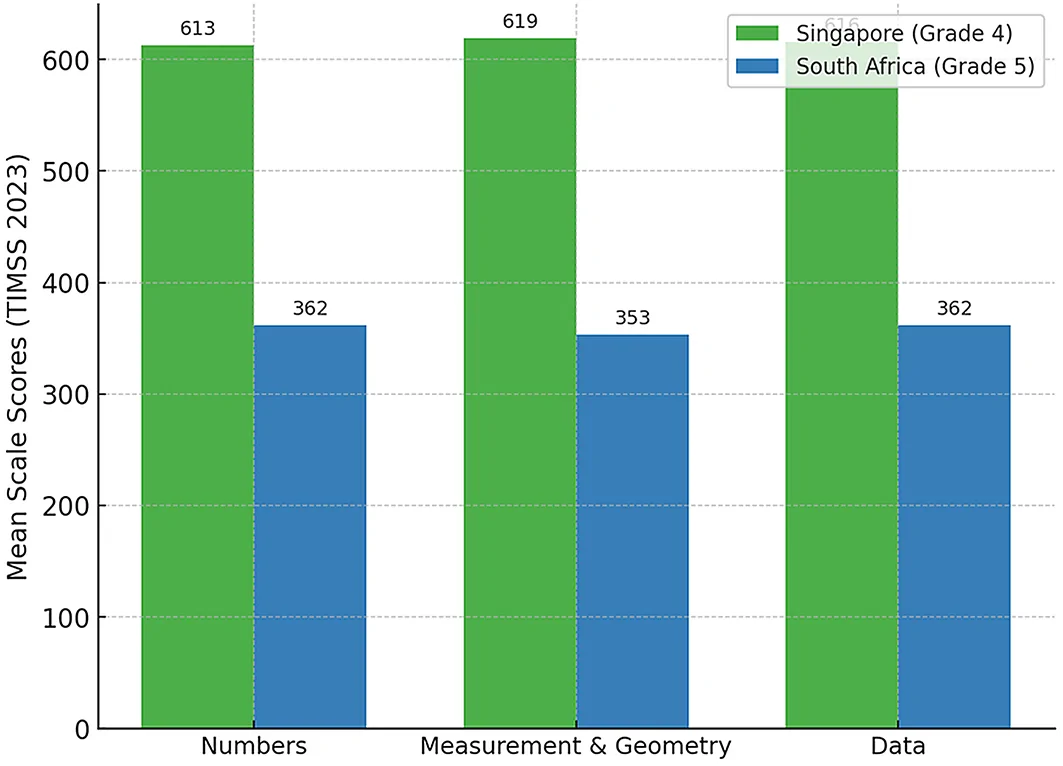
Comparative performance of South African Grade 5 and Singaporean Grade 4 learners across TIMSS content domains (Numbers, Measurement & Geometry, and Data).

**Figure 3.**
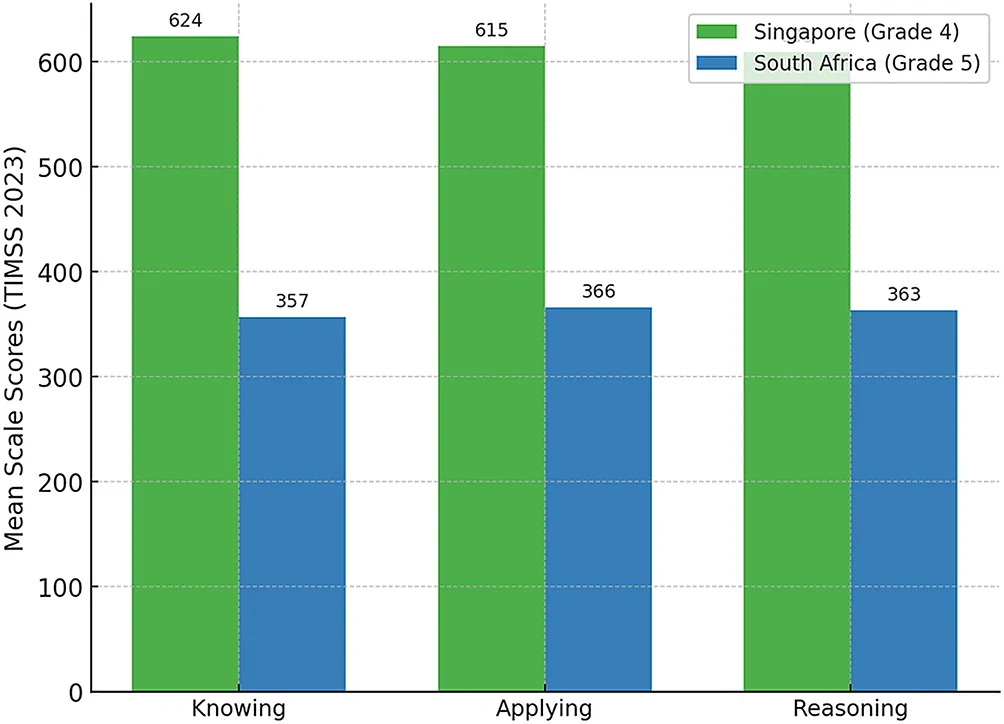
Comparative performance of South African Grade 5 and Singaporean Grade 4 learners across TIMSS cognitive domains (Knowing, Applying, and Reasoning).

Figure 2 illustrates the comparative performance of South African Grade 5 and Singaporean Grade 4 learners across the three TIMSS content domains. The visualisation confirms the consistently lower achievement of South African learners, with the largest gap observed in measurement and geometry. This finding supports the tabulated results and highlights the ongoing challenges that South African learners encounter in spatial reasoning and geometric concepts. To visually reinforce the domain-specific patterns identified in response to Research Questions 1 and 2, Figures 2 and 3 present comparative performance profiles across content and cognitive domains for South African Grade 5 and Singaporean Grade 4 learners. By presenting the disparities graphically, Figure 2 highlights the structural nature of these weaknesses and the extent to which curriculum design and instructional practices shape domain-specific performance.

Figure 3 presents the comparative performance of South African Grade 5 and Singaporean Grade 4 learners across the TIMSS cognitive domains. The figure makes clear that the widest disparity lies in the knowing domain, where South African learners demonstrate severe deficits in foundational knowledge and procedural fluency. While relative performance in applying appears slightly positive, it remains far below international benchmarks, limiting progress to more complex reasoning. These results show how gaps in basic knowledge can lead to fewer chances for higher-order thinking. On the other hand, Singapore’s curriculum scaffolding helps all three cognitive domains grow in a balanced way.

### 3.5 Summary of Results

Synthesising the results addressing Research Questions 1 and 2, the results highlight three critical insights into South African learners’ mathematics achievement. First, geometry and spatial reasoning continue to represent the weakest domain, pointing to enduring structural gaps in both curriculum design and teacher preparation. Second, the severity of deficits in foundational knowledge, reflected in the knowing domain, restricts learners from progressing toward higher-order reasoning tasks. Third, although learners demonstrate relatively positive learner achievement in the applying domain, this potential remains constrained by the absence of solid basic skills and limited opportunities for reasoning skills, which prevents the development of sustained mathematical learner achievement. Collectively, these results suggest that South Africa’s performance challenges are not incidental but rather systematic, domain-specific, and deeply embedded in pedagogical practices. In contrast, the comparison with Singapore emphasises the value of curriculum coherence and deliberate cognitive scaffolding for supporting learners’ steady progress across domains.

## 4. Discussion

This section addresses Research Question 3 by interpreting domain-specific and cognitive performance patterns in relation to curriculum alignment, pedagogy, and teacher professional development and outlining implications for improving mathematics achievement in South Africa. The TIMSS 2023 results reaffirm that South African Grade 5 learners perform substantially below the international mathematics benchmark, achieving a mean score of 362, compared with the TIMSS centre point of 500. While this overall result signals persistent systemic challenges, disaggregating achievement across content and cognitive domains provides a more diagnostic understanding of *where* learning gaps emerge and *how* they constrain curriculum implementation, pedagogy, and teacher preparation.

### 4.1 Content-Domain Patterns and Foundational Gaps

At the content level, learner performance in Number and Data indicates partial competence in basic computation and routine interpretation tasks. However, performance in measurement and geometry is markedly weaker, pointing to persistent challenges in developing spatial reasoning and conceptual understanding. This pattern aligns with national and international evidence identifying geometry as a longstanding area of difficulty in South African primary classrooms, often linked to limited pedagogical content knowledge, curriculum overload, and insufficient use of visual and manipulative-based instructional strategies ( Maqoqa, 2024; Taylor, 2021). Importantly, these results do not suggest an absence of instructional attention to geometry but rather a misalignment between curriculum expectations and classroom enactment. Prior studies have shown that geometry instruction in South Africa frequently emphasises procedural routines and definitions with limited opportunities for learners to explore spatial relationships, visualisation, and reasoning ( Taylor, 2021). Strengthening geometry learning therefore requires pedagogical approaches that prioritise visualisation, contextualisation, and progressive abstraction while using both low-cost physical materials and, where feasible, digital tools to support conceptual development.

### 4.2 Cognitive-Domain Performance and Learning Progression

Analysis of the cognitive domains reveals that the most pronounced weakness lies in the knowing domain, where South African learners scored substantially lower than their Singaporean counterparts (357 versus 624). This gap reflects fragile mastery of basic facts, operations, and recall skills that underpin later mathematical learning. From a learning progression standpoint, deficiencies at this fundamental level limit learners’ capacity to meaningfully engage with higher-order cognitive demands, especially in reasoning and non-routine problem- solving. Although performance in the applying domain is comparatively stronger than in knowing, this relative strength should be interpreted cautiously. The results suggest that learners are able to apply familiar procedures in structured contexts when concepts are explicitly taught, but they struggle to extend this knowledge to unfamiliar or exploratory tasks that require justification and generalisation. This pattern is consistent with research showing that application without deep conceptual grounding does not reliably translate into sustained reasoning ability ( Mullis & Martin, 2017). Consequently, the observed performance profile reflects a disrupted learning trajectory in which limited foundational fluency restricts progression toward adaptive reasoning.

### 4.3 Curriculum Alignment and Cross-National Insights

The comparison with Singapore highlights the role of systemic curriculum alignment in supporting coherent learning progression. Singapore’s curriculum is characterised by a tightly sequenced spiral structure and the use of the Concrete–Pictorial–Abstract (CPA) approach, which systematically scaffolds learners from concrete representations to abstract reasoning ( Leong et al., 2015). This coherence supports sustained development across cognitive domains and reduces fragmentation between knowledge, application, and reasoning. In contrast, South Africa’s curriculum spreads content across grades with limited instructional time for consolidation, particularly in foundational domains. As noted in previous research, this breadth-over-depth approach can undermine mastery and exacerbate cumulative learning gaps ( Maqoqa, 2024; Taylor, 2021). Consistent with Curriculum Alignment Theory ( Porter, 2002), effective learning depends on alignment between curriculum intent, classroom practice, and assessment demands. While South African policy documents emphasise reasoning and conceptual understanding, classroom practice often remains dominated by procedural repetition, reflecting constraints related to teacher preparation, class sizes, and resource availability.

### 4.4 Interpreting Singapore as a Benchmark

While Singapore’s performance provides a valuable benchmark for examining curriculum coherence and learning progression, it is essential to recognise the contextual conditions under which it is achieved. Singapore’s education system operates within a highly structured and competitive environment, characterised by strong central curriculum control, selective teacher recruitment, intensive professional preparation, and high societal expectations regarding academic achievement ( Ng, 2017). These features are accompanied by early differentiation and sustained academic pressure, which, although contributing to high achievement, may also generate stress and equity concerns that are not fully captured in large-scale assessment data ( Tan, 2018). The value of Singapore as a comparator resides not in direct policy transplantation, but in the identification of transferable principles, including curriculum coherence, focused content progression, and pedagogical scaffolding ( Deng, 2013). For South Africa, such an approach implies selective and context-sensitive adaptation rather than wholesale adoption, taking into account systemic capacity, linguistic diversity, and resource constraints ( Spaull & Kotze, 2015).

### 4.5 Implications for Policy and Practice

Taken together, the results point to a dual challenge. First, persistent weaknesses in number sense, procedural fluency, and spatial reasoning require systematic and early intervention. Second, learners’ relative strength in application offers a potential entry point for developing higher-order reasoning, provided that foundational knowledge is strengthened and instructional practices are redesigned to promote conceptual transfer. Addressing these challenges requires coordinated action across curriculum design, teacher professional development, and classroom practice. Reducing class sizes and increasing specialist support are desirable recommendations, but they may face short-term financial or political constraints. Targeted diagnostic assessment, structured small-group instruction, and school-based professional learning communities are examples of incremental strategies that provide more immediately feasible pathways for improvement.

### 4.6 Pillars for Strengthening Foundational Mathematics

**Pillar 1: Strengthening Foundations through Diagnostic Teaching**

The pronounced deficits in the knowing domain indicate that many learners progress without secure mastery of basic mathematical foundations. Early diagnostic assessment in the foundation and intermediate phases can help identify learning gaps before they compound. Adapted support programmes, combined with teacher development in formative assessment and error analysis, can translate diagnostic information into responsive classroom practices.

**Pillar 2: Enhancing Geometry and Spatial Reasoning**

Given the consistently poor performance in measurement and geometry, professional development should prioritise visual, experiential, and problem-based approaches to spatial reasoning. In resource-constrained contexts, locally available materials and outdoor measurement activities can provide effective entry points, supplemented over time by affordable digital visualisation tools.

**Pillar 3: Leveraging Application to Cultivate Reasoning**

The comparatively stronger performance in applying suggests an opportunity to scaffold reasoning through contextually relevant problem-solving tasks. Supporting teachers as they design inquiry-based lessons that emphasise explanation, justification, and collaborative reasoning can help bridge the gap between application and higher-order thinking.

### 4.7 System-Level Coherence

The effectiveness of these pillars depends on systemic coherence from the foundation phase through the intermediate phase. Singapore’s experience demonstrates that sustained improvement occurs when curriculum, assessment, teacher preparation, and professional support operate in alignment. For South Africa, strengthening coherence requires integrating curriculum reform with continuous professional development, school-based mentoring, and realistic accountability mechanisms. Identifying teachers as primary agents of change is central to this process. When curriculum goals, pedagogical support, and resourcing converge, foundational mathematics education can move beyond procedural instruction toward deeper conceptual understanding, supporting more equitable learning trajectories in line with Sustainable Development Goal 4.

## 5. Conclusion

This study examined South African Grade 5 learners’ mathematics achievement in TIMSS 2023, disaggregated by content and cognitive domains and benchmarked against Singapore’s Grade 4 performance. Consistent with earlier research ( Mabena et al., 2021; Taylor, 2021), the results confirm that South Africa continues to perform substantially below international benchmarks, with pronounced weaknesses in measurement, geometry, and the knowing cognitive domain. These patterns point to enduring challenges in foundational knowledge, spatial reasoning, and curriculum coherence that constrain learners’ progression toward higher-order mathematical thinking. Importantly, the results indicate that South African learners demonstrate comparatively stronger performance in the Applying domain than in Knowing or Reasoning, suggesting an ability to use familiar procedures in routine contexts when concepts are adequately taught. This relative strength does not offset foundational deficits; it provides a potential entry point for instructional improvement by leveraging application-oriented tasks to scaffold progression toward reasoning and conceptual understanding. Interpreted through the lenses of the TIMSS framework, mathematical proficiency theory, and curriculum alignment theory, the results highlight how weaknesses in early mastery of basic skills limit advancement along learning progressions.

Building on these insights, the study advances a reform agenda centred on three interrelated priorities: strengthening foundational knowledge through early diagnostic assessment and targeted catch-up support, enhancing geometry and spatial reasoning through sustained professional development focused on visualisation and conceptual teaching, and leveraging learners’ capacity for application by embedding authentic, context-based problem-solving tasks that bridge procedural fluency and higher-order reasoning. These reforms will only work if the curriculum design, teacher training, classroom practice, and assessment systems all work together. Drawing on Curriculum Alignment Theory ( Porter, 2002), meaningful improvement is most likely when the intended, implemented, and attained curricula reinforce one another. Ultimately, the results suggest that sustainable gains in mathematics achievement will not result from isolated interventions but from coordinated and context-sensitive reforms that strengthen teaching practice, support professional learning, and address systemic constraints. By aligning policy priorities with classroom realities, South Africa can move foundational mathematics instruction beyond procedural compliance toward deeper conceptual engagement, advancing equity and contributing to the achievement of Sustainable Development Goal 4 on quality education.

## 6. Limitations and Future Research

While this study offers significant insights into Grade 5 mathematics achievement in South Africa, it is important to acknowledge several limitations. First, the analysis relied on secondary, cross-sectional data from TIMSS 2023, which precludes causal inference. Consequently, the associations identified between content and cognitive domains should be regarded as descriptive rather than causal relationships. While such evidence is valuable for diagnostic and policy-relevant comparison, it cannot on its own establish mechanisms of effect.

Second, domain-specific subscales, particularly those for knowing and measurement and geometry, are based on fewer test items than the overall mathematics scale, which may reduce measurement precision. Although the use of plausible values enhances the reliability of population-level estimates, it also introduces statistical uncertainty that should be considered when interpreting effect sizes and domain-level differences.

Third, the use of large-scale assessment data limits direct examination of classroom processes, instructional strategies, and learner experiences that shape achievement. Without qualitative or longitudinal evidence, it is not possible to observe how curriculum intentions are enacted in practice or how learners engage with mathematical tasks over time. Future research would benefit from mixed-methods designs that integrate TIMSS data with classroom observations, teacher interviews, and learner case studies to deepen explanatory insight.

Finally, although TIMSS sampling ensures national representativeness, it may under-represent learners in marginalised or remote contexts characterised by multigrade teaching, language-of-instruction challenges, and severe resource constraints. Targeted studies and oversampling in such settings could provide a more nuanced perspective on how structural inequalities shape foundational learning trajectories.

Future research should therefore extend this work through longitudinal, experimental, and design-based studies aligned with the reform framework proposed here. Such studies could evaluate the impact of early diagnostic assessment, structured catch-up programmes, and geometry-focused pedagogical interventions in low-socioeconomic contexts. By generating complementary empirical evidence, this research agenda can inform curriculum reform, teacher education, and policy innovation aimed at improving mathematics achievement and equity in South Africa.

## Author Contributions

The author, Mathelela Steyn Mokgwathi, is responsible for the conceptualisation, data curation, formal analysis, investigation, methodology, project administration, resources, software, supervision, validation, visualisation, and writing of the original draft, as well as the writing, review, and editing processes.

## Declaration of AI Use

The author affirms that no generative artificial intelligence tools (such as ChatGPT or similar models) were used to produce the academic content, analysis, or interpretations presented in this manuscript. QuillBot (premium) was employed solely for grammar and spelling checks. The author personally reviewed and edited the final manuscript and takes full responsibility for its content and conclusions.

## Data Availability

The data that support the results of this article are derived from the publicly accessible Trends in International Mathematics and Science Study (TIMSS) 2023 database, available through the International Association for the Evaluation of Educational Achievement (IEA) at https://timssandpirls.bc.edu. Derived and processed data underlying the statistical analyses, including the variables used to generate the tables, figures, and descriptive statistics presented in this article, are openly available in the Zenodo repository ( Mokgwathi, 2025) at https://doi.org/10.5281/zenodo.17379412, under the Creative Commons Attribution 4.0 International (CC BY 4.0) licence. These files include anonymised values underlying the means, standard deviations, and effect sizes, as well as detailed variable descriptions and methodological notes supporting replication of the comparative analyses between South Africa (Grade 5) and Singapore (Grade 4). No ethical approval or participant consent was required, as all data originate from secondary sources that are publicly available through the IEA TIMSS 2023 repository. Supplementary materials supporting this article are available in the Zenodo repository at https://doi.org/10.5281/zenodo.17379412 ( Mokgwathi, 2025), under the CC BY 4.0 license. These include: TIMSS2023_SGvZA_Derived_Dataset.xlsx (domain-level statistics and effect sizes), TIMSS2023_Variable_Descriptions.xlsx (variable definitions and sources), and TIMSS2023_Supplementary_Notes.pdf (methodological documentation).
