# Closing the Gap in Early Mathematics: Domain and Cognitive Insights from TIMSS 2023 in South Africa and Singapore

**DOI:** 10.12688/f1000research.172015.3

**Published:** 2026-04-03

**Authors:** Mathelela Steyn Mokgwathi

**Affiliations:** 1 Department of Early Childhood Education, University of South Africa, Pretoria, Gauteng, South Africa

**Keywords:** Trends in International Mathematics and Science Study (TIMSS), Mathematics Achievement, Content and Cognitive Domains, South Africa, Singapore

## Abstract

**Background:**

South Africa continues to underperform in primary mathematics, yet overall mean comparisons obscure where achievement gaps are most concentrated. This study examines domain-specific patterns of mathematics achievement using data from the Trends in International Mathematics and Science Study (TIMSS) 2023, benchmarking South African Grade 5 learners against Singaporean Grade 4 learners assessed on the same TIMSS Grade 4 mathematics framework.

**Methods:**

A quantitative secondary analysis was conducted using nationally representative TIMSS 2023 datasets comprising 10,424 South African learners from 285 schools and 6,530 Singaporean learners from 181 schools. South Africa assessed learners in Grade 5 using the TIMSS Grade 4 mathematics instruments, consistent with TIMSS procedures where curriculum exposure aligns more closely with the benchmark framework. Mathematics achievement was analysed across content domains (number, measurement and geometry, data) and cognitive domains (knowing, applying, reasoning). Weighted estimates, replicate-based variance estimation, and effect sizes (Cohen's d) were computed in accordance with IEA technical guidelines. The analysis is descriptive and comparative rather than causal.

**Results:**

South African learners performed substantially below the international centre point across all domains. The largest content-domain gap relative to Singapore was observed in Measurement and Geometry, indicating pronounced weaknesses in spatial reasoning. The largest cognitive-domain gap occurred in Knowing, reflecting fragile foundational fluency. Although applying was relatively stronger within the South African profile, it remained substantially below benchmark levels. Achievement gaps were therefore concentrated in foundational and spatial domains rather than uniformly distributed.

**Conclusions:**

Concentrated achievement gaps in foundational knowledge and geometry characterise the underperformance of primary mathematics in South Africa. Strengthening procedural fluency, prioritising spatial reasoning, and improving curriculum alignment between intended, implemented, and assessed domains are highlighted as key areas for focused attention. The findings provide a structured diagnostic profile that may inform targeted, domain-specific curriculum and pedagogical considerations aimed at improving equitable foundational learning outcomes.

## 1. Introduction

Mathematics achievement at the primary school level has profound implications for learners’ future participation in education, work, and society. Basic mathematics skills support higher-order thinking, problem-solving, and lifelong learning, all of which are central to academic success and broader social participation, as consistently demonstrated in international large-scale assessment research ( Mullis et al., 2020; von Davier et al., 2024). The Trends in International Mathematics and Science Study (TIMSS) has become a key tool for comparing math achievement around the world. It shows how different countries' curricula, teaching methods, and education systems get students ready for the mental challenges of math. South Africa has participated in TIMSS since 1995 and, despite modest gains over successive cycles, continues to rank among the lowest-performing education systems internationally ( Mullis et al., 2020; Reddy & Hannan, 2019; Zuze et al., 2018).

Much of the existing research focuses on Grade 9 data, emphasising long-term learning deficits and systemic inequities ( Mensah & Baidoo-Anu, 2022; Reddy & Hannan, 2019). Far less attention has been given to the primary phase, particularly Grade 5, where learners consolidate foundational numeracy and begin transitioning from concrete to abstract reasoning. Empirical studies focusing specifically on Grade 5 domain-level analysis remain limited, particularly within the South African context. The TIMSS 2023 results show that South African Grade 5 students got an average score of 362, while Singapore's Grade 4 students got an average score of 615 (TIMSS, 2023). This 250-point difference, despite Singaporean learners being a grade lower, signals substantial foundational weaknesses in South Africa’s mathematics education system. More specifically, this disparity reflects structured achievement gaps across both mathematical content domains and cognitive domains, rather than a uniform decline across all aspects of mathematics learning.

Preliminary TIMSS 2023 results indicate that these gaps are particularly concentrated in measurement and geometry, as well as in the knowing cognitive domain, suggesting fragile foundations in spatial reasoning and procedural fluency, which are critical for overall mathematical competence and problem-solving skills. Within TIMSS 2023, while the international benchmarking grades are 4 and 8, some education systems assess an adjacent grade using the same internationally calibrated instruments when curriculum alignment indicates that the benchmark framework better reflects learners’ opportunity to learn. In this study, South Africa’s Grade 5 cohort is analysed because it was assessed using the TIMSS Grade 4 mathematics instrument and framework, enabling benchmarking with Singapore’s Grade 4 results on a common measurement scale ( Mullis et al., 2020; von Davier et al., 2024). The comparison therefore does not involve different curricular standards, but rather two cohorts assessed on the same TIMSS Grade 4 framework and reporting scale, ensuring measurement equivalence.

National assessments such as the Annual National Assessments (ANA), now discontinued, and systemic evaluation reports have consistently highlighted low levels of mathematics achievement among learners, yet they rarely examine how performance is distributed across specific content and cognitive domains. TIMSS distinguishes between three content domains (numbers, measurement and geometry, and data) and three cognitive domains (knowing, applying, and reasoning), enabling a more diagnostic analysis of learner performance. In this study, the term “achievement gap” refers to measurable differences in scale scores relative to the TIMSS international centre point and a high-performing benchmark system assessed on the same framework. The focus is therefore diagnostic and comparative rather than causal. Research from high-performing systems such as Singapore demonstrates that consistent curriculum alignment, spiral progression, and scaffolded teaching support balanced development of knowledge, application, and reasoning ( Choy & Dindyal, 2024; Low & Wong, 2021; Morony, 2023; Mullis et al., 2020), while South African studies point to persistent challenges in geometry, reasoning, and teacher content knowledge ( Maqoqa, 2024; Taylor, 2021).

Compounded by curriculum overload and large class sizes, these challenges limit opportunities for formative assessment and conceptual engagement. Improving mathematics achievement therefore requires more than curriculum reform; it depends on strengthening coherence between curriculum design, teacher professional development, and classroom practice. Understanding how learners engage with content and cognitive demands is essential for identifying areas requiring targeted instructional support and pedagogical innovation.

This study therefore examines South African Grade 5 learners’ mathematics achievement in TIMSS 2023, disaggregated across content and cognitive domains and benchmarked against Singapore’s Grade 4 performance on the same TIMSS Grade 4 framework. By identifying where achievement gaps are most pronounced, the study provides a domain-specific diagnostic profile to inform curriculum alignment and pedagogical reform. It contributes in three key ways: first, by focusing on the under-researched area of upper primary mathematics learning; second, by disaggregating performance across content and cognitive domains to identify patterns of strength and weakness; and third, by linking these patterns to curriculum and pedagogical implications aimed at strengthening foundational knowledge, geometry instruction, and teacher professional development.

### 1.1 Research Questions

1. What is the achievement profile of South Africa’s Grade 5 learners across the TIMSS 2023 Grade 4 mathematics content domains (number, measurement and geometry, and data)?
2. What is the achievement profile of South Africa’s Grade 5 learners across the TIMSS 2023 Grade 4 mathematics cognitive domains (knowing, applying, and reasoning)?
3. When benchmarked against Singapore’s Grade 4 learners on the TIMSS Grade 4 framework, where are the largest achievement gaps across content and cognitive domains, and what are their implications for curriculum and pedagogy?

### 1.2 Literature Review: Benchmarking South Africa’s Grade 5 Results on the TIMSS Primary Mathematics Assessment against Singapore’s Grade 4

**Benchmarking with TIMSS 2023**

The Trends in International Mathematics and Science Study (TIMSS) is a global standard for measuring how well primary and secondary school students do in math. Singapore was consistently ranked as the top achiever, with learners assessed at Grade 4 achieving an overall average of 615 points, while South Africa, assessed at Grade 5, scored 362 points. This means that Singaporean learners who are on average a year younger still outperform South African learners by more than 250 points ( von Davier et al., 2024). The magnitude of this achievement gap underscores the need to analyse not just overall scores but also performances across content domains (numbers, measurements, geometry, and data) and cognitive domains (knowing, applying, and reasoning) to understand how curricula and teaching practices shape outcomes.

**Curriculum Alignment and Content Domains**

Singapore’s mathematical curriculum is internationally recognised for its coherence and spiral structure, which involves systematically revisiting concepts at increasing levels of complexity. In TIMSS 2023, Singapore scored 613 in numbers, 619 in measurement and geometry, and 616 in data, while South Africa scored 362, 353, and 362, respectively. Maqoqa (2024) and Tachie (2020) reported that the largest achievement gap is in measurement and geometry (266 points), an area long identified as a “blind spot” in South African classrooms. These gaps suggest that South African learners struggle with reasoning and geometric domains, while Singaporean learners benefit from early exposure to concrete manipulatives, reasoning, and visual models that build conceptual skills and knowledge.

**Cognitive Demands: Knowing, Applying, and Reasoning**

TIMSS distinguishes between three cognitive domains: knowing, applying, and reasoning. Singapore’s Grade 4 learners achieved 624 in knowing, 615 in applying, and 609 in reasoning, whereas South Africa’s Grade 5 learners scored 357, 366, and 363. This result reveals a profound weakness in knowing (–267 points compared to Singapore), which reflects learners’ difficulties with knowing domains. South African learners performed slightly better in the applying domain (366) relative to their average, suggesting that when knowledge is available, learners can engage in routine applications. The reasoning domain, on the other hand, shows a persistent weakness. The result means that South African learners are not being prepared for non-routine, multi-step problem-solving tasks, which is a strong point of the Singaporean system.

### 1.3 Instructional and Structural Factors

Several systemic factors reinforce these disparities. According to Meier and West (2020), South Africa’s classrooms often suffer from overcrowding, with class sizes averaging over 50 learners, which hinders formative feedback and personalised support. Teacher content and pedagogical knowledge remain uneven, particularly in geometry and measurement ( Bhagwonparsadh & Pule, 2024; Taylor, 2021). In contrast, Singapore invests heavily in sustained teacher development, smaller class sizes, and instructional leadership, creating a conducive learning environment where consistent teaching and learning take place. Furthermore, although South Africa’s curriculum aims for comprehensive coverage, it has faced criticism for being “overloaded” and not allowing adequate time for the mastery of fundamental skills ( Milne & Mhlolo, 2021). By contrast, Singapore’s Concrete–Pictorial–Abstract (CPA) approach deliberately scaffolds learning so that conceptual understanding precedes abstraction, enabling positive learner achievement in higher-order reasoning ( Leong et al., 2015; Lutfi & Dasari, 2024).

### 1.4 Curriculum–Cognitive Alignment in International Research

International evidence further illustrates how disparities between curriculum objectives and classroom practices shape academic learner achievement. Yılmaz et al. (2021) found that, while mathematics curricula emphasised reasoning, textbook activities leaned more toward application, creating a mismatch between intended and taught cognitive emphases. Similarly, Bulut and Taşpınar-şener (2023) reported that the application domain is most frequently prioritised in the secondary mathematics curriculum, disregarding the primary mathematics curriculum, whereas the emphasis placed on knowledge and reasoning differs across grade levels. In primary schools, Pertiwi and Wahidin (2020) showed that fourth-grade assessments are dominated by number-related content, with far less attention given to geometry or data activities. These results reflect TIMSS’s framework, where knowing entails factual recall and procedural fluency, applying involves transferring knowledge to structured contexts, and reasoning requires non-routine problem-solving and critical thinking ( Peduk & Ateş, 2019). Importantly, the study also found that content domains exert a more positive effect on mathematics learner achievement than cognitive domains. This body of research provides an explanatory lens for South Africa’s TIMSS 2023 mathematics learner achievement. The relative strengths of South African Grade 5 learners in the application domain, alongside their persistent weaknesses in knowledge and reasoning, reflect the misalignment noted by several studies internationally, where instruction and textbooks emphasise routine application but fail to develop the basic knowledge and reasoning capacity required for learner progression. In contrast, Singapore’s balanced curriculum and pedagogy demonstrate how alignment across content and cognitive domains fosters sustained learner achievement.

### 1.5 TIMSS: A Diagnostic Instrument

TIMSS provides a structured empirical basis for analysing domain-specific performance patterns, beyond broad structural explanations. The comparison with Singapore shows that South Africa’s curriculum is superficially similar in content but not in cognitive expectations, especially when it comes to knowing and reasoning. This mismatch leaves learners underprepared for both academic progression and broader applications of mathematics in their everyday lives. The fact that Singapore’s Grade 4 learners significantly outperform South Africa’s Grade 5 learners further shows that the gap in mathematics achievement among learners is not simply attributable to learner age or exposure but to differences in curriculum coherence, cognitive scaffolding, and teacher training and development.

The TIMSS 2023 results highlight not just the scale of South Africa’s underperformance but also its domain-specific and cognitive weaknesses. While Singapore demonstrates how curriculum coherence and sustained teacher training and development can support an understanding of teaching and learning across content and cognitive domains, South Africa’s challenges are rooted in weak foundations, gaps in geometry and measurement, and systemic barriers in teacher training, development, and the classroom environment. Addressing these challenges requires targeted curriculum reforms in early-grade numeracy, curriculum streamlining, and teacher professional development, with a particular emphasis on geometry, critical thinking (reasoning), and basic knowledge. Context-sensitive adaptations, rather than comprehensive importation of Singapore’s model, offer a pathway for South Africa to strengthen learner trajectories in mathematics.

### 1.6 Conceptual Framework

This study is guided by the TIMSS conceptual model, which distinguishes between mathematical content domains (numbers, measurements, geometry, and data) and cognitive domains (knowing, applying, and reasoning) ( Mullis et al., 2020). These cognitive domains broadly align with established constructs of mathematical proficiency, including conceptual understanding, procedural fluency, and adaptive reasoning ( Findell et al., 2001). Together, these dimensions provide a diagnostic lens for examining not only what learners are expected to know but also how they engage with mathematical tasks at increasing levels of cognitive demand. In light of South Africa’s historically low mathematics achievement, this framework facilitates the identification of domain-specific deficiencies in foundational knowledge, procedural proficiency, and higher-order reasoning, which are frequently obscured by overall performance averages. Within this study, the TIMSS framework functions as the primary analytical structure, guiding the disaggregation of achievement results and the identification of domain-specific achievement gaps.

The study employs curriculum alignment theory, which emphasises the coherence between curricular intentions, classroom enactment, and assessment demands, to interpret these patterns ( Porter, 2002). Alignment theory posits that meaningful learning gains are most likely when the intended curriculum, the implemented curriculum, and the attained curriculum operate in concert. In this study, curriculum alignment theory serves as the principal interpretive lens, enabling explanation of why certain content and cognitive domains exhibit more pronounced achievement gaps than others. International evidence from high-performing systems such as Singapore illustrates how strong alignment is achieved through a spiral curriculum that revisits mathematical concepts at progressively higher levels of complexity, supported by the Concrete–Pictorial–Abstract (CPA) approach that scaffolds learning from concrete representations to abstract reasoning ( Leong et al., 2015; Lutfi & Dasari, 2024). In contrast, research on South African primary mathematics reveals an overloaded curriculum, limited instructional time for mastery of foundational domains, and persistent weaknesses in geometry and spatial reasoning, compounded by gaps in teacher content knowledge and pedagogical confidence ( Maqoqa, 2024; Taylor, 2021). Situating South Africa’s Grade 5 TIMSS 2023 performance against Singapore’s Grade 4 results therefore provides a comparative curriculum lens for examining how differences in sequencing, pacing, and instructional coherence shape progression from basic knowledge to application and reasoning. To ensure conceptual clarity and avoid theoretical fragmentation, the study is grounded in only two complementary frameworks. The TIMSS model structures the measurement and domain disaggregation, while curriculum alignment theory structures the interpretation of domain-specific disparities. This dual framework ensures both diagnostic precision and explanatory coherence without theoretical fragmentation.

**Figure 1.**
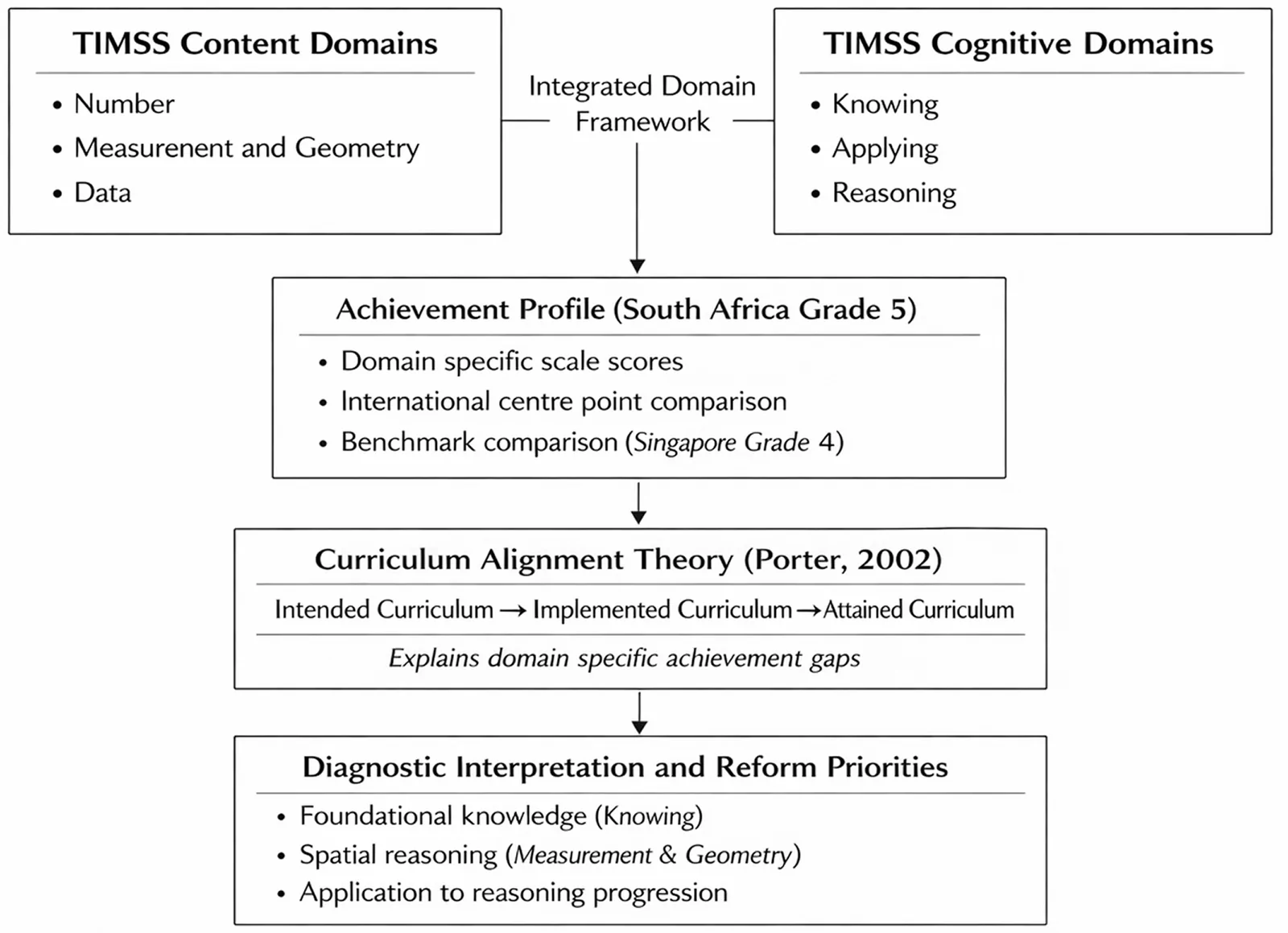
Adapted conceptual framework.

Figure 1 Conceptual framework integrating the TIMSS domain structure and Curriculum Alignment Theory guiding the diagnostic and interpretive analysis.

Figure 1 illustrates the streamlined conceptual framework, showing how the TIMSS domain structure and curriculum alignment theory interact to guide the analysis. Guided by this framework, the study’s methodology operationalises these dimensions through a secondary analysis of the TIMSS 2023 data. The TIMSS framework informs the analysis of content and cognitive domains, while curriculum alignment theory shapes the interpretation of performance patterns in relation to instructional coherence, learning progression, and assessment demands. The following section therefore outlines the research design, participants, data sources, analytical procedures, and ethical considerations employed in the study.

## 2. Methodology

### 2.1 Research Design

This study employed a quantitative secondary data analysis design, using data from the Trends in International Mathematics and Science Study (TIMSS) 2023. This design is particularly appropriate for investigating the mathematics achievement of South African Grade 5 learners across content and cognitive domains for several reasons. First, TIMSS provides a large, internationally standardised dataset that is both rigorous in design and nationally representative, making it suitable for examining learners’ performance patterns with a high degree of reliability. Second, secondary analysis enables the use of TIMSS’s robust psychometric procedures, including item response theory and plausible values, which strengthen the validity of inferences about learners’ achievement. Third, the TIMSS framework allows for meaningful international benchmarking, making it possible to situate South Africa’s performance in relation to high-performing education systems such as Singapore. Guided by the TIMSS assessment framework, the analysis focused on three content domains (numbers, measurement, geometry, and data) and three cognitive domains (knowing, applying, and reasoning). This two-fold focus not only enabled a diagnostic assessment of learners’ strengths and weaknesses within the South African context but also provided comparative insights to inform curriculum and policy reform globally. Importantly, the South African sample reported here reflects the TIMSS 2023 Grade 5 administration using the Grade 4 mathematics assessment instruments and framework. Within TIMSS procedures, some education systems assess an adjacent grade when curriculum alignment indicates that the benchmark framework better reflects learners’ opportunity to learn. Consequently, the comparison undertaken in this study is not between different grade curricula but between two cohorts assessed on the same TIMSS Grade 4 mathematics framework and common international reporting scale. The comparison does not assume equivalence in grade-level progression; rather, it examines relative performance under a common TIMSS measurement framework calibrated to ensure comparability of scale scores. The analysis therefore benchmarks Grade 5 in South Africa against Grade 4 in Singapore on an equivalent instrument, enabling comparison of performance patterns across TIMSS content and cognitive domains on the same international reporting scale ( Mullis et al., 2020; von Davier et al., 2024). It is important to emphasise that this design is descriptive and comparative rather than causal. The study identifies structured achievement gaps across domains but does not infer grade-level learning gains or causal mechanisms.

### 2.2 Participants

The South African TIMSS 2023 primary grade sample comprised 10,424 Grade 5 learners from 285 schools who were assessed using the TIMSS Grade 4 mathematics instruments, while Singapore assessed 6,530 Grade 4 learners from 181 schools using the same Grade 4 mathematics assessment framework ( Department of Basic Education, 2024; von Davier et al., 2024). The International Association for the Evaluation of Educational Achievement (IEA), in collaboration with Statistics Canada, used a two-stage stratified cluster sampling methodology to guarantee nationally representative estimates ( Siegel & Foy, 2024). In the first stage, schools were selected with probabilities proportional to their size, and in the second stage, intact Grade 5 classes were sampled. The stratification variables included the school sector (public or private), the language of instruction, the geographic region, socioeconomic indicators, the degree of urbanisation, and prior academic achievements (Ibid.). The use of stratified cluster sampling and population weights ensures that reported statistics represent national achievement distributions rather than sample-level estimates.

### 2.3 Data Collection and Analysis

Data for this study were drawn from the TIMSS 2023 mathematics assessment and associated contextual background questionnaires administered to participating learners, teachers, and schools. The TIMSS mathematics achievement is reported on an internationally standardised scale with a centre point of 500 and a standard deviation of 100, enabling valid comparisons across countries and education systems. The mathematics assessment comprised 183 items distributed across three content domains, namely number (94 items), measurement and geometry (49 items), and data (40 items), as well as three cognitive domains, namely knowing (58 items), applying (85 items), and reasoning (40 items) ( Reynolds, 2024). Each learner completed one assessment booklet, with achievement estimates derived using item response theory and reported as plausible values. This design supports reliable population-level estimation while minimising respondents’ burden ( von Davier, 2020).

The analysis focused on South Africa’s Grade 5 results, and Singapore’s Grade 4 performance was used as an international benchmark to contextualise domain-specific patterns of mathematics achievement. This comparison is consistent with TIMSS procedures, as both cohorts were assessed using the same Grade 4 mathematics framework and instruments, calibrated on a common international scale. Weighted descriptive statistics were computed in accordance with International Association for the Evaluation of Educational Achievement (IEA) guidelines to account for TIMSS’s two-stage stratified cluster sampling design. Sampling weights were applied to ensure nationally representative estimates and to correct for unequal probabilities of selection and non-response, thereby reducing bias in cross-national comparisons ( Siegel & Foy, 2024). All analyses incorporated the full set of plausible values, and estimates were combined using Rubin's rules in accordance with TIMSS technical procedures. To assess differences in performance across content and cognitive domains, weighted mean scores were compared and tested for statistical significance at the 1% level to account for multiple comparisons. In addition to significance testing, effect sizes (Cohen’s *d*) were calculated to evaluate the practical magnitude of observed differences between domains and between South Africa and Singapore. Variance estimation incorporated the TIMSS replicate weights to account for the two-stage stratified cluster sampling design. Standard errors and confidence intervals were computed using these replicate weights, ensuring appropriate estimation of sampling variability. The combined use of statistical significance and effect size estimation enabled a more substantively meaningful interpretation of performance gaps, beyond reliance on mean differences alone.

Although TIMSS 2023 collects extensive contextual information through learner, teacher, school, and curriculum questionnaires, the present study prioritised a diagnostic comparison of domain-specific achievement patterns. Consequently, contextual variables such as socioeconomic status, language of instruction, school resources, and teacher characteristics were not incorporated into multivariate or multilevel models in the main analysis. The exclusion of these variables from inferential modelling was deliberate, given that the primary objective was to identify and quantify achievement gaps across content and cognitive domains rather than to estimate explanatory or causal effects. Instead, these variables were used interpretively in the discussion to contextualise the observed performance trends. This analytic decision reflects a staged research logic: descriptive domain disaggregation precedes explanatory modelling. Such an approach is consistent with established practices in large-scale assessment research, where descriptive diagnostics and explanatory modelling are viewed as complementary rather than competitive strategies ( OECD, 2019; Rutkowski & Delandshere, 2016).

To ensure appropriate estimation of statistical uncertainty, all achievement comparisons were based on the full set of TIMSS plausible values. Estimates were combined using Rubin’s rules, as prescribed in IEA technical documentation, to account for measurement imputation variability inherent in plausible value methodology. Variance estimation was conducted in accordance with IEA technical guidelines for complex survey data. Weighted means were calculated using student sampling weights, and standard errors were derived using the TIMSS replicate weights to account for the two-stage stratified cluster sampling design.

Statistical inference was undertaken using these replicate-based variance estimates. Effect sizes (Cohen’s *d*) were reported alongside significance tests to provide an educationally meaningful interpretation of observed differences beyond statistical significance alone. Confidence intervals are reported in the supplementary materials to enhance transparency and facilitate replication of domain-level comparisons.

### 2.4 Ethical Considerations

This study is based on secondary analysis of TIMSS 2023 restricted-use datasets provided by the IEA. The data contain no personal identifiers and were collected under strict international ethical protocols during the original administration. Because this research involved secondary analysis of anonymised data, no institutional ethics approval was required. There was no formal request to use the dataset from the IEA since the data is available in the public domain, and all analyses adhered to its guidelines for responsible data use.

## 3. Results

This section presents the empirical results addressing Research Questions 1 and 2. Research Question 1 examines South Africa’s achievement profile across TIMSS mathematics content domains, while Research Question 2 examines achievement across cognitive domains. Research Question 3, concerning curriculum and pedagogical implications, is addressed in the Discussion section. For the purposes of this study, an “achievement gap” is operationally defined as (a) the scale score distance from the TIMSS international centre point of 500, and (b) the benchmark scale score difference between South African Grade 5 learners and Singaporean Grade 4 learners assessed on the same TIMSS Grade 4 mathematics framework. Effect sizes (Cohen’s *d*) are used to indicate the magnitude of these benchmark disparities.

### 3.1 Content Domain Achievement

In response to Research Question 1, the analysis reveals that South African Grade 5 learners perform substantially below Singaporean Grade 4 learners across all three TIMSS content domains: Number, Measurement and Geometry, and Data. These differences constitute substantial achievement gaps across all content domains rather than isolated performance variations. Relative to the TIMSS international centre point (500), South Africa’s mean performance in each content domain reflects a substantial negative deviation, indicating systemic underperformance rather than isolated topic weaknesses. The largest benchmark achievement gap is observed in Measurement and Geometry. This domain exhibits the greatest scale score distance between South Africa and Singapore and the largest effect size ( *d* = 2.66), indicating an extremely large practical disparity.  Table 1 reports the weighted mean scores and effect sizes for South African Grade 5 learners and Singaporean Grade 4 learners across the three TIMSS mathematics content domains.

**Table 1.** Mean Scale Scores and Effect Sizes in Mathematics Content Domains, TIMSS 2023

Content domain	Singapore (Grade 4)	South Africa (Grade 5)	Gap	Cohen’s *d*
Numbers	613	362	251	2.51
Measurement & Geometry	619	353	266	2.66
Data	616	362	254	2.54

*Note.* All estimates are weighted. Standard errors and confidence intervals were computed using TIMSS plausible values and replicate weights and are reported in the supplementary materials.

All three domains reveal large effect sizes ( *d* > 2.5), signifying deep and systematic achievement gaps across the mathematics curriculum. The concentration of the largest gaps in measurement and geometry indicates that spatial reasoning, geometric visualisation, and conceptual understanding in this domain represent the most acute areas of weakness within the South African achievement profile. The characteristics of this gap indicate constraints in students' capacity to interact with spatial and relational concepts that necessitate conceptual integration rather than mere procedural recall. Importantly, while performance is low across domains, the relative ordering of domain means indicates that the gap is not uniform; it is most severe in Measurement and Geometry and comparatively less pronounced, though still substantial, in Number and Data.

### 3.2 Cognitive Domain Achievement

Addressing Research Question 2, the results indicate that South African learners demonstrate markedly lower performance than Singaporean learners across all three cognitive domains: knowing, applying, and reasoning. These results reveal pronounced cognitive achievement gaps across all levels of mathematical thinking. The Knowing domain exhibits the largest cognitive achievement gap, with an effect size of *d* = 2.67. This domain captures factual recall, procedural fluency, and basic computational knowledge. The magnitude of this disparity indicates a profound benchmark gap in foundational mathematical knowledge.

**Table 2.** Mean Scale Scores and Effect Sizes in Mathematics Cognitive Domains, TIMSS 2023.

Cognitive domain	Singapore (Grade 4)	South Africa (Grade 5)	Gap	Cohen’s *d*
Knowing	624	357	267	2.67
Applying	615	366	249	2.49
Reasoning	609	363	246	2.46

*Note.* All estimates are weighted. Standard errors and confidence intervals were computed using TIMSS plausible values and replicate weights and are reported in the supplementary materials.

While performance in the applying domain (mean = 366) is relatively stronger than in knowing or reasoning within the South African profile, it remains substantially below both the international centre point and the Singapore benchmark. This indicates that even where relative strengths exist, significant achievement gaps persist when evaluated against international standards. This pattern indicates a differentiated achievement profile in which routine procedural engagement is comparatively more accessible than foundational fluency or non-routine reasoning. The reasoning domain also reflects a large achievement gap relative to Singapore. The combined pattern across cognitive domains indicates that foundational knowledge deficits are accompanied by substantial limitations in higher-order reasoning, with the largest disparity concentrated at the most basic cognitive level (knowing). This layered structure of gaps suggests that weaknesses in foundational knowledge constrain progression to more complex forms of mathematical reasoning, which may ultimately hinder students' overall mathematical performance and their ability to solve advanced problems effectively.

### 3.3 Item-Level Illustrations

To further illustrate the domain-specific patterns identified in Research Questions 1 and 2, selected released TIMSS items are used to demonstrate how content and cognitive disparities manifest at the task level. In the Knowing domain (Number), more than 90 percent of Singaporean learners correctly recalled basic multiplication facts, compared to fewer than 40 percent of South African learners. This example illustrates a substantial foundational achievement gap in procedural fluency. In the applying domain (data), routine tasks such as interpreting a simple bar chart were accessible to a proportion of South African learners, indicating some competence in structured and familiar problem contexts. However, in the reasoning domain (geometry), where items required multi-step reasoning with angle relationships, fewer than 20 percent of South African learners responded correctly, compared with a majority of Singaporean learners. This reflects a pronounced achievement gap in tasks requiring conceptual reasoning and spatial integration. These illustrative items reinforce the quantitative results by showing that the largest cognitive and content disparities are concentrated in foundational recall and spatial reasoning tasks requiring conceptual integration rather than routine execution alone. Collectively, these examples highlight how curriculum exposure and classroom instructional practices shape learners’ preparedness to engage with domain-specific cognitive demands.

### 3.4 Visualising the Gaps

**Figure 2.**
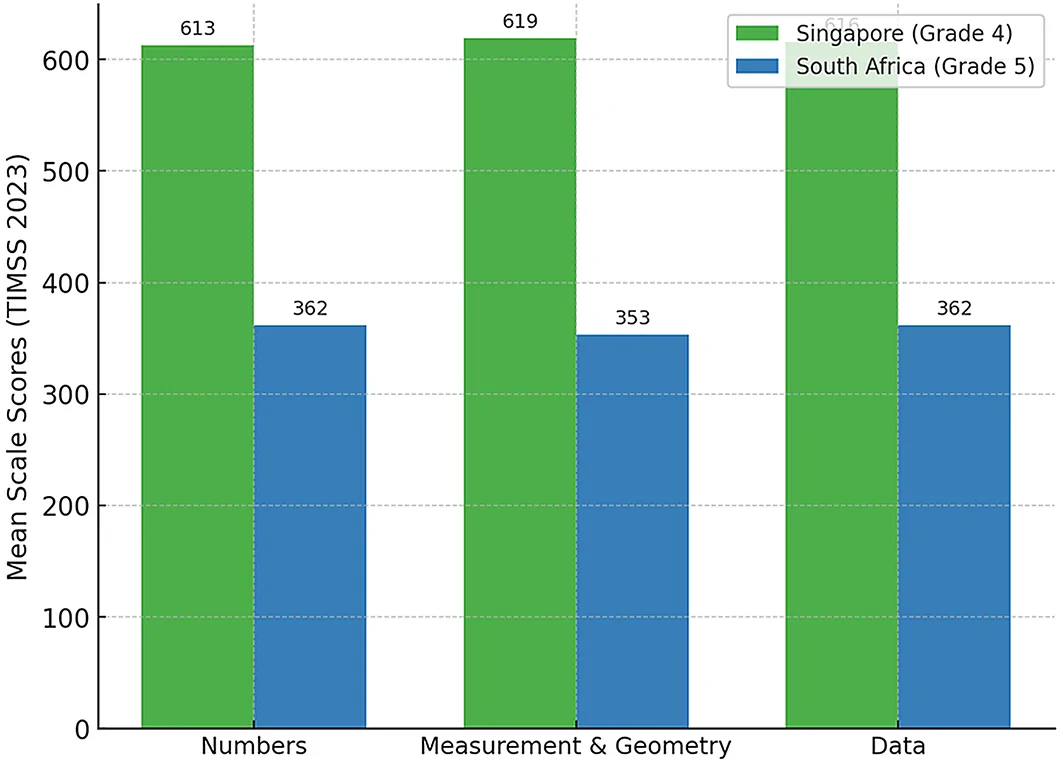
Comparative performance of South African Grade 5 and Singaporean Grade 4 learners across TIMSS content domains (Numbers, Measurement & Geometry, and Data).

Figure 2 illustrates the comparative performance of South African Grade 5 and Singaporean Grade 4 learners across the three TIMSS content domains. The visualisation confirms the consistently lower achievement of South African learners, with the largest gap observed in Measurement and Geometry. This pattern aligns with the tabulated results and underscores the concentration of content-domain disparities in spatial reasoning and geometric understanding. By presenting scale score distances graphically, Figure 2 highlights the distribution and magnitude of domain-specific achievement gaps rather than suggesting uniform underperformance across all content areas.

**Figure 3.**
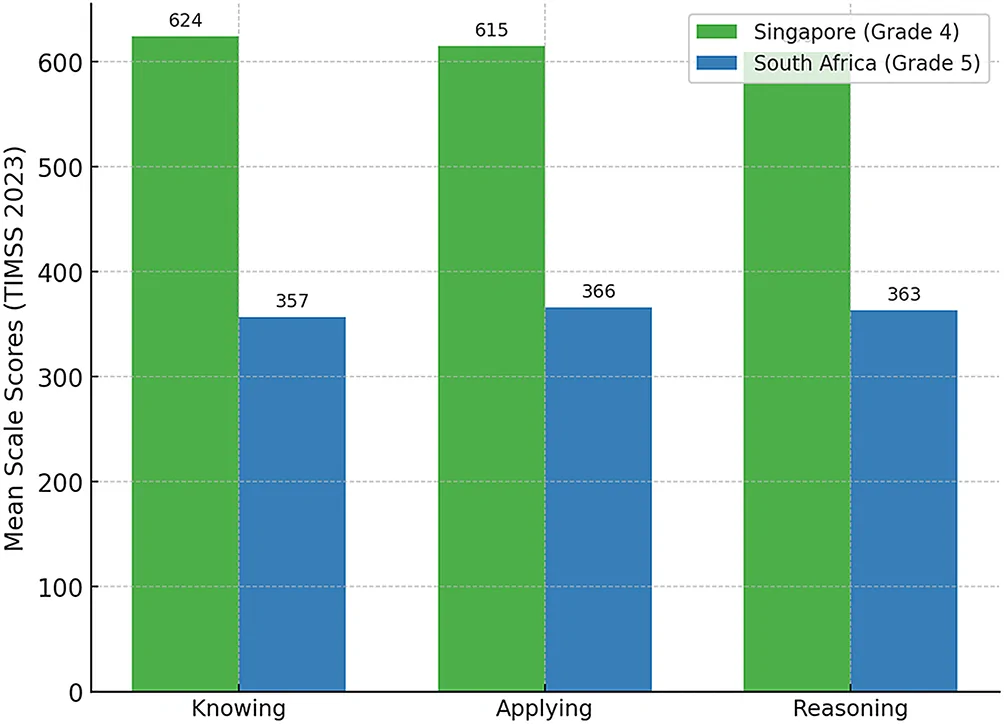
Comparative performance of South African Grade 5 and Singaporean Grade 4 learners across TIMSS cognitive domains (Knowing, Applying, and Reasoning).

Figure 3 presents the comparative performance of South African Grade 5 and Singaporean Grade 4 learners across the TIMSS cognitive domains. The figure clearly indicates that the widest cognitive disparity occurs in the Knowing domain, reflecting substantial weaknesses in foundational fluency. Although the Applying domain appears relatively stronger within the South African profile, it remains substantially below benchmark levels, indicating that relative strength does not imply adequacy relative to international expectations. Together, the figures visually reinforce the empirical finding that achievement gaps are systematic, domain-specific, and concentrated in foundational knowledge and spatial reasoning.

### 3.5 Summary of Results

Synthesising the results addressing Research Questions 1 and 2, the results highlight three critical insights into South African learners’ mathematics achievement. First, geometry and spatial reasoning continue to represent the weakest domain, pointing to enduring structural gaps in both curriculum design and teacher preparation. Second, the severity of deficits in foundational knowledge, as indicated in the knowing domain, hinders learners' advancement to higher-order reasoning tasks. Third, although learners demonstrate relatively positive learner achievement in the applying domain, this potential remains constrained by the absence of solid basic skills and limited opportunities for reasoning skills, which prevents the development of sustained mathematical learner achievement. These results collectively indicate that achievement gaps are not uniform but are concentrated in foundational knowledge and spatial reasoning domains. The benchmark comparison with Singapore highlights the magnitude of these domain-specific disparities and provides a structured reference point for interpreting their educational significance.

## 4. Discussion

This section addresses Research Question 3 by interpreting domain-specific and cognitive performance patterns in relation to curriculum alignment, pedagogy, and teacher professional development and outlining implications for improving mathematics achievement in South Africa. The TIMSS 2023 results reaffirm that South African Grade 5 learners perform substantially below the international mathematics benchmark, achieving a mean score of 362, compared with the TIMSS centre point of 500. However, the more analytically significant finding lies not in the overall mean alone but in the concentration and distribution of achievement gaps across specific content and cognitive domains. These comparisons are interpreted within a common TIMSS measurement framework and do not imply equivalence in grade-level progression; rather, they provide a structured benchmark for examining domain-specific achievement patterns. Disaggregating achievement reveals that the largest content-domain gap occurs in measurement and geometry, while the largest cognitive-domain gap occurs in knowing. These results are consistent with prior research highlighting persistent domain-specific weaknesses rather than uniform underperformance across mathematics ( Maqoqa, 2024; Taylor, 2021). These domain-specific disparities provide a more precise diagnostic basis for interpreting systemic challenges.

### 4.1 Content-Domain Patterns and Foundational Gaps

The largest benchmark gap was observed in measurement and geometry, where effect sizes indicated a large disparity relative to Singapore. This result indicates that spatial reasoning, geometric visualisation, and conceptual understanding constitute the most acute areas of weakness in the South African achievement profile. While performance in Number and Data also remains substantially below international expectations, the relative ordering of domain means indicates that underperformance is not uniform across content areas. The disproportionate weakness in measurement and geometry is consistent with national studies identifying geometry as a longstanding area of difficulty in South African classrooms ( Maqoqa, 2024; Taylor, 2021). These results do not indicate a lack of geometry instruction; instead, they suggest a possible misalignment between the intended curriculum and its implementation in the classroom. Consistent with Curriculum Alignment Theory ( Porter, 2002), gaps may emerge when intended learning outcomes emphasise conceptual reasoning, while implemented instruction remains focused on procedural routines. This interpretation extends prior research by demonstrating how such misalignments manifest empirically in large-scale assessment data. Strengthening geometry education therefore requires approaches that prioritise visualisation, contextualisation, and progressive abstraction. The empirical evidence indicates that targeted improvement in spatial reasoning should be treated as a priority domain rather than as one component among equally distributed weaknesses.

### 4.2 Cognitive-Domain Performance and Learning Progression

In further addressing research question 3, the largest cognitive achievement gap was observed in the Knowing domain. South African learners scored substantially lower than Singaporean learners (357 versus 624), with a very large effect size. Because the Knowing domain captures factual recall and procedural fluency, this result reflects a pronounced gap in foundational knowledge. In the TIMSS framework, foundational fluency is the basis for moving on to tasks that require applying and reasoning. The magnitude of the knowing gap is therefore associated with the overall cognitive achievement profile. Although applying represents a relatively stronger domain within the South African distribution, it remains substantially below international benchmarks. These results indicate that learners may engage with familiar, structured procedures, but their capacity to generalise, justify, or reason abstractly appears limited in relation to their foundational fluency levels. This pattern is consistent with prior results that procedural competence without conceptual depth does not reliably support sustained reasoning development ( Mullis & Martin, 2017). The combined domain pattern therefore reflects a vertically constrained achievement profile, in which weaknesses at the most basic cognitive level are associated with reduced engagement with higher-order reasoning. These results extend prior work by demonstrating how domain-specific disparities in foundational knowledge correspond with differentiated cognitive performance patterns across applying and reasoning domains.

### 4.3 Curriculum Alignment and Cross-National Insights

In relation to Research Question 3, the benchmark comparison with Singapore illustrates how curriculum alignment may be associated with domain-specific achievement distributions. Singapore’s tightly sequenced spiral curriculum and systematic use of the Concrete–Pictorial–Abstract progression support continuity across content and cognitive domains ( Leong et al., 2015). This aligns with international evidence that coherent curriculum progression and structured pedagogical scaffolding are associated with balanced development across knowledge, application, and reasoning domains. In contrast, South Africa’s curriculum has been described as broad in scope, with limited instructional time for consolidation of foundational domains ( Maqoqa, 2024; Taylor, 2021). A lack of sufficient depth may constrain cumulative mastery, especially in domains like geometry that require sustained conceptual development. Curriculum Alignment Theory ( Porter, 2002) indicates that meaningful gains are most likely when the intended, implemented, and assessed curricula are coherently aligned. Therefore, partial misalignments between policy aspirations, classroom practice, and assessment demands may be associated with the observed achievement gaps in knowing, measurement, and geometry. These results extend prior work by indicating that such misalignments correspond with clearly differentiated domain-specific achievement patterns rather than uniform underperformance. This interpretation situates the present results within a broader body of curriculum alignment research, highlighting the consequences of systemic incoherence. Importantly, the comparison does not imply direct causal attribution but rather provides a structured reference point for interpreting domain-specific disparities.

### 4.4 Interpreting Singapore as a Benchmark

In relation to Research Question 3, Singapore’s performance provides a valuable benchmark for examining curriculum coherence and learning progression, but it is essential to recognise the contextual conditions under which it was achieved. Singapore's education system functions within a highly structured and competitive framework, characterised by strict central curriculum oversight, selective teacher recruitment, rigorous professional training, and elevated societal expectations concerning academic performance ( Ng, 2017). These features are accompanied by early differentiation and sustained academic pressure, which, although associated with high achievement, may also generate stress and equity concerns that are not fully captured in large-scale assessment data ( Tan, 2018). The value of Singapore as a comparator resides not in direct policy transplantation but in the identification of transferable principles, including curriculum coherence, focused content progression, and pedagogical scaffolding ( Deng et al., 2013). For South Africa, such an approach implies selective and context-sensitive adaptation rather than wholesale adoption, taking into account systemic capacity, linguistic diversity, and resource constraints ( Spaull & Kotze, 2015). The benchmark therefore functions analytically, providing scale and distributional reference rather than prescriptive replication.

### 4.5 Implications for Policy and Practice

In relation to Research Question 3, these results point to a dual challenge. First, persistent weaknesses in number sense, procedural fluency, and spatial reasoning are evident and warrant focused instructional attention. Second, learners’ relative strength in applying may represent a potential entry point for developing higher-order reasoning, provided that foundational knowledge is more consistently supported and instructional practices are orientated toward conceptual engagement. Addressing these challenges requires coordinated consideration across curriculum design, teacher professional development, and classroom practice. These results align with existing research, which emphasises the importance of integrating foundational skill development with opportunities for conceptual reasoning. Reducing class sizes and increasing specialist support remain important considerations, but they may face short-term financial or political constraints, which could hinder their implementation in the immediate future. Targeted diagnostic assessment, structured small-group instruction, and school-based professional learning communities offer contextually feasible strategies that may support incremental improvement in student learning outcomes within the constraints of existing educational contexts and resources.

### 4.6 Pillars for Strengthening Foundational Mathematics

**Pillar 1: Strengthening Foundations through Diagnostic Teaching**

The pronounced deficits in the knowing domain indicate that many learners progress without secure mastery of basic mathematical foundations. Early diagnostic assessment in the foundation and intermediate phases can help identify learning gaps before they compound. Adapted support programmes, combined with teacher development in formative assessment and error analysis, can translate diagnostic information into responsive classroom practices.

**Pillar 2: Enhancing Geometry and Spatial Reasoning**

Given the consistently poor performance in measurement and geometry, professional development should prioritise visual, experiential, and problem-based approaches to spatial reasoning. In resource-constrained contexts, locally available materials and outdoor measurement activities can provide effective entry points, supplemented over time by affordable digital visualisation tools.

**Pillar 3: Leveraging Application to Cultivate Reasoning**

The comparatively stronger performance in applying suggests an opportunity to scaffold reasoning through contextually relevant problem-solving tasks. Supporting teachers as they design inquiry-based lessons that emphasise explanation, justification, and collaborative reasoning can help bridge the gap between application and higher-order thinking.

### 4.7 System-Level Coherence

The effectiveness of these priorities depends on systemic alignment across phases. Consistent with Curriculum Alignment Theory, sustainable improvement requires coherence between curriculum intent, classroom enactment, assessment design, and teacher preparation. The domain-specific gaps identified in this study underscore that improvement is unlikely to result from isolated interventions. Instead, targeted strengthening of foundational fluency and spatial reasoning, supported by aligned curriculum and professional development structures, is necessary to shift the achievement profile. When curriculum goals, pedagogical support, and assessment expectations converge, foundational mathematics instruction can move beyond procedural compliance toward deeper conceptual engagement, contributing to more equitable learning trajectories in alignment with Sustainable Development Goal 4.

## 5. Conclusion

This study examined South African Grade 5 learners’ mathematics achievement in TIMSS 2023, disaggregated by content and cognitive domains and benchmarked against Singapore’s Grade 4 performance on the same TIMSS Grade 4 mathematics framework. Rather than relying solely on overall mean comparisons, the study identified the distribution and concentration of achievement gaps across specific domains. Consistent with earlier research ( Mabena et al., 2021; Taylor, 2021), the results confirm that South Africa continues to perform substantially below international benchmarks. However, the more diagnostic contribution of this study lies in demonstrating that the largest content-domain gap occurs in measurement and geometry, while the largest cognitive-domain gap occurs in knowing. These domain-specific disparities are most evident in foundational procedural fluency and spatial reasoning within the national achievement profile.

The results further indicate that South African learners demonstrate comparatively stronger performance in the 'Applying' domain than in 'Knowing' or 'Reasoning' within their internal distribution. Although this relative positioning does not reduce the magnitude of the benchmark gap, it points to structured procedural engagement as a potential area of instructional focus, particularly in enhancing learners' foundational procedural fluency and spatial reasoning skills. Interpreted through the TIMSS domain framework and Curriculum Alignment Theory ( Porter, 2002), the results indicate that achievement gaps are not uniformly distributed but are concentrated in foundational and spatial domains that are critical for progression to higher-order reasoning. The study does not identify causal mechanisms; instead, it offers a structured diagnostic profile that can guide focused curriculum and pedagogical decisions.

Building on these empirical insights, three interrelated priorities emerge: strengthening foundational fluency through early diagnostic assessments and targeted support, prioritising spatial reasoning and conceptual geometry instruction, and supporting the progression from application to reasoning through tasks requiring explanation and justification. Sustainable improvement is unlikely to result from isolated interventions. Consistent with Curriculum Alignment Theory, meaningful gains are more likely when curriculum design, assessment expectations, classroom enactment, and teacher development operate coherently. Therefore, the results draw attention to the importance of domain-specific reform efforts grounded in empirical achievement profiles rather than generalised prescriptions. By aligning curriculum intent with classroom practice and assessment demands, South Africa is better positioned to strengthen foundational mathematics instruction in ways that promote deeper conceptual engagement and contribute to more equitable learning trajectories in line with Sustainable Development Goal 4.

## 6. Limitations and Future Research

While this study offers significant insights into Grade 5 mathematics achievement in South Africa, it is important to acknowledge several limitations. First, the analysis relied on secondary, cross-sectional data from TIMSS 2023, which precludes causal inference. Consequently, the associations identified between content and cognitive domains should be regarded as descriptive rather than causal relationships. While such evidence is valuable for diagnostic and policy-relevant comparison, it cannot on its own establish mechanisms of effect.

Second, domain-specific subscales, particularly those for knowing and measurement and geometry, are based on fewer test items than the overall mathematics scale, which may reduce measurement precision. Although the use of plausible values enhances the reliability of population-level estimates, it also introduces statistical uncertainty that should be considered when interpreting effect sizes and domain-level differences.

Third, the use of large-scale assessment data limits direct examination of classroom processes, instructional strategies, and learner experiences that shape achievement. Without qualitative or longitudinal evidence, it is not possible to observe how curriculum intentions are enacted in practice or how learners engage with mathematical tasks over time. Future research would benefit from mixed-methods designs that integrate TIMSS data with classroom observations, teacher interviews, and learner case studies to deepen explanatory insight.

Finally, although TIMSS sampling ensures national representativeness, it may under-represent learners in marginalised or remote contexts characterised by multigrade teaching, language-of-instruction challenges, and severe resource constraints. Targeted studies and oversampling in such settings could provide a more nuanced perspective on how structural inequalities shape foundational learning trajectories.

Future research should therefore extend this work through longitudinal, multilevel, and design-based studies that examine how targeted interventions in foundational fluency and spatial reasoning influence learner trajectories over time. Experimental or quasi-experimental evaluations of diagnostic teaching approaches and geometry-focused professional development would provide stronger evidence regarding the mechanisms underlying domain-specific improvement. By integrating descriptive large-scale assessment diagnostics with explanatory and intervention research, a more comprehensive evidence base can be developed to inform curriculum reform, teacher education, and policy innovation aimed at improving mathematics achievement and equity in South Africa.

## Author Contributions

The author, Mathelela Steyn Mokgwathi, is responsible for the conceptualisation, data curation, formal analysis, investigation, methodology, project administration, resources, software, supervision, validation, visualisation, and writing of the original draft, as well as the writing, review, and editing processes.

## Declaration of AI Use

The author affirms that no generative artificial intelligence tools (such as ChatGPT or similar models) were used to produce the academic content, analysis, or interpretations presented in this manuscript. QuillBot (premium) was employed solely for grammar and spelling checks. The author personally reviewed and edited the final manuscript and takes full responsibility for its content and conclusions.

## Data Availability

The data that support the results of this article are derived from the publicly accessible Trends in International Mathematics and Science Study (TIMSS) 2023 database, available through the International Association for the Evaluation of Educational Achievement (IEA) at https://timssandpirls.bc.edu. Derived and processed data underlying the statistical analyses, including the variables used to generate the tables, figures, and descriptive statistics presented in this article, are openly available in the Zenodo repository ( Mokgwathi, 2025) at https://doi.org/10.5281/zenodo.17379412, under the Creative Commons Attribution 4.0 International (CC BY 4.0) licence. These files include anonymised values underlying the means, standard deviations, and effect sizes, as well as detailed variable descriptions and methodological notes supporting replication of the comparative analyses between South Africa (Grade 5) and Singapore (Grade 4). No ethical approval or participant consent was required, as all data originate from secondary sources that are publicly available through the IEA TIMSS 2023 repository. Supplementary materials supporting this article are available in the Zenodo repository at https://doi.org/10.5281/zenodo.17379412 ( Mokgwathi, 2025), under the CC BY 4.0 license. These include: TIMSS2023_SGvZA_Derived_Dataset.xlsx (domain-level statistics and effect sizes), TIMSS2023_Variable_Descriptions.xlsx (variable definitions and sources), and TIMSS2023_Supplementary_Notes.pdf (methodological documentation).
