# Closing the Gap in Early Mathematics: Domain and Cognitive Insights from TIMSS 2023 in South Africa and Singapore

**DOI:** 10.12688/f1000research.172015.4

**Published:** 2026-09-12

**Authors:** Mathelela Steyn Mokgwathi

**Affiliations:** 1 Department of Early Childhood Education, University of South Africa, Pretoria, Gauteng, South Africa

**Keywords:** Trends in International Mathematics and Science Study (TIMSS), Mathematics Achievement, Content and Cognitive Domains, South Africa, Singapore

## Abstract

**Background:**

South Africa continues to underperform in primary mathematics, yet overall mean comparisons obscure where achievement gaps are most concentrated. This study examines domain-specific patterns of mathematics achievement using data from the Trends in International Mathematics and Science Study (TIMSS) 2023, benchmarking South African Grade 5 learners against Singaporean Grade 4 learners assessed on the same TIMSS Grade 4 mathematics framework.

**Methods:**

A quantitative secondary analysis was conducted using nationally representative TIMSS 2023 datasets comprising 10,424 South African learners from 285 schools and 6,530 Singaporean learners from 181 schools. South Africa assessed learners in Grade 5 using the TIMSS Grade 4 mathematics instruments, consistent with TIMSS procedures where curriculum exposure aligns with the benchmark framework. Mathematics achievement was analysed across content domains (Number, Measurement and Geometry, Data) and cognitive domains (Knowing, Applying, Reasoning). Weighted estimates, replicate-based variance estimation, and effect sizes (Cohen’s d) were computed in accordance with IEA technical guidelines. The analysis is descriptive and comparative rather than causal.

**Results:**

South African learners performed substantially below the international centre point across all domains. The largest content-domain gap relative to Singapore was observed in Measurement and Geometry, indicating pronounced weaknesses in spatial reasoning. The largest cognitive-domain gap occurred in Knowing, reflecting fragile foundational fluency. Although Applying was relatively stronger within the South African profile, it remained substantially below benchmark levels. Achievement gaps were therefore concentrated in foundational and spatial domains rather than uniformly distributed.

**Conclusions:**

Concentrated achievement gaps in foundational knowledge and geometry characterise the underperformance of primary mathematics in South Africa. The findings provide a structured diagnostic profile of domain-specific achievement disparities, highlighting the concentration of gaps in foundational knowledge and spatial reasoning.

## 1. Introduction

Mathematics achievement at the primary school level has profound implications for learners’ future participation in education, work, and society. Basic mathematics skills support higher-order thinking, problem-solving, and lifelong learning, all of which are central to academic success and broader social participation, as consistently demonstrated in international large-scale assessment research ( Mullis et al., 2020; von Davier et al., 2024). TIMSS has become a critical assessment instrument for benchmarking mathematical learner achievement internationally, providing comparative evidence of mathematics achievement across education systems with different curricular and instructional contexts. Since the advent of democracy, South Africa has expanded access to schooling and implemented curriculum reforms aimed at promoting more equitable learning opportunities. South Africa has participated in TIMSS since 1995 and, despite modest gains over successive cycles, continues to record one of the lowest average mathematics achievement scores among participating education systems ( Mullis et al., 2020; Reddy & Hannan, 2019; Zuze et al., 2018). National average performance, however, masks substantial variation associated with socioeconomic background, language, school contexts, and other structural inequalities. Much of the existing research focuses on Grade 9 data, emphasising long-term learning deficits and systemic inequities ( Mensah & Baidoo-Anu, 2022; Reddy & Hannan, 2019). Far less attention has been given to the primary phase, particularly Grade 5, where learners consolidate fundamental numeracy and begin to transition from concrete to abstract reasoning.

Despite extensive research on mathematics achievement in South Africa, much of the existing literature has concentrated on overall performance trends or on secondary school outcomes, particularly at the Grade 9 level, where cumulative learning deficits become more visible. Comparatively limited attention has been given to domain-specific achievement patterns at the primary level, where foundational competencies are established. Furthermore, while international benchmarking studies highlight performance disparities, fewer studies systematically disaggregate these gaps across both content and cognitive domains using a unified analytical framework. This limits the ability to identify where learning deficits are most concentrated and how these deficits are distributed across different areas of mathematics learning. The present study addresses this gap by providing a structured domain-level analysis of Grade 5 mathematics achievement using TIMSS 2023 data.

TIMSS 2023 results indicate that South African Grade 5 learners achieved an average score of 362 compared to Singapore’s Grade 4 average of 615 ( von Davier et al., 2024). This gap, despite Singaporean learners being a grade lower than South African learners, indicates substantial differences in average mathematics achievement between the two education systems. More specifically, this disparity reflects structured achievement gaps across both mathematical content domains and cognitive domains, rather than a uniform decline across all aspects of mathematics learning. Preliminary TIMSS 2023 reporting shows that the lowest average performance occurred in Measurement and Geometry and in the Knowing cognitive domain. These patterns are consistent with comparatively weaker performance in spatial reasoning and procedural fluency.

In TIMSS 2023, while the international benchmarking grades are 4 and 8, some education systems assess an adjacent grade using the same internationally calibrated instruments when curriculum alignment indicates that the benchmark framework better matches learners’ opportunity to learn. In this study, South Africa’s Grade 5 cohort is analysed because it was assessed using the TIMSS Grade 4 mathematics assessment instruments and framework, providing a common assessment framework for comparison with Singapore’s Grade 4 results ( Mullis et al., 2020; von Davier et al., 2024). Accordingly, the comparison undertaken in this study is not between different curricula, but between two cohorts assessed on the same TIMSS Grade 4 mathematics framework and reporting scale, thereby ensuring measurement equivalence. Singapore was selected as the benchmark because it consistently demonstrates the highest average mathematics achievement in TIMSS. The comparison is intended to provide an analytical benchmark for examining domain-specific achievement gaps rather than to imply equivalence between the two education systems or to advocate direct transfer of educational policies and practices. Given the substantial historical, cultural, socioeconomic, and educational differences between South Africa and Singapore, the findings are interpreted to inform context-sensitive curriculum and pedagogical improvement rather than direct policy adoption.

National assessments such as the Annual National Assessments (ANA), which is now discontinued, and systemic evaluation reports have highlighted low achievement levels in mathematics among learners, but they rarely explore how learners perform across specific content and cognitive domains. Yet TIMSS distinguishes between three content domains (Numbers, Measurement and Geometry, and Data) and three cognitive domains (Knowing, Applying, and Reasoning). Assessing learner achievement through these lenses allows for a diagnostic understanding of learners’ strengths and weaknesses. In this study, the term “achievement gap” refers to measurable differences in scale scores relative to the TIMSS international centre point and relative to a high-performing benchmark system on the same assessment framework. The focus is therefore diagnostic and comparative rather than causal. Research from high-performing education systems such as Singapore describes curriculum alignment, spiral progression, and scaffolded teaching and learning as features associated with balanced development of knowledge, application, and reasoning skills ( Choy & Dindyal, 2024; Low & Wong, 2021; Morony, 2023; Mullis et al., 2020). These features provide valuable comparative insights; however, educational practices are embedded within specific historical, cultural, and institutional contexts and therefore require careful contextual adaptation rather than direct transfer. Conversely, South African studies identify persistent challenges in geometry, reasoning, and teacher content knowledge ( Maqoqa, 2024; Taylor, 2021), compounded by curriculum overload and large class sizes, which are associated with fewer opportunities for formative assessment and conceptual engagement **.** Improving mathematics learner achievement has been associated with more than curriculum reform; it depends on strengthening the consistency between curriculum design and development, teacher professional development, and classroom practice. Examining how learners perform across content and cognitive domains helps identify the areas requiring targeted instructional support and pedagogical innovation.

This study therefore aims to examine South African Grade 5 learners’ mathematics achievement in TIMSS 2023, disaggregated by content and cognitive domains, and benchmarked against Singapore’s Grade 4 performance on the same TIMSS Grade 4 mathematics framework. By identifying where achievement gaps are most pronounced, the study seeks to provide a domain-specific diagnostic profile to inform curriculum alignment and pedagogical reform. It contributes in three key ways. Firstly, it focuses on the under-researched area of upper primary mathematics learning, where learners consolidate foundational numeracy and transition from concrete to more abstract mathematical reasoning. Secondly, it breaks down performance by content and cognitive areas to identify specific patterns of strength and weakness. Thirdly, it uses Singapore as an analytical benchmark to contextualise South Africa’s domain-specific achievement profile within a common international assessment framework. The study was guided by the following research questions:

### 1.1 Research Questions

1. What is the achievement profile of South Africa’s Grade 5 learners across the TIMSS 2023 Grade 4 mathematics content domains (Number, Measurement and Geometry, and Data)?
2. What is the achievement profile of South Africa’s Grade 5 learners across the TIMSS 2023 Grade 4 mathematics cognitive domains (Knowing, Applying, and Reasoning)?

### 1.2 Literature Review: Benchmarking South Africa’s Grade 5 Results on the TIMSS Primary Mathematics Assessment against Singapore’s Grade 4

***Benchmarking with TIMSS 2023***

The Trends in International Mathematics and Science Study (TIMSS) provides an internationally comparable assessment of mathematics and science achievement at the primary and secondary school levels ( Mullis et al., 2020; von Davier et al., 2024). Singapore consistently records the highest average mathematics achievement among participating education systems. In TIMSS 2023, Singapore’s Grade 4 learners achieved an average score of 615, while South Africa’s Grade 5 learners achieved an average score of 362 ( von Davier et al., 2024). Although Singaporean learners were assessed one grade lower than South African learners, both cohorts completed the internationally calibrated TIMSS Grade 4 mathematics assessment, providing a common framework for comparison. The comparison therefore serves as an analytical benchmark for examining domain-specific achievement patterns rather than as evidence that the two education systems are directly comparable in all respects. Given the substantial historical, cultural, socioeconomic, and educational differences between the two countries, differences in average achievement should be interpreted cautiously and within their respective educational contexts. The magnitude of the achievement gap highlights the value of examining performance across the TIMSS content domains (Number, Measurement and Geometry, and Data) and cognitive domains (Knowing, Applying, and Reasoning) to better understand domain-specific patterns of learner achievement.

***Curriculum Alignment and Content Domains***

Singapore’s mathematics curriculum is internationally recognised for its coherence and spiral structure, with mathematical concepts revisited at progressively higher levels of complexity. Previous research describes this curriculum structure as supporting continuity in learners’ mathematical development ( Choy & Dindyal, 2024; Low & Wong, 2021). In TIMSS 2023, Singapore scored 613 in Number, 619 in Measurement and Geometry, and 616 in Data, while South Africa scored 362, 353, and 362, respectively ( von Davier et al., 2024). These results indicate substantial differences in average achievement across all three content domains, with the largest difference observed in Measurement and Geometry. Maqoqa (2024) and Tachie (2020) similarly identify Measurement and Geometry as an area in which South African learners experience persistent learning challenges. The convergence between the TIMSS 2023 findings and previous South African research suggests that Measurement and Geometry remains an important area for curriculum and instructional attention. Although Singapore consistently records higher achievement across all content domains, these differences should be interpreted within the broader educational contexts of the two countries rather than as evidence that any single curricular feature explains the observed performance differences.

***Cognitive Demands: Knowing, Applying, and Reasoning***

TIMSS distinguishes between three cognitive domains: Knowing, Applying, and Reasoning, which assess learners’ ability to recall and apply mathematical knowledge and to solve increasingly complex problems ( Mullis et al., 2020). Singapore’s Grade 4 learners achieved average scores of 624 in Knowing, 615 in Applying, and 609 in Reasoning, whereas South Africa’s Grade 5 learners scored 357, 366, and 363, respectively ( von Davier et al., 2024). These results indicate substantial differences in average achievement across all three cognitive domains, with the largest difference observed in the Knowing domain (267 points). Within the South African cohort, the Applying domain recorded the highest average score, while the Knowing domain recorded the lowest average score. This pattern suggests variation in learners’ performance across the cognitive domains rather than uniform performance across all aspects of mathematical cognition. Previous studies have associated strong performance in the Knowing domain with secure foundational mathematical knowledge, while the Applying and Reasoning domains require learners to transfer and extend that knowledge in increasingly complex contexts ( Mullis et al., 2020; Peduk & Ateş, 2019). The comparatively lower performance observed in the Knowing domain therefore highlights an area that warrants further attention when interpreting South Africa’s overall mathematics achievement profile.

### 1.3 Instructional and Structural Factors

Several systemic factors have been associated with differences in mathematics achievement across education systems. According to Meier and West (2020), South African classrooms often experience overcrowding, with class sizes averaging over 50 learners, which may reduce opportunities for formative feedback and individualised learner support. Teacher content knowledge and pedagogical content knowledge also vary across schools, particularly in Measurement and Geometry ( Bhagwonparsadh & Pule, 2024; Taylor, 2021). These factors have been identified in the South African literature as important contextual conditions that influence the teaching and learning of mathematics.

Studies of Singapore’s education system describe sustained teacher professional development, comparatively smaller class sizes, and strong instructional leadership as important features of its mathematics education system ( Choy & Dindyal, 2024; Low & Wong, 2021). The literature further describes the Concrete–Pictorial–Abstract (CPA) approach as a structured instructional strategy that supports learners’ progression from concrete representations to increasingly abstract mathematical reasoning ( Leong et al., 2015; Lutfi & Dasari, 2024). Although these instructional characteristics provide useful comparative insights, they operate within Singapore’s broader educational, cultural, and institutional context and should not be interpreted as direct explanations for differences observed in TIMSS achievement. Furthermore, although South Africa’s curriculum aims for comprehensive coverage, research has identified concerns regarding curriculum overload, with some studies suggesting that limited instructional time may constrain opportunities for learners to consolidate foundational mathematical knowledge ( Milne & Mhlolo, 2021). Collectively, this body of literature provides contextual evidence for interpreting domain-specific achievement patterns rather than establishing causal explanations for the differences observed between South Africa and Singapore.

### 1.4 Curriculum–Cognitive Alignment in International Research

International evidence further illustrates the relationship between curriculum objectives, classroom practices, and mathematics learner achievement. Yılmaz et al. (2021) found that, while mathematics curricula emphasised reasoning, textbook activities placed greater emphasis on application, creating a mismatch between intended and enacted cognitive emphases. Similarly, Bulut and Taşpınar-Şener (2023) reported that the Applying cognitive domain received greater emphasis than the Knowing and Reasoning domains within the mathematics curriculum, although this emphasis varied across grade levels. In primary schools, Pertiwi and Wahidin (2020) showed that fourth-grade assessments placed greater emphasis on number-related content, with comparatively less attention given to geometry and data.

These results align with the TIMSS assessment framework, where Knowing involves factual recall and procedural fluency, Applying requires learners to use mathematical knowledge in familiar contexts, and Reasoning involves non-routine problem-solving and critical thinking ( Mullis et al., 2020; Peduk & Ateş, 2019). International studies have also reported that curriculum alignment across content and cognitive domains is associated with stronger mathematics achievement, although the strength of these relationships varies across educational contexts. Viewed alongside this international evidence, South Africa’s TIMSS 2023 mathematics achievement profile shows comparatively higher average performance in the Applying domain than in the Knowing domain, while substantial achievement differences remain across all three cognitive domains. These patterns are consistent with findings reported in several international studies, although the present study does not examine the classroom or curricular mechanisms underlying these differences. In contrast, Singapore’s balanced performance across the three cognitive domains illustrates the level of cognitive proficiency demonstrated by a high-performing education system assessed on the same TIMSS framework, providing a useful benchmark for interpreting South Africa’s domain-specific achievement profile rather than evidence of direct curricular effects.

### 1.5 TIMSS: A Diagnostic Instrument

TIMSS provides a structured empirical basis for analysing domain-specific performance patterns across participating education systems using a common assessment framework, beyond broad structural explanations. The comparison with Singapore indicates that, although the two education systems are assessed on the same TIMSS Grade 4 mathematics framework, their average achievement profiles differ across both content and cognitive domains. These differences should be interpreted within the broader historical, cultural, socioeconomic, and educational contexts of the two countries rather than as evidence that any single curricular or instructional characteristic explains the observed achievement patterns. The substantially higher average achievement of Singapore’s Grade 4 learners, relative to South Africa’s Grade 5 learners, provides an analytical benchmark for examining domain-specific differences in mathematics achievement. Rather than attributing these differences to learner age or curriculum exposure alone, the comparison highlights the value of examining curriculum coherence, cognitive demands, instructional practices, and teacher development within their respective educational contexts, as documented in previous research.

The TIMSS 2023 results highlight not only the scale of South Africa’s average mathematics achievement relative to the benchmark, but also its domain-specific and cognitive achievement profile. While Singapore provides an example of a high-performing education system characterised in the literature by coherent curriculum design and sustained teacher professional development, South Africa’s mathematics achievement profile indicates comparatively lower average performance in foundational knowledge and Measurement and Geometry, alongside broader systemic challenges identified in the South African literature. Accordingly, the comparison supports context-sensitive reflection on curriculum and instructional priorities rather than direct adoption of another education system’s practices.

### 1.6 Conceptual Framework

This study is guided primarily by the TIMSS conceptual model, which distinguishes between mathematical content domains (Number, Measurement and Geometry, and Data) and cognitive domains (Knowing, Applying, and Reasoning) ( Mullis et al., 2020). Together, these dimensions provide a diagnostic lens for examining not only what learners are expected to know but also how they engage with mathematical tasks at increasing levels of cognitive demand. Rather than relying solely on overall average achievement, the framework enables the examination of domain-specific performance patterns across content and cognitive domains. Within this study, the TIMSS framework functions as the primary analytical structure, guiding the disaggregation of achievement results and the identification of domain-specific achievement gaps.

The study further employs Curriculum Alignment Theory, which emphasises the coherence between curricular intentions, classroom enactment, and assessment demands ( Porter, 2002). Curriculum Alignment Theory proposes that meaningful learning is more likely when the intended curriculum, the implemented curriculum, and the attained curriculum operate in concert. In this study, Curriculum Alignment Theory serves as the principal interpretive lens, supporting the interpretation of domain-specific achievement patterns identified through the TIMSS framework. The theory therefore provides a conceptual basis for examining how alignment between curriculum, instruction, and assessment is reflected in the existing literature, rather than for establishing causal explanations for the observed achievement differences.

International evidence from high-performing education systems such as Singapore describes strong curriculum alignment through a spiral curriculum that revisits mathematical concepts at progressively higher levels of complexity, supported by the Concrete–Pictorial–Abstract (CPA) approach, which scaffolds learning from concrete representations to abstract reasoning ( Leong et al., 2015; Lutfi & Dasari, 2024). In contrast, research on South African primary mathematics identifies an overloaded curriculum, limited instructional time for mastery of foundational domains, and persistent challenges in geometry and spatial reasoning, together with variation in teacher content knowledge and pedagogical confidence ( Maqoqa, 2024; Taylor, 2021). Viewed through the combined TIMSS and Curriculum Alignment frameworks, the comparison between South Africa’s Grade 5 and Singapore’s Grade 4 results provides a structured basis for interpreting differences in domain-specific mathematics achievement within a common international assessment framework.

To maintain conceptual clarity and parsimony, the study does not introduce additional theoretical models beyond these two complementary frameworks. The TIMSS model structures the measurement and domain disaggregation, while Curriculum Alignment Theory guides the interpretation of domain-specific achievement patterns. This dual framework ensures both diagnostic precision and interpretive coherence without theoretical fragmentation.

**Figure 1.**
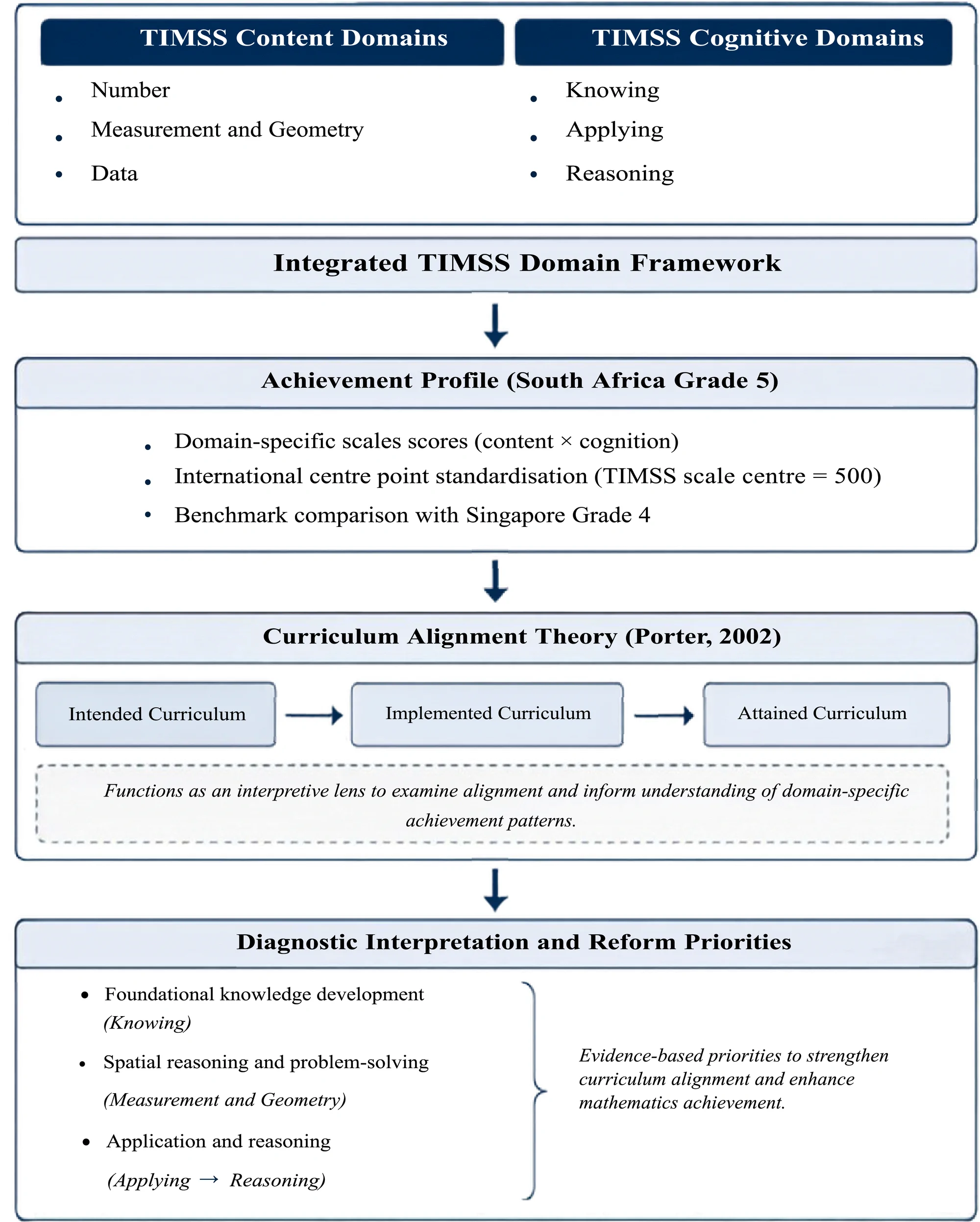
Conceptual framework integrating the TIMSS domain structure and Curriculum Alignment Theory guiding the diagnostic and interpretive analysis.

Figure 1 illustrates the integrated conceptual framework underpinning this study. The TIMSS framework structures the analysis of content and cognitive domains, while Curriculum Alignment Theory guides the interpretation of the resulting achievement patterns. Together, these complementary frameworks provide a diagnostic and interpretive basis for examining South Africa’s Grade 5 mathematics achievement in relation to the TIMSS 2023 assessment framework.

## 2. Methodology

### 2.1 Research Design

This study employed a quantitative secondary data analysis design, using data from the Trends in International Mathematics and Science Study (TIMSS) 2023. This design is particularly appropriate for investigating the mathematics achievement of South African Grade 5 learners across content and cognitive domains for several reasons. First, TIMSS provides a large, internationally standardised dataset that is both rigorous in design and nationally representative, making it suitable for examining learners’ performance patterns with a high degree of reliability. Second, secondary analysis enables the use of TIMSS’s robust psychometric procedures, including item response theory scaling and plausible value methodology, which strengthen the validity of inferences about learners’ achievement. Third, the TIMSS framework allows for meaningful international benchmarking, making it possible to situate South Africa’s performance in relation to high-performing education systems such as Singapore.

Guided by the TIMSS assessment framework, the analysis focused on three content domains (Number, Measurement and Geometry, and Data) and three cognitive domains (Knowing, Applying, and Reasoning). This two-fold focus enabled a diagnostic assessment of learners’ domain-specific achievement profiles within the South African context and supported structured benchmarking against an international reference system. Importantly, the South African sample reported here reflects the TIMSS 2023 Grade 5 administration using the Grade 4 mathematics assessment instruments and framework. Within TIMSS procedures, some education systems assess an adjacent grade when curriculum alignment indicates that the benchmark framework better reflects learners’ opportunity to learn. Consequently, the comparison undertaken in this study is not between different grade curricula, but between two cohorts assessed on the same TIMSS Grade 4 mathematics framework and common international reporting scale. The analysis therefore benchmarks Grade 5 in South Africa against Grade 4 in Singapore on an equivalent instrument, enabling comparison of performance patterns across TIMSS content and cognitive domains on the same scale metric ( Mullis et al., 2020; von Davier et al., 2024). It is important to emphasise that this design is descriptive and comparative rather than causal. The study identifies structured achievement gaps across domains but does not infer grade-level learning gains or causal mechanisms.

### 2.2 Participants

The South African TIMSS 2023 primary grade sample comprised 10,424 Grade 5 learners from 285 schools who were assessed using the TIMSS Grade 4 mathematics instruments, while Singapore assessed 6,530 Grade 4 learners from 181 schools using the same Grade 4 mathematics assessment framework ( Department of Basic Education, 2024; von Davier et al., 2024). The International Association for the Evaluation of Educational Achievement (IEA), in collaboration with Statistics Canada, used a two-stage stratified cluster sampling methodology to guarantee nationally representative estimates ( Siegel & Foy, 2024). In the first stage, schools were selected with probabilities proportional to their size, and in the second stage, intact Grade 5 classes were sampled. The stratification variables included the school sector (public or private), the language of instruction, the geographic region, socioeconomic indicators, the degree of urbanisation, and prior academic achievements (Ibid.). The use of stratified cluster sampling and population weights ensures that reported statistics represent national achievement distributions rather than sample-level estimates.

### 2.3 Data Collection and Analysis

Data for this study were drawn from the TIMSS 2023 mathematics assessment and associated contextual background questionnaires administered to participating learners, teachers, and schools. The TIMSS mathematics achievement is reported on an internationally standardised scale with a centre point of 500 and a standard deviation of 100, enabling valid comparisons across countries and education systems. The mathematics assessment comprised 183 items distributed across three content domains, namely Number (94 items), Measurement and Geometry (49 items), and Data (40 items), as well as three cognitive domains, namely Knowing (58 items), Applying (85 items), and Reasoning (40 items) ( Reynolds, 2024). Each learner completed one assessment booklet, with achievement estimates derived using item response theory and reported as plausible values. Plausible values represent multiple imputed proficiency estimates for each learner and are designed to produce unbiased population-level statistics rather than individual scores ( von Davier, 2020). This design supports reliable population-level estimation while accounting for measurement uncertainty inherent in large-scale assessment data.

The analysis focused on South Africa’s Grade 5 results, and Singapore’s Grade 4 performance was used as an international benchmark to contextualise domain-specific patterns of mathematics achievement. This comparison is consistent with TIMSS procedures, as both cohorts were assessed using the same Grade 4 mathematics framework and instruments, calibrated on a common international scale. Weighted descriptive statistics were computed in accordance with International Association for the Evaluation of Educational Achievement (IEA) guidelines to account for TIMSS’s two-stage stratified cluster sampling design. Sampling weights were applied to ensure nationally representative estimates and to correct for unequal probabilities of selection and non-response, thereby reducing bias in cross-national comparisons ( Siegel & Foy, 2024). All analyses incorporated the full set of plausible values, and estimates were combined using Rubin’s rules in accordance with TIMSS technical procedures to ensure accurate estimation of means and variability. In addition to significance testing, effect sizes (Cohen’s *d*) were calculated to evaluate the practical magnitude of observed differences between domains and between South Africa and Singapore. Effect sizes above 2.5 indicate extremely large educational disparities, reflecting substantial differences in performance between the two systems. Variance estimation incorporated the TIMSS replicate weights to account for the two-stage stratified cluster sampling design. Standard errors and confidence intervals were computed using these replicate weights, ensuring appropriate estimation of sampling variability. The combined use of statistical significance and effect size estimation enabled a more substantively meaningful interpretation of performance gaps, beyond reliance on mean differences alone.

Although TIMSS 2023 collects extensive contextual information through learner, teacher, school, and curriculum questionnaires, the present study prioritised a structured diagnostic comparison of domain-specific achievement profiles. Consequently, contextual variables such as socioeconomic status, language of instruction, school resources, and teacher characteristics were not incorporated into multivariate or multilevel models in the main analysis. The exclusion of these variables from inferential modelling was deliberate, given that the primary objective was to identify and quantify achievement gaps across content and cognitive domains rather than to estimate explanatory or causal effects. Instead, contextual factors are referenced interpretively in the discussion to situate the observed performance patterns within broader structural and policy contexts.

This analytic decision reflects a staged research logic: descriptive domain disaggregation precedes explanatory modelling. Such an approach is consistent with established practice in large-scale assessment research, where diagnostic profiling and causal modelling are treated as sequential and complementary phases of inquiry rather than simultaneous analytical requirements ( OECD, 2019; Rutkowski & Delandshere, 2016).

To ensure appropriate estimation of statistical uncertainty, all achievement comparisons were based on the full set of TIMSS plausible values. Estimates were combined using Rubin’s rules, as prescribed in IEA technical documentation, to account for measurement imputation variability inherent in plausible value methodology. Variance estimation was conducted in accordance with IEA technical guidelines for complex survey data. Weighted means were calculated using student sampling weights, and standard errors were derived using the TIMSS replicate weights to account for the two-stage stratified cluster sampling design.

Statistical inference was undertaken using these replicate-based variance estimates. Effect sizes (Cohen’s *d*) were reported alongside significance tests to provide an educationally meaningful interpretation of observed differences beyond statistical significance alone. Confidence intervals are reported in the supplementary materials to enhance transparency and facilitate replication of domain-level comparisons.

### 2.4 Ethical Considerations

This study used publicly available TIMSS 2023 datasets provided by the IEA. The data contain no personal identifiers and were collected under strict international ethical protocols during the original administration. Because this research involved secondary analysis of anonymised data, no institutional ethics approval was required. There was no formal request to use the dataset from the IEA since the data is available in the public domain, and all analyses adhered to its guidelines for responsible data use.

## 3. Results

This section presents the empirical results addressing Research Questions 1 and 2. Research Question 1 examines South Africa’s achievement profile across TIMSS mathematics content domains, while Research Question 2 examines achievement across cognitive domains. For the purposes of this study, an “achievement gap” is operationally defined as (a) the scale score distance from the TIMSS international centre point of 500, and (b) the benchmark difference between South African Grade 5 learners and Singaporean Grade 4 learners assessed on the same TIMSS Grade 4 mathematics framework. Effect sizes (Cohen’s *d*) are used to indicate the magnitude of these disparities.

### 3.1 Content Domain Achievement

In response to Research Question 1, the analysis reveals that South African Grade 5 learners scored substantially lower average mathematics achievement than Singaporean Grade 4 learners across all three TIMSS content domains: Number, Measurement and Geometry, and Data. Relative to the TIMSS international centre point (500), South Africa’s average achievement in each content domain remained well below the international reference point. These results indicate consistently lower average achievement across all three content domains rather than isolated weaknesses in a single area. Among the three domains, Measurement and Geometry recorded the largest benchmark gap, with the greatest scale score difference between South Africa and Singapore and very large effect sizes (d = 2.66), indicating an extremely large practical difference.  Table 1 presents the weighted mean scores and effect sizes for South African Grade 5 learners and Singaporean Grade 4 learners across the three TIMSS mathematics content domains.

**Table 1.** Mean Scale Scores and Effect Sizes in Mathematics Content Domains, TIMSS 2023.

Content domain	Singapore (Grade 4)	South Africa (Grade 5)	Gap	Cohen’s *d*
Numbers	613	362	251	2.51
Measurement & Geometry	619	353	266	2.66
Data	616	362	254	2.54

Note. All estimates are weighted. Standard errors and confidence intervals were computed using TIMSS plausible values and replicate weights and are reported in the supplementary materials.

All three content domains produced significant large effect sizes (d > 2.5), demonstrating substantial benchmark differences between South African and Singaporean learners. Although Measurement and Geometry recorded the largest observed benchmark gap, all three domains showed pronounced differences in average achievement. Within the South African achievement profile, Measurement and Geometry recorded the lowest average score, whereas Number and Data recorded slightly higher, but still substantially lower, average achievement relative to the Singapore benchmark.

### 3.2 Cognitive Domain Achievement

Addressing Research Question 2, the results indicate that South African Grade 5 learners scored substantially lower average mathematics achievement than Singaporean Grade 4 learners across all three TIMSS cognitive domains: Knowing, Applying, and Reasoning. Among the three cognitive domains, Knowing recorded the largest benchmark gap, with a scale score difference of 267 points and the largest effect size (d = 2.67). Applying and Reasoning also recorded substantial benchmark differences, with effect sizes of 2.49 and 2.46, respectively.  Table 2 presents the weighted mean scores and effect sizes for South African Grade 5 learners and Singaporean Grade 4 learners across the TIMSS mathematics cognitive domains.

**Table 2.** Mean Scale Scores and Effect Sizes in Mathematics Cognitive Domains, TIMSS 2023.

Cognitive domain	Singapore (Grade 4)	South Africa (Grade 5)	Gap	Cohen’s *d*
Knowing	624	357	267	2.67
Applying	615	366	249	2.49
Reasoning	609	363	246	2.46

Note. All estimates are weighted. Standard errors and confidence intervals were computed using TIMSS plausible values and replicate weights and are reported in the supplementary materials.

Within the South African achievement profile, Applying recorded the highest average score (366), followed by Reasoning (363) and Knowing (357). Despite these relative differences, average achievement across all three cognitive domains remained substantially below both the TIMSS international centre point and the Singapore benchmark. Although Applying recorded the highest average achievement within the South African profile, the benchmark differences remained substantial across all cognitive domains **.** The Knowing domain recorded the largest observed benchmark gap, while Applying and Reasoning also demonstrated pronounced differences relative to Singapore.

### 3.3 Item-Level Illustrations

To further illustrate the domain-specific patterns identified in Research Questions 1 and 2, selected released TIMSS items are used to demonstrate how content and cognitive differences are reflected at the task level. In the Knowing domain (Number), more than 90% of Singaporean learners correctly answered an item requiring the recall of basic multiplication facts, compared with fewer than 40% of South African learners. This example is consistent with the comparatively larger benchmark gap observed in the Knowing domain. In the Applying domain (Data), routine tasks involving the interpretation of a simple bar chart were answered correctly by a greater proportion of South African learners than items in the Knowing and Reasoning domains, consistent with the relatively higher average score recorded for Applying within the South African achievement profile. In the Reasoning domain (Measurement and Geometry), items requiring multi-step reasoning involving angle relationships were answered correctly by fewer than 20% of South African learners, compared with a majority of Singaporean learners. This example reflects the substantial benchmark difference observed in tasks requiring mathematical reasoning within geometric contexts. Collectively, these released items illustrate how the benchmark differences identified in the scale scores are reflected across tasks representing different mathematics content and cognitive domains. The examples provide illustrative support for the quantitative results by demonstrating variation in learners’ success across tasks with different content and cognitive demands.

### 3.4 Visualising the Gaps

**Figure 2.**
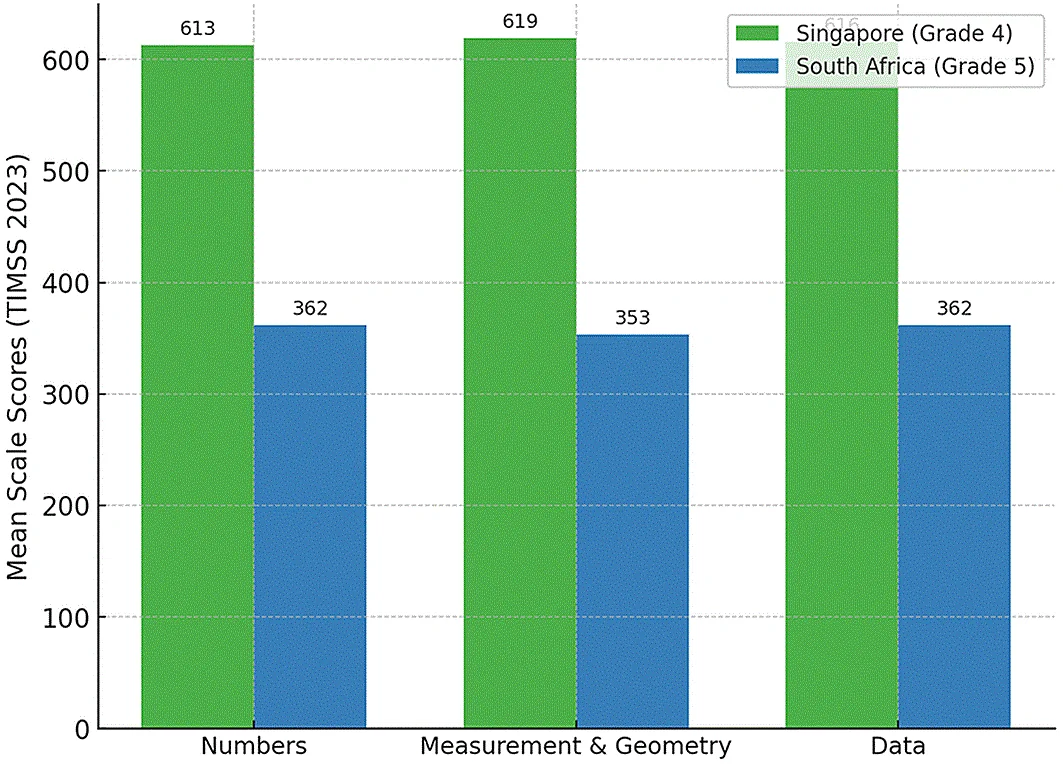
Comparative performance of South African Grade 5 and Singaporean Grade 4 learners across TIMSS content domains (Number, Measurement & Geometry, and Data).

Figure 2 illustrates the comparative performance of South African Grade 5 and Singaporean Grade 4 learners across the three TIMSS mathematics content domains. The figure shows consistently lower average achievement for South African learners across Number, Measurement and Geometry, and Data. Among the three content domains, Measurement and Geometry recorded the largest benchmark gap, although substantial differences were evident across all domains. The graphical presentation complements the results reported in  Table 1 by illustrating the magnitude and distribution of the benchmark differences across the content domains.

**Figure 3.**
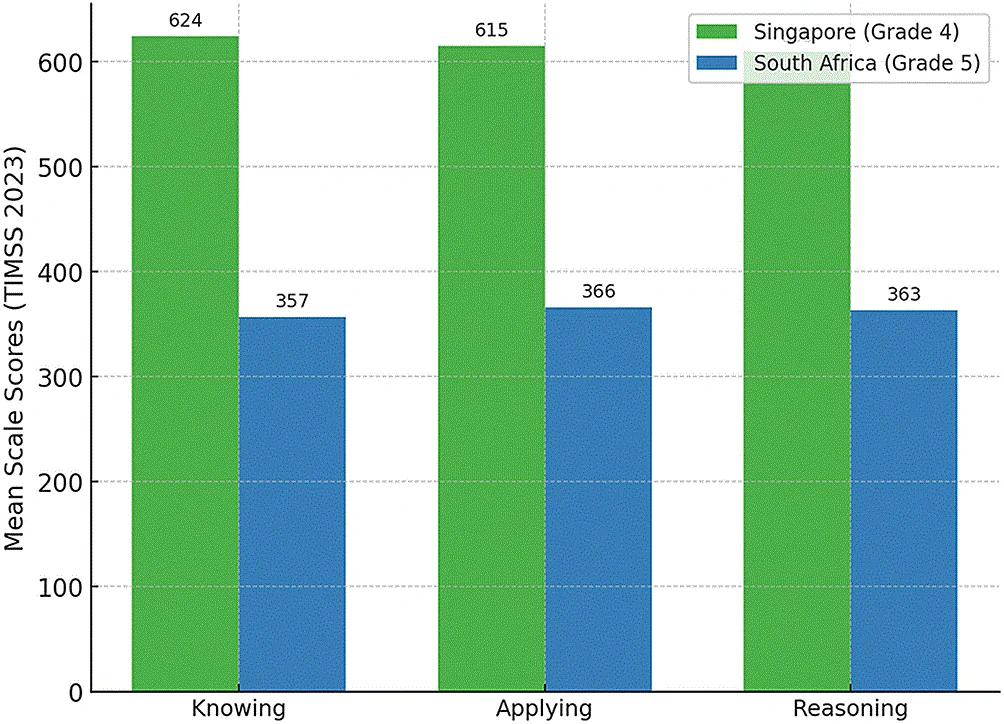
Comparative performance of South African Grade 5 and Singaporean Grade 4 learners across TIMSS mathematics cognitive domains (Knowing, Applying, and Reasoning).

Figure 3 illustrates the comparative performance of South African Grade 5 and Singaporean Grade 4 learners across the three TIMSS cognitive domains. The figure shows that the Knowing domain recorded the largest benchmark gap, while Applying and Reasoning also exhibited substantial benchmark differences. Although Applying recorded the highest average score within the South African cognitive profile, average achievement remained substantially below both the TIMSS international centre point and the Singapore benchmark. Together,  Figures 2 and 3 visually reinforce the quantitative findings presented in  Tables 1 and 2 by illustrating the distribution of benchmark differences across the mathematics content and cognitive domains.

### 3.5 Summary of Results

Synthesising the findings for Research Questions 1 and 2, South African Grade 5 learners recorded substantially lower average mathematics achievement than Singaporean Grade 4 learners across all TIMSS mathematics content and cognitive domains. Among the content domains, Measurement and Geometry recorded the largest benchmark gap, while the Knowing domain recorded the largest benchmark gap among the cognitive domains. Although Applying recorded the highest average score within the South African cognitive profile, substantial benchmark differences remained evident across all three cognitive domains. Overall, the results demonstrate that benchmark differences were observed across all mathematics content and cognitive domains, with comparatively larger differences in Measurement and Geometry and Knowing.

## 4. Discussion

This section interprets the domain-specific and cognitive achievement patterns identified in  Section 3 in relation to curriculum alignment and learning progression. The TIMSS 2023 results indicate that South African Grade 5 learners achieved an average score of 362, substantially below the international centre point of 500. However, the analytical emphasis of this study lies not only in the overall average score, but in the concentration and distribution of achievement gaps across specific content and cognitive domains. Disaggregating achievement reveals that the largest content-domain gap occurs in Measurement and Geometry, while the largest cognitive-domain gap occurs in Knowing. Although substantial achievement differences were observed across all content and cognitive domains, these two domains exhibited the largest benchmark gaps. These domain-specific disparities provide a more precise diagnostic basis for interpreting patterns of mathematics learning and progression in the primary phase.

### 4.1 Content-Domain Patterns and Foundational Gaps

The largest benchmark gap was observed in Measurement and Geometry, where effect sizes indicated an extremely large disparity relative to Singapore. Although Measurement and Geometry exhibited the largest benchmark gap, substantial differences were evident across all three content domains. This finding indicates that the South African mathematics achievement profile reflects broad domain-specific disparities rather than weakness confined to a single content area. While performance in Number and Data also remains substantially below international benchmark levels, the relative ordering of domain means indicates that achievement varies across content areas. The comparatively larger gap in Measurement and Geometry aligns with national research identifying geometry as a persistent area of difficulty in South African classrooms, where studies have reported variation in teacher content knowledge, pedagogical content knowledge, and opportunities for conceptual engagement ( Maqoqa, 2024; Taylor, 2021).

International studies have also associated the development of spatial reasoning with coherent instructional progression and sustained conceptual engagement ( Choy & Dindyal, 2024). Viewed alongside this body of literature, the TIMSS findings suggest that Measurement and Geometry remains an important area for further curriculum and instructional attention in South Africa. Importantly, these results do not suggest an absence of geometry instruction but rather highlight patterns that are consistent with possible differences between intended curriculum, classroom enactment, and assessment expectations. Consistent with Curriculum Alignment Theory ( Porter, 2002), differences between these dimensions may be reflected in learners’ achievement profiles across content domains. The present study, however, does not directly examine curriculum implementation or classroom practice. Consequently, the comparatively larger disparity observed in Measurement and Geometry should be interpreted as a diagnostic indicator within the broader mathematics achievement profile rather than as evidence of a single underlying cause. Together with the substantial differences observed in Number and Data, these findings highlight the importance of strengthening learners’ mathematical development across all content domains while recognising the comparatively greater challenge presented by Measurement and Geometry.

### 4.2 Cognitive-Domain Performance and Learning Progression

The largest cognitive achievement gap was observed in the Knowing domain. South African learners scored substantially lower than Singaporean learners (357 versus 624), with a very large effect size. Although achievement differences were evident across all three cognitive domains, the Knowing domain exhibited the largest benchmark gap. Because the Knowing domain captures factual recall and procedural fluency ( Mullis et al., 2020), the comparatively lower performance in this domain indicates that foundational mathematical knowledge warrants particular attention within South Africa’s overall achievement profile. Within the TIMSS framework, foundational fluency provides the basis for progression to Applying and Reasoning tasks. Viewed alongside this framework, the comparatively lower achievement in Knowing is consistent with the broader pattern of performance observed across the cognitive domains. Although Applying represents a relatively stronger domain within the South African distribution, it remains substantially below international benchmark levels. This pattern indicates variation in achievement across the cognitive domains rather than uniformly low performance.

Previous research has associated secure foundational knowledge with learners’ ability to engage successfully in increasingly complex mathematical tasks ( Mullis & Martin, 2017). Consistent with this literature, the present findings suggest that strengthening foundational mathematical knowledge may support learners’ progression across higher cognitive demands. However, the present study does not directly examine the instructional or curricular processes through which such progression occurs. Similar patterns have been reported in large-scale assessment studies, where learners demonstrate partial procedural competence but experience greater difficulty with tasks requiring conceptual integration and multi-step reasoning. Taken together, these findings describe a cognitive achievement profile characterised by comparatively lower performance in foundational knowledge alongside substantial differences across all three cognitive domains. Rather than identifying causal mechanisms, the results provide a diagnostic profile that may inform future research and curriculum development aimed at strengthening mathematical learning progression.

### 4.3 Curriculum Alignment and Cross-National Insights

The benchmark comparison with Singapore provides a useful context for interpreting domain-specific achievement patterns through the lens of Curriculum Alignment Theory. The literature describes Singapore’s mathematics curriculum as characterised by a tightly sequenced spiral structure and the systematic use of the Concrete–Pictorial–Abstract (CPA) progression, which supports continuity in learners’ mathematical development across content and cognitive domains ( Leong et al., 2015). Within the Singaporean context, these curriculum features have been associated with coherent learning progression and balanced achievement across mathematical domains. Conversely, research on South African primary mathematics has identified concerns regarding curriculum breadth, limited instructional time for consolidating foundational concepts, and persistent challenges in domains such as Measurement and Geometry ( Maqoqa, 2024; Taylor, 2021). These contextual factors have been discussed in the literature as potential influences on mathematics teaching and learning; however, the present study does not directly examine their effects on learner achievement.

Curriculum Alignment Theory ( Porter, 2002) proposes that meaningful learning is more likely when the intended, implemented, and assessed curricula are coherently aligned. Viewed through this theoretical perspective, the comparatively larger achievement gaps observed in Knowing and Measurement and Geometry are consistent with possible differences in curriculum alignment across these dimensions. However, because the present study analyses secondary TIMSS data, it cannot determine the extent to which curriculum alignment or classroom implementation contributed to the observed achievement patterns. Importantly, the comparison should not be interpreted as evidence that Singapore’s curriculum or instructional approaches directly account for its higher achievement. Rather, Singapore provides an analytical benchmark against which South Africa’s domain-specific achievement profile can be interpreted within a common international assessment framework. Accordingly, the comparison offers comparative insight into achievement patterns while recognising the distinct historical, cultural, socioeconomic, and educational contexts within which the two education systems operate.

### 4.4 Interpreting Singapore as a Benchmark

While Singapore’s performance provides a valuable benchmark for examining curriculum coherence and learning progression, it is essential to recognise the contextual conditions within which this performance is situated. Singapore’s education system operates within a highly structured and competitive environment, characterised by strong central curriculum control, selective teacher recruitment, intensive professional preparation, and high societal expectations regarding academic achievement ( Ng, 2017). The literature associates these systemic characteristics with consistent instructional practices and sustained learner engagement, alongside high levels of mathematics achievement across content and cognitive domains. At the same time, these features are accompanied by early differentiation and sustained academic pressure, which, although associated with high achievement, may also generate stress and equity concerns that are not fully captured in large-scale assessment data ( Tan, 2018). This broader context highlights the importance of interpreting benchmark comparisons within their socio-educational settings, rather than attributing performance differences solely to instructional or curricular factors.

The value of Singapore as a comparator lies in providing a well-established international reference point against which South Africa’s achievement profile can be interpreted, rather than serving as a model for direct policy transfer ( Deng, 2013). Within the context of this study, Singapore functions as an analytical benchmark that helps contextualise the domain-specific achievement patterns observed in South Africa. For South Africa, the findings highlight the importance of considering curriculum coherence, focused content progression, and pedagogical scaffolding in ways that are responsive to the country’s educational context, systemic capacity, linguistic diversity, and resource constraints ( Spaull & Kotze, 2015). Accordingly, the comparison supports context-sensitive reflection on mathematics curriculum and instructional priorities rather than wholesale adoption of practices from another education system. The benchmark therefore functions as a comparative reference point that helps to situate the scale and distribution of domain-specific disparities, while recognising the distinct educational contexts in which the two systems operate.

### 4.5 Implications for Policy and Practice

The results identify a dual pattern in South African learners’ mathematics achievement. First, substantial achievement differences are evident across all content and cognitive domains, with comparatively larger gaps observed in foundational knowledge and Measurement and Geometry **.** Second, the relatively stronger performance in the Applying domain indicates comparatively higher average achievement within the South African profile, although performance remains substantially below the international benchmark. Together, these findings indicate variation in achievement across domains rather than uniform mathematics underperformance. From a Curriculum Alignment perspective, these domain-specific patterns are consistent with the possibility of differences between curriculum expectations, instructional practices, and assessment demands, as discussed in the literature. The present study, however, does not directly examine curriculum implementation or classroom practice and therefore cannot determine the extent to which these factors contributed to the observed achievement patterns. Within this context, the findings highlight the importance of strengthening curriculum coherence, instructional sequencing, and opportunities for developing both foundational knowledge and conceptual understanding.

The findings also suggest that efforts to improve mathematics achievement should extend across all content and cognitive domains, while recognising the comparatively larger disparities identified in Measurement and Geometry and the Knowing domain. Accordingly, the results support a balanced approach to curriculum and instructional improvement rather than prioritising a single domain in isolation. More broadly, South Africa has made important progress in expanding access to education and improving average mathematics achievement over successive TIMSS cycles. Nevertheless, substantial inequalities associated with socioeconomic conditions, language, school resources, and educational opportunities continue to shape learners’ mathematics achievement. In addition, the educational disruption associated with the COVID-19 pandemic may have influenced learning opportunities for the cohort assessed in TIMSS 2023 and should therefore be considered when interpreting these findings.

Taken together, these findings indicate that South African mathematics achievement is characterised by substantial domain-specific variation rather than uniformly low performance. The diagnostic achievement profile presented in this study provides evidence that may inform curriculum review, teacher professional development, and future research aimed at strengthening mathematics learning in the primary phase. Interpreted through Curriculum Alignment Theory, these patterns are consistent with possible differences between intended curriculum, classroom enactment, and assessment expectations. Rather than identifying causal mechanisms, the study provides a structured analytical basis for understanding how achievement varies across content and cognitive domains within the TIMSS 2023 assessment framework.

## 5. Conclusion

This study examined domain-specific mathematics achievement among South African Grade 5 learners using TIMSS 2023 data by comparing performance across mathematics content and cognitive domains with Singapore as an analytical benchmark. By disaggregating achievement across these domains, the analysis demonstrated that substantial benchmark differences were evident across all mathematics content and cognitive domains, with comparatively larger differences observed in Measurement and Geometry and the Knowing domain. Rather than relying solely on overall mathematics scores, the study provides a structured diagnostic profile that identifies how achievement varies across specific areas of mathematical knowledge and cognitive demand.

The findings contribute to the literature by demonstrating the value of domain-specific analyses for understanding patterns of mathematics achievement within an internationally comparable assessment framework. Interpreted through Curriculum Alignment Theory, the results are consistent with the importance of coherence between curriculum expectations, instructional practice, and assessment in supporting mathematics learning. However, the study does not identify the underlying causes of the observed benchmark differences. Instead, it provides an empirical basis for informing curriculum review, teacher professional development, and future research aimed at strengthening mathematics learning within the South African context. By positioning Singapore as an analytical benchmark rather than a model for direct policy transfer, the study contributes context-sensitive evidence to comparative mathematics education research and highlights the value of domain-level analyses for informing educational improvement.

## 6. Limitations and Future Research

This study provides a structured diagnostic profile of domain-specific mathematics achievement using TIMSS 2023 data; however, several limitations should be acknowledged. First, the analysis is based on cross-sectional secondary data and therefore supports descriptive and comparative interpretation rather than causal inference. The benchmark differences reported in this study describe patterns of achievement between South African Grade 5 learners and Singaporean Grade 4 learners assessed using the common TIMSS Grade 4 mathematics framework, but they do not explain the processes that produced these differences.

Second, the mathematics content and cognitive domain subscales contain fewer assessment items than the overall mathematics scale, which may reduce the precision of domain-level estimates. Although the use of plausible values and replicate-based variance estimation enhances the robustness of the analyses, statistical uncertainty remains inherent in domain-level comparisons and should be considered when interpreting the reported effect sizes.

Third, TIMSS is designed to measure learner achievement rather than classroom processes or curriculum implementation. Consequently, the study cannot directly examine instructional practices, curriculum enactment, teacher quality, learner engagement, or other contextual factors that may be associated with domain-specific achievement patterns. Interpretations informed by Curriculum Alignment Theory therefore remain theoretical rather than empirically tested within the present study.

Finally, although TIMSS employs nationally representative sampling procedures, certain educational contexts may remain under-represented, particularly small, remote, or multigrade schools. In addition, differences in national exclusion rates should be considered when interpreting benchmark comparisons, as the assessed populations in South Africa and Singapore may not be directly equivalent. Furthermore, the comparison with Singapore should be interpreted as an analytical benchmark within a common international assessment framework rather than as evidence supporting direct policy transfer between education systems.

Future research would benefit from longitudinal, multilevel, and mixed-methods approaches that combine large-scale assessment data with classroom observations, curriculum analyses, teacher data, and learner experiences to examine how domain-specific achievement patterns develop over time and how they may be addressed through contextually appropriate instructional and curriculum interventions.

## Author Contributions

The author, Mathelela Steyn Mokgwathi, is responsible for the conceptualisation, data curation, formal analysis, investigation, methodology, project administration, resources, software, supervision, validation, visualisation, and writing of the original draft, as well as the writing, review, and editing processes.

## Declaration of AI Use

The author affirms that no generative artificial intelligence tools (such as ChatGPT or similar models) were used to produce the academic content, analysis, or interpretations presented in this manuscript. QuillBot (premium) was employed solely for grammar and spelling checks. The author personally reviewed and edited the final manuscript and takes full responsibility for its content and conclusions.

## Data Availability

The data that support the results of this article are derived from the publicly accessible Trends in International Mathematics and Science Study (TIMSS) 2023 database, available through the International Association for the Evaluation of Educational Achievement (IEA) at https://timssandpirls.bc.edu. Derived and processed data underlying the statistical analyses, including the variables used to generate the tables, figures, and descriptive statistics presented in this article, are openly available in the Zenodo repository ( Mokgwathi, 2025) at https://doi.org/10.5281/zenodo.17379412, under the Creative Commons Attribution 4.0 International (CC BY 4.0) licence. These files include anonymised values underlying the means, standard deviations, and effect sizes, as well as detailed variable descriptions and methodological notes supporting replication of the comparative analyses between South Africa (Grade 5) and Singapore (Grade 4). No ethical approval or participant consent was required, as all data originate from secondary sources that are publicly available through the IEA TIMSS 2023 repository.

### Extended Data

Supplementary materials supporting this article are available in the Zenodo repository at https://doi.org/10.5281/zenodo.17379412 ( Mokgwathi, 2025), under the CC BY 4.0 license. These include:

 TIMSS2023_SGvZA_Derived_Dataset.xlsx (domain-level statistics and effect sizes),

 TIMSS2023_Variable_Descriptions.xlsx (variable definitions and sources), and

 TIMSS2023_Supplementary_Notes.pdf (methodological documentation).
