# Supplementary material for: Closing the Gap in Early Mathematics: Domain and Cognitive Insights from TIMSS 2023 in South Africa and Singapore
Source: F1000Res. 2026 Sep 12;14:1209. Originally published 2025 Nov 5. [Version 4] doi: 10.12688/f1000research.172015.4 (PMC13587798; doi:10.12688/f1000research.172015.4)
Supplement: Peer review history

# Reviewer report: Reviewer response for version 4

**DOI:** 10.5256/f1000research.207186.r520589

**Published:** 2026-09-15

**Authors:** MARGUERITE KHAKASA MIHESO O'CONNOR

**Affiliations:** 1 Kenyatta University, Nairobi, Nairobi County, Kenya

The authors have responded satisfactorily to the suggestions and have made commendable efforts to improve the manuscript. The revisions have enhanced the overall coherence and clarity of the article. While the use of generic terms to explain the gaps identified in the results would have been strengthened by incorporating more empirical evidence, this limitation does not substantially detract from the overall quality and coherence of the revised manuscript. I am therefore satisfied with the authors’ responses and recommend the article for Approval status.

Is the work clearly and accurately presented and does it cite the current literature?

Yes

If applicable, is the statistical analysis and its interpretation appropriate?

Partly

Are all the source data underlying the results available to ensure full reproducibility?

Yes

Is the study design appropriate and is the work technically sound?

Yes

Are the conclusions drawn adequately supported by the results?

Yes

Are sufficient details of methods and analysis provided to allow replication by others?

Partly

Reviewer Expertise:

Mathematics Education and teacher Education

I confirm that I have read this submission and believe that I have an appropriate level of expertise to confirm that it is of an acceptable scientific standard.

# Reviewer report: Reviewer response for version 3

**DOI:** 10.5256/f1000research.196841.r490388

**Published:** 2026-07-01

**Authors:** Jennie Golding

**Affiliations:** 1 University College London, London, England, UK

This paper addresses the very, very important issue of South African mathematics performance at Grade 5, as reflected in TIMSS 2023 data and particularly in comparison with that of Grade 4 in the highest-performing system in TIMSS 2023, Singapore. While aiming high is usually commendable, it would be good to have a rationale for aiming quite so high when, as the author notes, the Singapore education system context is very different from that prevalent in South Africa (Indeed, additionally, several authors suggest the prevailing Confucian ethos adds significantly to that context).

 It is valuable to have analysis of South Africa’s TIMSS 2023 performance by content and by cognitive domain: South Africa does not appear to include that in their national report at   TIMSS 2024.pdf whereas a number of other systems do, and e.g. https://tinyurl.com/yx622ea5 shows performance in Grade 4 content and cognitive domains across a number of different countries. (Note, though, that the centre point and SD of TIMSS scores were those established as mean and SD in the first iteration of TIMSS, and the SD varies each cycle, though the centrepoint remains fixed, and separate from the mean).

 It is good also to have technical aspects of TIMSS assessments explained, though sources should always be quoted.

 However, if performances are to be compared then the differences in testing should also be acknowledged: the TIMSS 2023 International Results at https://www.iea.nl/publications/timss-2023-international-report state that South African learners completed paper-based assessment comprised of TIMSS 2019 trend items in at least one grade (so no curriculum-matched scores ae available, though the same source states that there is a 100% South Africa curriculum match with the relevant TIMSS Grade 4 mathematics assessment framework); and that South Africa assessed students in their fifth and ninth years of schooling to better match curricula and maintain trend measurement (which clarifies the rationale for Grade 5 participation). 

 Similarly, the paper very largely refers to average performance (and including that word ‘average’ each time would strengthen rigour). The national report already identifies significant disparities by gender (with girls significantly outperforming boys, on average) and an average 111-point gap between those who “Always” speak the LOLT at home (405) and those who “Never” do (294), as well as very large gaps in average scores by socio-economic background. Note also that Singapore’s exclusion rate was >> that in South Africa. Taken together, these considerations contribute to a very different distribution of scores in South Africa than the distribution seen among the (non-excluded) assessed pupils in Singapore.

 However, the comparison across the two countries remains fairly simple, focusing on gaps between average scores in each (content and cognitive domain). The patterns seen in South Africa (relatively low scores in Measurement and Geometry, and in Knowing) are not uncommon across education systems: see e.g. https://tinyurl.com/yx622ea5  - and yes, probably at least in part reflect a mismatch between the intended curriculum and the enacted, or received, curriculum. However, in each domain, the difference between focus average performance in South Africa and that in Singapore amounts of 2.5-2.7, using Cohen’s d to 1 dp, which is a very substantial difference of a magnitude broadly comparable across domains.

 In terms of response by that measure, the focus for action needs to be across the curriculum, rather than primarily in just one content and just one cognitive domain (and of course there is a swathe of evidence from across the globe that points to the desirability of learning development across content and cognitive domains).

 Given the starkness of the contrast between reported average scores in South Africa and those in Singapore, it would be useful to report also the statistical significance of the differences across domains *within South Africa* in terms of each of content and cognition, which might give a more realistic steer as to the urgency of a particular focus within mathematics learning.

 The author very rightly points to the necessity of adapting apparently more successful approaches from other contexts, but greater criticality in the use of international literature would further strengthen the paper, e.g. curriculum papers are usually focused on one particular curriculum, set of curriculum materials, classroom practices etc. – so cannot unproblematically be transferred across very different cultural and contextual systems. It’s also important to avoid implying causation without due regard to rigour: for example ‘shaping’ suggests causality and that needs to be justified if it is claimed. Further, all claims such as those around curriculum system responses should be referenced (together with critique of the related evidence in relation to transferability to the South African context).

 Finally, the focus of this paper is the comparison with Singapore, pointing to significant potential for enhancement of learning in South Africa. However, it is also important to acknowledge the enormous progress made in recent years, post-apartheid, to give more equitable access to a broad and balanced mathematics curriculum across young people in South Africa, with the related challenges to resources and in particular, the equipping of teachers in all communities. TIMSS 2023 assessments also took place in the shadow of the global pandemic. The TIMSS 2024.pdf national report recognises the sustained and significant impact of the pandemic on learning across much of South Africa, not least because of very limited digital infrastructure to support continued learning. Learners in Singapore also suffered pandemic impact, as evidenced by the drop in average scores from TIMSS 2019, but evidence from Singapore suggests a pivot to digital or hybrid learning was more widely achieved.

Is the work clearly and accurately presented and does it cite the current literature?

Yes

If applicable, is the statistical analysis and its interpretation appropriate?

Partly

Are all the source data underlying the results available to ensure full reproducibility?

Yes

Is the study design appropriate and is the work technically sound?

Partly

Are the conclusions drawn adequately supported by the results?

Partly

Are sufficient details of methods and analysis provided to allow replication by others?

Yes

Reviewer Expertise:

International large scale studies in mathematics and science; mathematics education policy-practice interface; mathematics teacher education.

I confirm that I have read this submission and believe that I have an appropriate level of expertise to confirm that it is of an acceptable scientific standard, however I have significant reservations, as outlined above.

# Response

**Published:** 2026-07-23

**Authors:** Mathelela Steyn Mokgwathi

**Affiliations:** Early Childhood Education, University of South Africa, Pretoria, Gauteng, South Africa

Dear Dr Golding,

 I would like to sincerely thank you for your comprehensive, thoughtful, and constructive review of this manuscript. Your comments have been invaluable in strengthening the study, particularly regarding its comparative framing, methodological clarity, and interpretation of the findings. I greatly appreciate the time and expertise you devoted to reviewing the manuscript.

 In response to your recommendations, I have substantially strengthened the rationale for benchmarking South African Grade 5 learners against Singaporean Grade 4 learners by explaining the TIMSS procedures that permit assessment of adjacent grades using the common TIMSS Grade 4 mathematics framework. The manuscript now makes it clear that Singapore is used as an analytical benchmark within a common international assessment framework rather than as a model for direct policy transfer. Throughout the manuscript, I have also acknowledged the considerable historical, cultural, socioeconomic, and educational differences between the two countries and emphasised that the findings should be interpreted within their respective contexts.

 The Introduction and Literature Review have been revised to provide a more balanced discussion of South Africa's mathematics achievement, recognising the significant progress made since the advent of democracy while also acknowledging the persistent inequalities associated with socioeconomic conditions, language, school resources, and educational opportunities. I have also incorporated discussion of the potential influence of the COVID-19 pandemic on the TIMSS 2023 cohort to provide a more comprehensive contextual interpretation of the findings.

 The manuscript has been carefully revised to enhance methodological precision and interpretive rigour. References to mathematics performance have been standardised to average mathematics achievement where appropriate, and causal language has been removed or moderated throughout the manuscript. Expressions that could imply causal inference have been replaced with more appropriate terminology that reflects the descriptive and comparative nature of the study. In addition, the Methods section has been expanded to clarify the analytical procedures employed, including the use of plausible values, sampling weights, replicate-based variance estimation, and effect-size estimation in accordance with IEA technical guidelines.

 The Results, Discussion, and Conclusion have also been substantially revised. The Results section now focuses exclusively on descriptive reporting of the empirical findings, while interpretation has been reserved for the Discussion. The manuscript now consistently emphasises that substantial benchmark differences were observed across **all** mathematics content and cognitive domains, with comparatively larger differences in Measurement and Geometry and the Knowing domain, thereby avoiding undue emphasis on a single domain. The Discussion has been strengthened by critically engaging with the international literature and by positioning Curriculum Alignment Theory as an interpretive framework rather than as evidence of causal relationships. The Conclusion has likewise been revised to ensure that all conclusions are directly supported by the reported findings.

 Finally, the Limitations section has been expanded to acknowledge the descriptive nature of the study, the constraints of cross-sectional secondary data, the limitations inherent in domain-level comparisons, differences in national exclusion rates, and the need to interpret the comparison with Singapore as an analytical benchmark rather than evidence supporting direct policy transfer.

 I trust that these revisions have addressed the concerns raised and have strengthened the manuscript in terms of methodological transparency, interpretive balance, and its contribution to comparative mathematics education research.

 Thank you once again for your insightful and constructive review.

 Kind regards,

 Dr Mathelela Steyn Mokgwathi

# Reviewer report: Reviewer response for version 3

**DOI:** 10.5256/f1000research.196841.r472940

**Published:** 2026-04-13

**Authors:** Fidele Ukobizaba

**Affiliations:** 1 University of Rwanda College of Education, Rwamagana, Rwanda; 2 African Centre of Excellence for Innovative Teaching and Learning Mathematics and Science (ACEITLMS), University of Rwanda College of Education, Kayonza, Rwanda

The authors have made notable improvements to the manuscript compared to the previous version. Most of the earlier concerns have been addressed, and the manuscript is now generally readable. However, some issues still require revision:

1. The third research objective is overly lengthy and lacks sufficient focus. Furthermore, it is unclear how this objective is operationalized in the results section, as no corresponding findings are presented. Considering this and given the absence of aligned results, it is recommended that this objective be removed.
2. Following the revision of Comment 1, the statement in the first paragraph of results section: “Research Question 3, concerning curriculum and pedagogical implications, is addressed in the Discussion section” should also be deleted.
3. The discussion section should explicitly interpret and synthesize the study findings in relation to relevant literature. Accordingly, Sections 4.6 and 4.7 should be removed, as they do not directly engage with or interpret the study results.
4. A strong conclusion should sound clear, concise, and synthesis-oriented. It should not introduce new ideas, nor discuss the results, and should not include citations. Instead, it should bring the study to a logical close by highlighting meaning, contribution, and implications.
5. The limitations section appears excessively extensive, which may weaken the perceived robustness of the study. It is recommended that the authors consolidate this section into two concise paragraphs.

Is the work clearly and accurately presented and does it cite the current literature?

Yes

If applicable, is the statistical analysis and its interpretation appropriate?

Yes

Are all the source data underlying the results available to ensure full reproducibility?

Partly

Is the study design appropriate and is the work technically sound?

No

Are the conclusions drawn adequately supported by the results?

No

Are sufficient details of methods and analysis provided to allow replication by others?

No

Reviewer Expertise:

Mathematics education.

I confirm that I have read this submission and believe that I have an appropriate level of expertise to confirm that it is of an acceptable scientific standard, however I have significant reservations, as outlined above.

# Response

**Published:** 2026-04-15

**Authors:** Mathelela Steyn Mokgwathi

**Affiliations:** Early Childhood Education, University of South Africa, Pretoria, Gauteng, South Africa

Dear Dr Ukobizaba,

 I would like to thank you for your constructive feedback and for recognising the improvements made in the manuscript. In this revision, I have carefully addressed the remaining concerns you raised. The third research question has been removed to ensure alignment between the study objectives and the empirical findings, and all references to it in the Results section have been deleted.

 The Discussion section has been substantially revised to strengthen the interpretation and synthesis of findings in relation to relevant literature. Sections 4.6 and 4.7 have been removed, and the discussion now focuses on analytically engaging with the results rather than presenting prescriptive content. In addition, the Conclusion has been rewritten to ensure that it is concise, synthesis-orientated, and free from citations or new arguments.

 Finally, the Limitations section has been consolidated into two concise paragraphs to improve clarity and balance while retaining key methodological considerations.

 I trust that these revisions address the remaining concerns and further strengthen the clarity, coherence, and scholarly contribution of the manuscript.

 Kind regards,

# Reviewer report: Reviewer response for version 2

**DOI:** 10.5256/f1000research.194776.r453643

**Published:** 2026-02-17

**Authors:** Fidele Ukobizaba

**Affiliations:** 1 University of Rwanda College of Education, Rwamagana, Rwanda; 2 African Centre of Excellence for Innovative Teaching and Learning Mathematics and Science (ACEITLMS), University of Rwanda College of Education, Kayonza, Rwanda

The study addresses an important and timely issue, “Closing the gap in early mathematics”, which is commendable. However, the manuscript has several substantial concerns that should be addressed to strengthen its quality and clarity.

 The introduction does not clearly identify or elaborate on the specific gaps in early mathematics among South African students. As a result, the purpose and direction of the study are not sufficiently clear to the reader. The focus and motivation of the study should be made explicit earlier in the manuscript. In this regard, think about the first line of the conclusion (which may be the aim of the study), where it is written: “This study examined South African Grade 5 learners’ mathematics achievement in TIMSS 2023, disaggregated by content and cognitive domains and benchmarked against Singapore’s Grade 4 performance.”

 The research questions are currently too broad and appear to target multiple aspects simultaneously, resulting in a lack of focus. For greater coherence and rigor, the research questions should be more specific and precisely formulated.

 The manuscript draws on three theoretical frameworks. This may dilute the conceptual focus of the study. The authors are encouraged to consider using one framework, or at most two that clearly complement each other, to provide a more coherent theoretical grounding.

 Research design raises concerns. The rationale for comparing Grade 5 South African students with Grade 4 Singaporean students is not adequately justified, as these groups are not at the same educational level. Comparisons should ideally be made between equivalent grade levels. Moreover, comparing two groups’ results alone is insufficient to identify learning gaps in mathematics. The authors should consider an alternative design or provide a stronger justification for their approach.

 The results section primarily reports differences in performance between the two groups (effect size) but does not clearly identify or analyze “gaps” in learning. Given the title, *“Closing the gap in early mathematics…,”* the manuscript should explicitly identify where the gaps exist, describe their nature, explore possible reasons for them, and suggest ways these gaps could be addressed.

 In the discussion section, it is written: “This section addresses Research Question 3 by interpreting domain-specific and cognitive performance patterns in relation to curriculum alignment, pedagogy, and teacher professional development and outlining implications for improving mathematics achievement in South Africa.” However, this section should be discussing the results in line with the research questions. Additionally, this section should more explicitly connect the findings to existing literature and prior research to situate the study within the broader scholarly conversation.

Is the work clearly and accurately presented and does it cite the current literature?

Yes

If applicable, is the statistical analysis and its interpretation appropriate?

Yes

Are all the source data underlying the results available to ensure full reproducibility?

Partly

Is the study design appropriate and is the work technically sound?

No

Are the conclusions drawn adequately supported by the results?

No

Are sufficient details of methods and analysis provided to allow replication by others?

No

Reviewer Expertise:

Mathematics education.

I confirm that I have read this submission and believe that I have an appropriate level of expertise to confirm that it is of an acceptable scientific standard, however I have significant reservations, as outlined above.

# Response

**Published:** 2026-02-19

**Authors:** Mathelela Steyn Mokgwathi

**Affiliations:** Early Childhood Education, University of South Africa, Pretoria, Gauteng, South Africa

**Reviewer Comments**

**Response to Comments**

 The introduction does not clearly identify or elaborate on the specific gaps in early mathematics among South African students. The purpose and direction of the study are not sufficiently clear.

 The introduction has been revised to explicitly state the purpose of the study earlier and to clearly identify the specific achievement gaps motivating the analysis. The revised text now highlights that the largest content-domain gap occurs in Measurement and Geometry and the largest cognitive-domain gap occurs in Knowing. The study aim is explicitly articulated in the opening section, clarifying the diagnostic intent of disaggregating achievement across content and cognitive domains.

 The research questions are too broad and lack focus.

 The research questions were refined for precision and coherence. Research Questions 1 and 2 now focus specifically on content-domain and cognitive-domain profiling, while Research Question 3 is limited to curriculum and pedagogical implications derived from the empirical findings. This ensures a clear alignment between Results (RQ1 and RQ2) and Discussion (RQ3).

 The manuscript draws on three theoretical frameworks, which may dilute conceptual focus.

 The conceptual framework has been streamlined to retain two complementary frameworks: the TIMSS domain framework and Curriculum Alignment Theory (Porter, 2002). Additional theoretical layering was removed to improve conceptual clarity. Figure 1 was redesigned to reflect this streamlined structure.

 The rationale for comparing Grade 5 South African learners with Grade 4 Singaporean learners is not adequately justified. Comparisons should ideally be between equivalent grade levels.

 The methodology section now clearly explains that both cohorts were assessed using the same TIMSS Grade 4 mathematics framework and reporting scale. The comparison is instrument-equivalent rather than curriculum-equivalent. The manuscript explicitly clarifies that the design is descriptive and comparative, not causal, and is intended to benchmark performance patterns rather than infer grade-level learning differences.

 Comparing two groups alone is insufficient to identify learning gaps.

 The Results section now operationalises “achievement gap” explicitly as (a) deviation from the TIMSS international centre point and (b) benchmark scale-score differences with associated effect sizes. The analysis identifies the location, magnitude, and concentration of domain-specific gaps rather than merely reporting mean differences.

 The Results section reports effect sizes but does not clearly analyse gaps in learning.

 The Results section was restructured to explicitly identify where gaps exist, describe their magnitude, and distinguish between uniform underperformance and concentrated domain-specific weaknesses. Item-level illustrations were included to demonstrate how gaps manifest in foundational fluency and spatial reasoning tasks.

 The Discussion should more explicitly connect findings to the research questions and prior literature.

 The Discussion was reorganised to mirror the empirical findings and to explicitly anchor each subsection in the identified content and cognitive gaps. Connections to prior South African and international research were strengthened, and interpretations are framed within the retained theoretical structure.

 Is the study design appropriate and technically sound? (Reviewer indicated “No”)

 The Method section was strengthened by explicitly detailing the use of plausible values, Rubin’s rules for combining estimates, replicate weights for variance estimation, complex sampling adjustments, and effect size interpretation. The manuscript now clearly states that the analysis is descriptive and non-causal.

 Are sufficient details provided to allow replication? (Reviewer indicated “No”)

 Additional methodological transparency was incorporated. The manuscript now specifies estimation procedures, variance computation methods, and reporting of confidence intervals. Derived datasets and methodological documentation have been deposited in an open-access repository to support replication.

 Are the conclusions adequately supported by the results? (Reviewer indicated “No”)

 The Conclusion was revised to align strictly with the empirical findings. Causal overreach was removed, reform priorities are directly grounded in identified domain-specific gaps, and claims are framed proportionately to the descriptive comparative design.

# Response

**Published:** 2026-04-15

**Authors:** Mathelela Steyn Mokgwathi

**Affiliations:** Early Childhood Education, University of South Africa, Pretoria, Gauteng, South Africa

Dear Dr Ukobizaba,

 I would like to thank you for your detailed and constructive feedback, which has significantly strengthened this manuscript. In this revision, I have clarified the research gap in the Introduction by explicitly identifying the limited focus on domain-specific mathematics achievement at the primary level and by stating the purpose of the study more clearly at an earlier stage. The research questions have been refined and reduced to ensure greater focus and alignment with the study design. In addition, the conceptual framework has been streamlined by adopting curriculum alignment theory as the primary analytical lens, with the TIMSS framework providing the structure for domain-level analysis.

 I have also strengthened the justification for comparing South African Grade 5 learners with Singaporean Grade 4 learners by explaining the use of the TIMSS Grade 4 assessment framework and ensuring comparability on a common measurement scale. The Results section has been revised to explicitly identify and analyse achievement gaps across content and cognitive domains, rather than focusing only on differences in performance.

 Furthermore, the Discussion section has been restructured to align directly with the research questions, remove references to the previously included third research question, and strengthen the integration of findings with existing literature. The Methods section has been expanded to provide clearer detail on analytical procedures, including the handling of plausible values, sampling weights, and variance estimation, to enhance transparency and reproducibility. Finally, the conclusion has been rewritten to ensure that it is fully supported by the findings and provides a clear and concise synthesis of the study’s contribution.

 I trust that these revisions address the concerns raised and improve the overall clarity, methodological rigour, and contribution of the study.

 Kind regards,

# Reviewer report: Reviewer response for version 1

**DOI:** 10.5256/f1000research.189692.r435305

**Published:** 2025-12-30

**Authors:** MARGUERITE KHAKASA MIHESO O'CONNOR

**Affiliations:** 1 Kenyatta University, Nairobi, Nairobi County, Kenya

****Review  report

 Introduction

 The article addresses an important and timely question in mathematics education: domain-specific patterns of achievement in TIMSS 2023, comparing South African Grade 5 learners with Singaporean Grade 4 learners. The topic is relevant and well grounded providing an opportunity for policy and practice impact in South Africa and the wider Sub-Saharan region.

 The research purpose and alignment with TIMSS conceptual frameworks are quite clear. Descriptive statistical analysis and accurate identification of domain-specific weaknesses is evident.  There is observed an effective use of TIMSS as an international benchmark to highlight systemic performance gaps. The integration of literature from both high-performing (Singapore) and lower-performing (South Africa) systems is provided in a coherent narrative that links content and cognitive deficits to curriculum and instructional factors.

 However several conceptual and methodological limitations  which currently reduce the manuscript’s rigor. have been identified. Revisions are required, particularly in theoretical framing, TIMSS- specific methodology, and the depth of interpretation and contextualization. Some minor corrections have also been identified for authors attention for proof reading

 The  following observations have been made and related. action points suggested for consideration

 A): Presentation

 The article  is generally well structured, articulate, and contextually grounded. The introduction to the article provides a clear rationale for focusing on South African primary mathematics achievement using TIMSS 2023 and situates the work within persistent national under performance. The performance gap (- 250 points) between South African Grade 5 and Singaporean Grade 4 learners is clearly stated and framed as a systemic issue rather than a purely learner-level deficit. The need to move beyond overall scores to content and cognitive domain analysis is well argued and aligned with TIMSS reporting structures. In addition, the contribution of the study, especially the focus on Grade 5 as an under-researched phase relative to Grade 9, is well articulated

 The following issues were noted in the presentation

 The rationale  of benchmarking South African Grade 5 learners against Singaporean Grade 4 learners though  methodologically common in TIMSS is not well explained. The clarification of why Singapore Grade 4 is used as a comparator is necessary . Secondly ,the article introduces and sustains a misaligned reference to Early Childhood Education (ECE). This introduction at one point frames the work as focusing on * Early Childhood Education,* which is conceptually inaccurate for TIMSS Grade 5. Grade 5 sits in the primary/intermediate phase, not ECE.. In addition, the conceptual rationale for the theoretical framing of content and cognitive domains did not recognize frameworks such as: TIMSS assessment framework, Kilpatrick et al.’s strands of mathematical proficiency and  theories of cognitive demand and learning progression,. Finally, there is need to clarify  the research gap.  It is stated that Grade 5 and domain-specific analyses are under-researched, but this gap is not supported by specific citations and a brief synthesis of existing work.. 

 As a result, the author is encouraged  to Action as follows

- Clearly justify the benchmarking of South Africa Grade 5 against Singapore Grade 4.
- Remove or revise all references to ECE; and  use more accurate terms  
- Strengthen theoretical framing by linking content and cognitive domains to recognized frameworks (e.g., mathematical proficiency, cognitive demand, learning trajectories).
- Clarify the research gap with citations that show the relative neglect of Grade 5 and domain-specific analyses.
- Streamline the introduction by reducing redundant statements and improving paragraph transitions

 B: Study Design and Data Sources.

 The use of quantitative secondary analysis of TIMSS 2023 data is appropriate and well aligned with the research questions. The sample sizes and the national representativeness of the TIMSS samples justifies the generalizability of the findings. The recognition of  TIMSS as a well-established international assessment enables valid cross-national comparisons. The description of the test design is broadly accurate and shows familiarity with large-scale assessment methodology.

  The following issues were observed

 The  description of TIMSS technical procedures in the article is incomplete.  Although TIMSS design is mentioned, the article does not explicitly state how plausible values (PVs) were handled in the analysis. These details will help to confirm that TIMSS technical standards were followed.

*The* analysis does not explain whether or how contextual variables collected during the study (SES, language of instruction, teacher qualifications, opportunity-to-learn indicators) were used. Given TIMSS’s strong emphasis on context, this is a missed opportunity for explanatory depth.

 For the psychometric and reliability considerations, there is need for reference to internal consistency, While TIMSS provides validated items, secondary analyses typically reference psychometric adequacy or refer explicitly to IEA technical documentation. Inclusion of some justification of Grade-Level Comparison is missing *.* A rationale for South Africa testing at Grade 5 (rather than Grade 4) for the primary cycle should be explicitly noted to strengthen the conceptual legitimacy of the comparison with Singapore Grade 4.

 The following action points are suggested

- Ex *plicitly describe* the use of plausible values (number of PVs, which domains, how handled in analysis).
- *Detail the weighting strategy* , including sampling weights and replicate weights, and explain how the two-stage sampling structure was accounted for.
- *Indicate whether contextual variables* (SES, language, school resources, teacher characteristics) were included in any extended analysis; if not, explain this limitation.
- *Reference psychometric evidence* (e.g., IEA technical reports) to reassure readers about reliability and validity of domain scores.
- *Clarify the grade-level choice* for South Africa and how it affects comparability with Singapore Grade 4.

 C: Methodology and Statistical Analysis The categorization of content domains (Number, Measurement & Geometry, Data) and cognitive domains (Knowing, Applying, Reasoning) is appropriate and consistent with TIMSS. The use of weighted estimates, mean differences, and effect sizes (Cohen’s d) is methodologically  suitable for characterizing performance gaps.. 

 The following issues were observed:

 To avoid biased estimates, domain-level comparisons in TIMSS require appropriate PV handling and replication methods. It is not clear whether the analysis uses domain-specific plausible values correctly 

 For the variance estimation and significance testing, it is does not specify whether standard errors, confidence intervals, or p-values were computed. Descriptive statistics without uncertainty estimates weaken inferential robustness. The author had limited use of advanced modelling. The analysis is largely descriptive (means and effect sizes)..Given the richness of TIMSS data, multivariate methods (e.g., regression, multilevel models, mediation with contextual variables, DIF analyses) could substantially deepen the insights. While Cohen’s D  is reported, the interpretation of effect sizes in context is limited. There is little discussion of what constitutes a small/medium/large effect in educational terms., 

 Suggested action points

- Specify analytical tools and procedures used to handle PVs and weights (software, settings, treatment of domains).
- Report standard errors and/or confidence intervals for key estimates and clarify whether hypothesis tests were performed.
- Where feasible, incorporate multivariate or multilevel analyses to examine how contextual variables relate to domain-specific performance.
- Interpret effect sizes in educationally meaningful terms, not just numerically.

D: Interpretation of Findings, Discussion, and Conclusion

 The discussion is comprehensive and analytically rich. Content and cognitive domain findings and linking them to curriculum, pedagogy, and systemic factors is integrated well. Content-level weaknesses  and cognitive weaknesses are clearly interpreted and consistent with previous research .Policy and practice implications including pillars of reform around foundational knowledge, geometry/spatial reasoning, and leveraging application skills to cultivate reasoning is well structured. The conclusion offers a strong synthesis, links to broader goals such as SDG 4, and emphasizes curriculum-pedagogy-assessment coherence through Curriculum Alignment Theory

 Issues observed included  the need for greater engagement with learning progression theory, to deepen the explanatory power.

 Some  claims require evidence or qualification statements such as “procedural accuracy over conceptual exploration” or “curriculum spreads content thinly” need either citation or more cautious phrasing.

 The claim that learners are “stronger in applying” requires quantification and statistical validation .

 ECE References  in this section should also be expunged and corrected .

  To maintain academic balance, the discussion on using  Singapore as a model should acknowledge potential constraints such as high academic pressure, streaming, selective teacher recruitment, and cultural expectations.

 Some weaknesses (e.g., geometry and knowing) are repeated in multiple sections affecting  structure   In Pillar(3) three  repetition  is noted, The discussion is not using empirical evidence and relies of vague quantifiers. The structure of the discussion could be sharpened with clearer subheadings and more concise phrasing.

 Some *recommendations* (e.g., reducing class sizes) may be politically or financially challenging; indicating alternative or incremental strategies would strengthen the practical relevance.

 The conclusion would benefit from a brief statement of study limitations (e.g., reliance on secondary data, limited use of contextual variables, cross-sectional design) and directions for future research.

 Action points suggested

- Deepen theoretical engagement by explicitly drawing on mathematical proficiency and learning progression frameworks.
- Support key claims with evidence (citations or data) and quantify statements about “relative strength” in applying.
- Remove or reframe ECE references, using appropriate phase terminology.
- Provide a more nuanced view of Singapore, noting contextual constraints and non-transferable features.
- Consolidate repeated material on geometry/knowing weaknesses and improve subheadings and paragraph transitions.
- Comment on feasibility of policy recommendations and suggest realistic pathways or phases of implementation.
- Add a short section on study limitations and future research directions
-  replace vague quantifiers with measurable empirical evidence

 Summary of Key Action Points for Revision

*Conceptual and Theoretical*

 Clarify the benchmarking rationale (Singapore Grade 4 vs South Africa Grade 5).

 Correct ECE misalignment and use appropriate phase terminology.

 Strengthen theoretical framing using TIMSS, mathematical proficiency, and learning progression frameworks.

 Provide a more balanced portrayal of Singapore, acknowledging contextual constraints.

*Methodological and Statistical*

 Explicitly describe handling of plausible values, sampling weights, and replicate weights.

 Report standard errors, confidence intervals, and significance tests for key comparisons.

 Consider multivariate or multilevel analyses using contextual variables

 Interpret effect sizes substantively, not just numerically.

*Context and Interpretation*

 Integrate contextual questionnaire data (SES, language, resources, teacher factors) where possible.

 Link performance patterns explicitly to systemic factors (teacher knowledge, curriculum pacing, language of learning and teaching)

 Ensure claims are evidence-based and appropriately referenced.

*Structure and Presentation  >>minor corrections*

 Streamline the introduction and discussion, removing redundancy and improving coherence.

 Strengthen section headings and transitions for easier navigation.

 Include tables or figures with domain-specific means, SEs, and effect sizes

 Add a concise section on limitations and future research.

Is the work clearly and accurately presented and does it cite the current literature?

Yes

If applicable, is the statistical analysis and its interpretation appropriate?

Partly

Are all the source data underlying the results available to ensure full reproducibility?

Yes

Is the study design appropriate and is the work technically sound?

Yes

Are the conclusions drawn adequately supported by the results?

Yes

Are sufficient details of methods and analysis provided to allow replication by others?

Partly

Reviewer Expertise:

Mathematics Education and Teacher Education

I confirm that I have read this submission and believe that I have an appropriate level of expertise to confirm that it is of an acceptable scientific standard, however I have significant reservations, as outlined above.

# Response

**Published:** 2026-01-01

**Authors:** Mathelela Steyn Mokgwathi

**Affiliations:** Early Childhood Education, University of South Africa, Pretoria, Gauteng, South Africa

I would like to thank Reviewer 2 for the detailed and constructive feedback concerning theoretical framing, TIMSS-specific methodology, interpretation of domain-level findings, and the balance and structure of the Discussion and Conclusion. These concerns have been addressed through strengthened integration of mathematical proficiency and learning progression frameworks, expanded methodological transparency regarding plausible values and variance estimation, refined interpretation of effect sizes, removal of misaligned ECE references, and a more nuanced treatment of Singapore as a benchmark. We believe the revisions comprehensively address the issues identified.

# Response

**Published:** 2026-04-15

**Authors:** Mathelela Steyn Mokgwathi

**Affiliations:** Early Childhood Education, University of South Africa, Pretoria, Gauteng, South Africa

Dear Dr O’Connor,

 I would like to thank you for your detailed and constructive feedback, which has contributed significantly to strengthening this manuscript. In this revision, I have clarified the benchmarking rationale by providing a detailed explanation of the use of the TIMSS Grade 4 framework and ensuring comparability between South African Grade 5 and Singaporean Grade 4 learners. I have also strengthened the articulation of the research gap, removed misaligned references to Early Childhood Education, and refined the theoretical framing to improve conceptual clarity.

 In addition, I have expanded the methodological section to provide clearer explanations of TIMSS procedures, including the handling of plausible values, sampling weights, and variance estimation, and clarified the descriptive and diagnostic focus of the study in relation to the use of contextual variables and modelling approaches. The interpretation of effect sizes has been strengthened, and claims have been revised to ensure they are appropriately supported and cautiously framed.

 The discussion has been revised to enhance analytical depth, reduce prescriptive elements, improve structural coherence, and better integrate the findings with existing literature, while also providing a more balanced interpretation of the Singapore comparison. A concise limitations section has also been included to reflect key design and contextual constraints.

 I trust that these revisions address the concerns raised and improve the overall clarity, rigour, and contribution of the study.

 Kind regards,

# Reviewer report: Reviewer response for version 1

**DOI:** 10.5256/f1000research.189692.r435300

**Published:** 2025-12-30

**Authors:** Rashmi Khazanchi

**Affiliations:** 1 Open University of the Netherlands, Heerlen, The Netherlands

The manuscript titled *“Closing the Gap in Early Mathematics: Domain and Cognitive Insights from TIMSS 2023 in South Africa and Singapore”* addresses an important topic by examining gaps in elementary-level mathematics learning through a comparison of TIMSS results from the two countries. While the study’s focus and findings are potentially valuable, the manuscript contains several major methodological and conceptual issues that need to be addressed.

 The author compares TIMSS results of Grade 4 learners from Singapore with Grade 5 learners from South Africa; however, TIMSS assesses learners at Grades 4 and 8 only. The manuscript therefore needs to clarify how Grade 5 results were derived from TIMSS data and justify the rationale for benchmarking Grade 4 and Grade 5 learners. The research Question 1 may need to be revised, as TIMSS does not directly assess Grade 5 learners.

 Several in-text citations are missing throughout the manuscript as statement should be supported with appropriate citations. On page 3, the title of the literature review (“Comparative analysis of South African and Singaporean Grade 5 mathematics achievement”) requires correction and should be revised consistently throughout the paper, as it implies that the comparison is limited solely to Grade 5 mathematics achievement.

 In the conceptual framework section, the author introduces an integrative framework combining a comparative lens, the TIMSS curriculum model, and curriculum theory dimensions; however, the sentence presenting this framework is a paragraph long and reduces clarity. To enhance clarity, the author needs to explain the role of each framework component, articulate the directional relationships among key constructs, and strengthen the narrative link to Figure 1.

 In the results section, findings should be discussed in direct relation to the research questions rather than beginning with tables or figures. The discussion section would benefit from further elaboration and stronger integration with the literature reviewed earlier, as well as more explicit connections to the research questions to enhance clarity and readability.

Is the work clearly and accurately presented and does it cite the current literature?

Yes

If applicable, is the statistical analysis and its interpretation appropriate?

Partly

Are all the source data underlying the results available to ensure full reproducibility?

Yes

Is the study design appropriate and is the work technically sound?

No

Are the conclusions drawn adequately supported by the results?

Partly

Are sufficient details of methods and analysis provided to allow replication by others?

Partly

Reviewer Expertise:

Artificial Intelligence in Education, AI - based tutoring systems, Mathematics Achievement, Student Engagement.

I confirm that I have read this submission and believe that I have an appropriate level of expertise to state that I do not consider it to be of an acceptable scientific standard, for reasons outlined above.

# Response

**Published:** 2025-12-30

**Authors:** Mathelela Steyn Mokgwathi

**Affiliations:** Early Childhood Education, University of South Africa, Pretoria, Gauteng, South Africa

Dear Reviewer,

 Thank you for your careful reading of the manuscript and for the detailed and constructive feedback provided. I appreciate the time and scholarly attention devoted to the review, and I am grateful for the suggestions, which have been invaluable in strengthening the clarity, methodological transparency, and overall quality of the paper. I respond to each of the major points raised below.

 With regard to the comment concerning the comparison between Grade 4 learners in Singapore and Grade 5 learners in South Africa, I appreciate the opportunity to clarify this important methodological issue. While TIMSS is internationally benchmarked at Grades 4 and 8, it is also well documented that several participating countries, including South Africa, assess learners at Grade 5 using the Grade 4 TIMSS assessment instruments. This approach is adopted where curriculum alignment indicates that the Grade 4 framework more accurately reflects the instructional exposure of learners, a practice that parallels the assessment of Grade 9 learners using the Grade 8 TIMSS instruments in some contexts. In the revised manuscript, I will make this rationale explicit by clarifying that the South African Grade 5 learners were assessed using the standard TIMSS Grade 4 mathematics framework and instruments, thereby ensuring construct equivalence and international comparability. In response to the reviewer’s suggestion, Research Question 1 will be refined to explicitly reflect this assessment design and to avoid any implication that TIMSS directly assesses a distinct Grade 5 framework.

 Concerning the observation that several in-text citations are missing, I acknowledge this limitation. A thorough review of the manuscript is currently underway to ensure that all empirical claims and theoretical statements are appropriately supported by relevant and up-to-date scholarly sources. These revisions will improve academic rigour and align with best practices in scholarly writing.

 Regarding the comment on the literature review title on page 3, I appreciate the reviewer’s attention to precision in terminology. The study intentionally focuses on Grade 5 learners in South Africa, assessed using the Grade 4 TIMSS instrument, and compares their performance with Grade 4 learners in Singapore, assessed using the same framework. To avoid ambiguity, the literature review title and related headings will be revised for consistency and clarity, ensuring that they accurately reflect the comparative focus and assessment design of the study, rather than implying a narrow or exclusive emphasis on Grade 5 achievement alone.

 I am also grateful for the insightful feedback on the conceptual framework section. I acknowledge that the current presentation of the integrative framework is overly dense. In the revised manuscript, this section will be restructured to clearly explain the role of each component, namely the comparative education lens, the TIMSS curriculum model, and the curriculum theory dimensions. The directional relationships among the key constructs will be explicitly articulated, and the narrative will be more tightly aligned with Figure 1 to enhance conceptual coherence and reader accessibility.

 Finally, I appreciate the guidance regarding the results and discussion sections. In response, the results will be reorganised so that the findings are presented explicitly in relation to the research questions before introducing supporting tables and figures. The discussion section will be expanded to provide deeper engagement with the literature reviewed earlier in the manuscript and to draw clearer, more explicit links between the findings, the research questions, and the broader scholarly debates on early mathematics achievement and international benchmarking.

 Once again, I sincerely thank the reviewer for these constructive comments. I am confident that addressing these points will substantially strengthen the manuscript and enhance its contribution to the literature on comparative mathematics education.

# Response

**Published:** 2026-01-01

**Authors:** Mathelela Steyn Mokgwathi

**Affiliations:** Early Childhood Education, University of South Africa, Pretoria, Gauteng, South Africa

I would like to thank Reviewer 1 for highlighting critical concerns regarding grade-level comparability, clarity of the benchmarking rationale, citation completeness, and alignment between the research questions, results, and interpretation. These issues have been carefully addressed through clarifications of TIMSS grade participation, revisions to the conceptual framing and research questions, strengthened citation support, and restructuring of the Results and Discussion sections. We believe the revised manuscript now fully addresses the raised concerns.

# Response

**Published:** 2026-04-15

**Authors:** Mathelela Steyn Mokgwathi

**Affiliations:** Early Childhood Education, University of South Africa, Pretoria, Gauteng, South Africa

Dear Dr Khazanchi,

 I would like to thank you for your constructive and insightful feedback, which has significantly strengthened this manuscript. In this revision, I have clarified the rationale for benchmarking South African Grade 5 learners with Singaporean Grade 4 learners by explicitly explaining the use of the TIMSS Grade 4 framework and ensuring measurement equivalence. I have also refined the research questions to align with the TIMSS design and streamlined the conceptual framework to improve clarity and coherence.

 In addition, I have strengthened the literature support throughout the manuscript, revised section headings for consistency, and restructured the Results and Discussion sections to ensure clear alignment with the research questions and stronger integration with existing literature. The conclusion has been rewritten to provide a concise synthesis fully grounded in the findings.

 I trust that these revisions address the concerns raised and improve the overall clarity, rigour, and contribution to the study.

 Kind regards,
